# Real-life prescribing of asthmatic treatments in UK general practice over time using 2014 BTS/SIGN steps

**DOI:** 10.1038/s41533-019-0137-7

**Published:** 2019-07-11

**Authors:** Alicia Gayle, Abigail Tebboth, Marie Pang, Florent Guelfucci, Ramzi Argoubi, Steven Sherman, Vincent Mak

**Affiliations:** 1 Boehringer Ingelheim, Bracknell, UK; 2 Creativ-Ceutical, Paris, France; 3 Creativ-Ceutical, Chicago, IL USA; 4 Imperial College Healthcare Trust, London, UK

**Keywords:** Asthma, Outcomes research

## Abstract

The 2014 British Thoracic Society (BTS) and Scottish Intercollegiate Guideline Network (SIGN) guidelines recommend a stepwise approach to asthma management. We investigated the management of asthma in primary care in the UK to understand how real-world practice compares with BTS/SIGN guidelines. Asthma patients were identified from the UK Clinical Practice Research Datalink from September 2006 to August 2016. Aims were to classify patients according to BTS/SIGN steps, describe the proportion of patients transitioning between steps and describe patient demographics and clinical characteristics per group. Overall, 647,308 patients with asthma were identified (40,096 aged 5–11 years; 607,212 aged 12–80 years). Most treated patients were in step 1 or 2 (88.3% of children/67.5% of adults in December 2007; 83.0% of children/67.0% of adults in June 2016). Most patients remained within their treatment step within a 6-month interval (>78% of children and adults throughout the study duration). The proportion of patients stepping up and down reduced from the beginning of the study, although stepping down to step 1 was relatively common in both adults and children. Few patients had a recorded asthma review in the year before reference date (18.8% of children and 14.8% of adults). Although prescribing patterns meant that most patients remained within their treatment step throughout the study, we cannot be sure that this was because their disease was truly stable. The small proportion of patients stepping up/down and the lack of recorded asthma review suggest that patients may not be treated in accordance with BTS/SIGN guidelines.

## Introduction

Asthma is one of the most common long-term conditions in the UK, with around 5.4 million people receiving treatment for asthma.^1^ Despite advances in treatment and clinical guidelines, there are still a number of patients for whom asthma control is inadequate, resulting in an estimated 100,000 inpatient episodes for asthma and over 1,000 asthma deaths a year.^2^ This problem was highlighted recently by the 2014 National Review of Asthma Deaths, which found potentially preventable factors in two-thirds of the deaths recorded.^3^ Specifically, potentially avoidable factors related to the non-implementation of British Thoracic Society (BTS)/Scottish Intercollegiate Guideline Network (SIGN) guidelines were found in 46% of deaths recorded.^3^ This highlighted the potential impact of lack of adherence to guideline recommendations on patient outcomes, although it was not an epidemiological study and did not reflect all UK asthma deaths. Prescribing habits were further scrutinised in a 2015 Asthma UK report, which found evidence of unsafe prescribing errors from over 500 UK general practices.^4^ Previous studies of asthma prescribing in the UK have reported discrepancies between real-world practice and guideline recommendations, although these are becoming outdated and have a greater focus on prescribing in paediatric patients.^5–10^ An updated understanding of how adults and children with asthma are treated in UK primary care, including how prescribing patterns compare with national guidelines, may enable us to identify key issues preventing optimal pharmacological treatment and, ultimately, improve asthma control.

The 2014 BTS/SIGN guidelines recommended a treatment escalation/de-escalation approach to the management of asthma, categorising patients into ‘steps’ based on the treatment prescribed (https://www.brit-thoracic.org.uk/quality-improvement/guidelines/asthma/).^11^ Steps are defined as: 1: mild intermittent asthma; 2: regular preventer therapy; 3: initial add-on therapy; 4: persistent poor control; 5: continuous or frequent use of oral steroids. According to these guidelines, patients should start treatment at the step most appropriate to the initial severity of their disease, stepping up as necessary and stepping down when control is good.

Recent studies have provided a ‘snapshot’ into the management of asthma patients in UK clinical practice,^8,12,13^ but there have been no studies looking at prescribing patterns vs. BTS/SIGN guidelines over a long period, including how patients transition between steps. We conducted a retrospective, observational, longitudinal study of asthma patients treated in primary care in the UK. The aims of this study were to classify patients according to BTS/SIGN guideline steps, describe the proportion of patients transitioning between steps and describe the patient demographics and clinical characteristics per group.

## Results

A total of 647,308 patients with a Read code for asthma diagnosis were identified. Of those, 40,096 were aged 5–11 years (paediatric group) and 607,212 were aged 12–80 years (adult group) at study inclusion (Supplementary Fig. 1). For overall patient demographics and clinical characteristics, see Supplementary Table 1.

The patients in the paediatric group had an average age of 8.7 years at study inclusion, and 60.1% were male (Supplementary Table 2). Eczema was the most common comorbidity (18.3%), followed by hayfever (9.8%). The number of children with any record of spirometry was generally low (2.2% of the whole group), but varied across the different steps with children in step 5 having the most recorded. Only a small number of children had a recorded asthma review in the year prior to reference date (18.8% with a record of review vs. 81.2% without). This was true for children in all BTS/SIGN steps: although there was a slight increase in review from steps 1 to 3, this decreased again in steps 4 and 5. A greater proportion of children had a recorded review in the 2 years prior to reference date, although the number with a record of review was still considerably lower than the number without review (33.4 vs. 66.6%).

Patients in the adult group had an average age of 43.04 years at study inclusion, and 44.9% were male (Supplementary Table 3). In all, 38.7% were recorded as current smokers, compared with 13.3% recorded as ex-smokers and 48.0% as non-smokers. Hayfever was the most common comorbidity (16.8%), followed by eczema (13.0%). Similar to the paediatric group, the number of patients with a record of spirometry was generally low (18.3% of the whole group), but with considerable variation across the steps (from 6.4% in step 1 to 36.3% in step 5). Again, only a small number of patients had a recorded asthma review in the year prior to reference date (14.8% with a record of review vs. 85.2% without). This was true across all BTS/SIGN steps: although there was a slight increase in review from steps 1 to 3, this decreased again in steps 4 and 5. More patients had a recorded review in the 2 years prior to reference date (26.7 vs. 73.3%), but the number of patients without a record of review still outnumbered those with review.

### Distribution of patients according to treatment steps

**Fig. 1:**
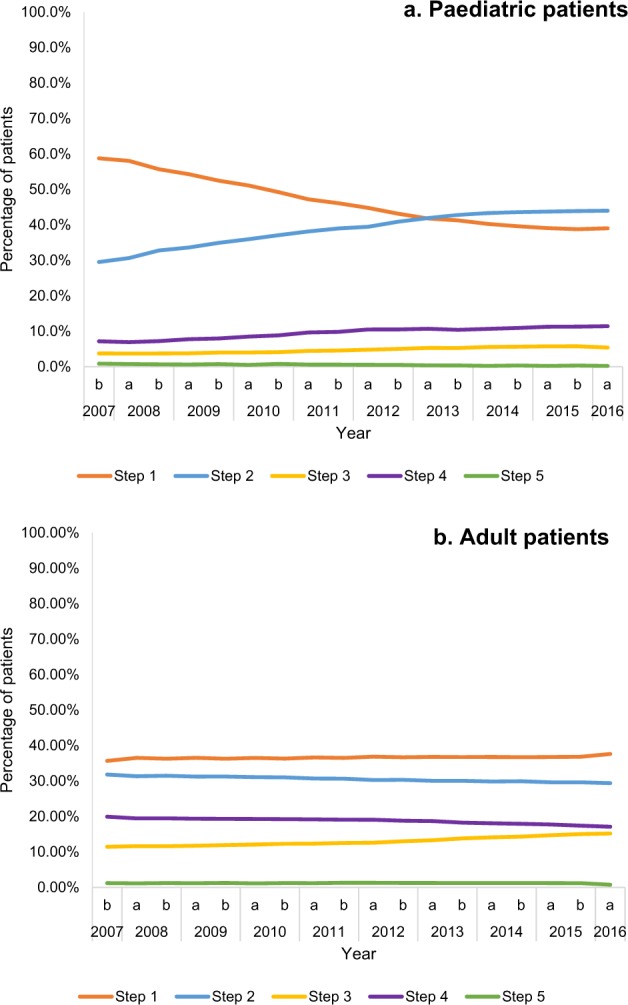
Distribution of patients in treatment steps, at each 6-month time interval

The most common steps in the paediatric group were steps 1 and 2 (Supplementary Table 5). A small number of children (3.2–5.2%) were diagnosed, but untreated; these were categorised as step 0. Of those who were treated, between 58.7 and 39.0% were in step 1 within a 6-month period, from the beginning to the end of the study (Fig. 1). The proportion of patients in step 2 was 29.5–44.0%. Only a very small proportion of children were in steps 3 and 5, and this was consistent throughout the study (3.7–5.4% and 0.9–0.2% of the treated patients over the study period, respectively). The proportion of children in step 4 was low at the start, but increased as the study went on (7.2–11.4% of the treated patients over the study period).

**Fig. 2:**
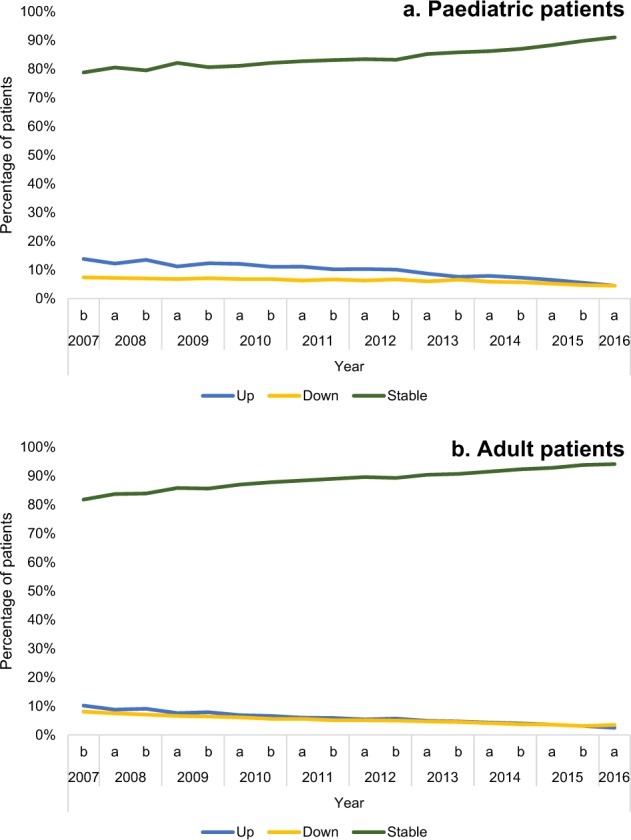
Proportion of patients stepping up, down or remaining stable within a 6-month period (patients in steps 0 to 5)

**Fig. 3:**
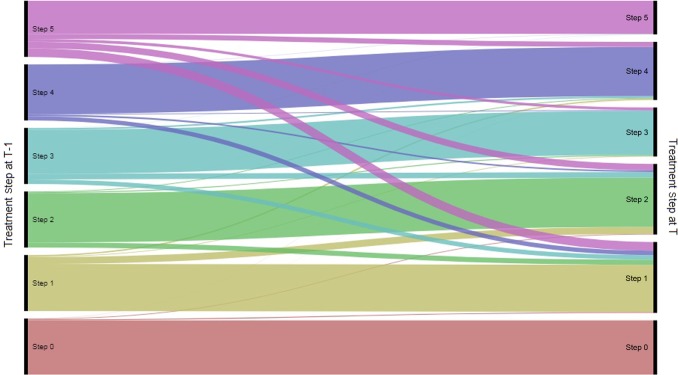
Flow of paediatric patients between steps from one 6-month interval to the next

A large proportion of patients in the paediatric group (≥78.8%) remained within their treatment step within a 6-month period (Fig. 2). The number of children stepping up reduced from the beginning to the end of the study (13.8% in December 2007 to 4.5% in June 2016). The number of children stepping down also decreased (7.4% in December 2007 to 4.5% in June 2016). Most children remained within the same treatment step from one 6-month interval to the next, regardless of the treatment step (Table 1, Fig. 3). Step 5 was the least stable treatment step, with 59.1% remaining in it from one 6-month interval to the next. Among the patients in step 5, the most common change in step was a non-consecutive step down from step 5–1 (15.5%). Among patients in step 4 and 2, it was a step down to step 1 (8.0 and 9.1%, respectively). For patients in steps 3 and 1, it was a move to step 2 (10.1 and 12.3%, respectively).

**Table 1:** Transitions from one interval (T-1) to the next (T) by treatment step

Paediatric patients		To: (Time interval T)				
		Step 5	Step 4	Step 3	Step 2	Step 1
From: (time interval T-1)	Step 5	**59.1%**	9.0%	4.9%	11.6%	15.5%
	Step 4	0.3%	**87.9%**	1.2%	2.7%	8.0%
	Step 3	0.3%	2.9%	**78.6%**	10.1%	8.1%
	Step 2	0.1%	1.3%	1.6%	**87.9%**	9.1%
	Step 1	0.1%	2.4%	1.1%	12.3%	**84.0%**
	Step 0	0.0%	0.2%	0.1%	1.2%	1.8%
Adult patients		To: (time interval T)				
		Step 5	Step 4	Step 3	Step 2	Step 1
From: (time interval T-1)	Step 5	**76.8%**	12.0%	4.1%	2.0%	5.2%
	Step 4	0.7%	**91.4%**	1.9%	0.8%	5.2%
	Step 3	0.3%	2.2%	**90.3%**	1.8%	5.4%
	Step 2	0.1%	0.9%	1.4%	**89.9%**	7.6%
	Step 1	0.2%	2.7%	2.1%	6.9%	**88.1%**
	Step 0	0.0%	0.5%	0.3%	2.5%	4.3%

This table summarises how patients transition between BTS/SIGN steps from one time interval (T-1) to the next time interval (T). For example, 9.0% of paediatric patients in step 5 in the previous time interval (T-1) had moved to step 4 by time interval T. Similarly, 5.4% of adult patients in step 3 in the previous time interval (T-1) had moved to step 1 by time interval T

Bold formatting indicates the most common transition in each step

As in the paediatric group, most patients in the adult group were in steps 1 and 2 (Supplementary Table 6). In all, 12.0% patients were diagnosed but untreated (step 0) at the start of the study; this had reduced to 6.4% by the study end. Of the treated patients, 35.7–37.6% were in step 1 from the beginning to the end of the study (Fig. 1). The proportion of patients in step 2 was 31.8–29.4%. There were more patients in steps 3 and 4 compared with the paediatric population (step 3: from 11.4% in 2007 to 15.2% in 2016; step 4: from 19.9% in 2007 to 17.1% in 2016). However, the number of patients in step 5 was very low throughout the study (from 1.2% in 2007 to 0.7% in 2016).

A large proportion of adult patients (>80%) remained within their treatment step within a 6-month period (Fig. 2). The number of patients stepping up reduced over the course of the study (10.2% in December 2007 to 2.5% in June 2016). Similarly, the number of patients stepping down decreased over time from 8.1% in December 2007 to 3.5% in June 2016.

**Fig. 4:**
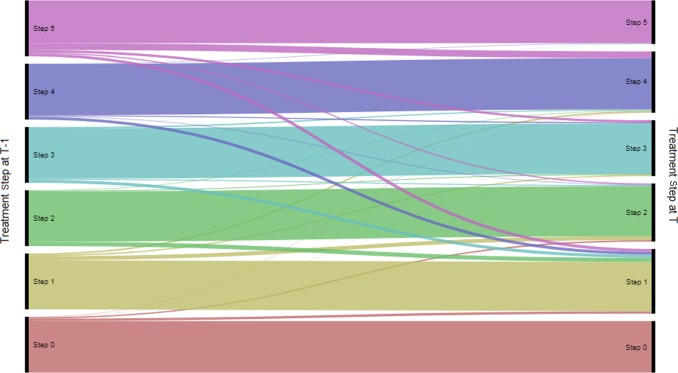
Flow of adult patients between steps from one 6-month interval to the next

Most adult patients remained within their treatment step from one 6-month interval to the next (Table 1, Fig. 4). Step 5 was the least stable, with 76.8% remaining in this treatment step from one 6-month interval to the next. Among patients in step 5, the most common change of step was from steps 5 to 4 (12.0%). Among patients in steps 4, 3 and 2, it was a step down to step 1 (5.2, 5.4 and 7.6%, respectively); for patients in step 1, it was a step up to step 2 (6.9%). Stepping down from step 5 to step 1 was also relatively common (5.2%).

### Sensitivity analysis

Sensitivity analyses were conducted to investigate the impact of certain assumptions on the results (further details in the Methods section). Firstly, we changed the interval length from 6 months to 3 months. Overall, this had a low impact on the direction of results in children and adults (Supplementary Tables 7, 8), with similar proportions of patients in each treatment step compared with the base case. There was an increase in the number of patients remaining within a step within a time interval, but a reduction in the number of patients stepping up or down that was most noticeable at the beginning of the study (Supplementary Tables 9, 10). More patients also remained in their step from one 3-month interval to the next in both the adult and paediatric groups (Supplementary Tables 11, 12).

Changing the length of the grace period from 90 days to 30 days had a low impact on the results in children, with a distribution of patients across treatment steps that was identical to the base case (Supplementary Tables 13, 14). Most children remained within their treatment step within a 6-month interval, and a similar proportion stepped up or down within a 6-month interval compared with the base case (Supplementary Table 15). Overall, the pattern of treatment transitions from one 6-month interval to the next was similar to the base case, but with a slight increase in the number of children stepping down to step 1 from all other steps (Supplementary Table 16). The change in grace period also had a low impact on the overall results in the adult group with a distribution of patients across steps that was identical to the base case. There was a small decrease in the number of patients remaining in their treatment step within a 6-month period and a slight increase in the numbers stepping up, although this was a very small change compared with the base case (Supplementary Table 17). Most patients also remained in their treatment step from one 6-month interval to the next, with a slight increase in the number of patients stepping down to step 1 from all other steps compared with the base case (Supplementary Table 18).

When paediatric patients with comorbid COPD were excluded from the analysis (0.2% of the total paediatric population) there was very little change to the results, with almost identical distribution across treatment steps vs. the base case (Supplementary Table 19). There was effectively no change in the number of children remaining in a treatment step, or stepping up or down, from one 6-month interval to the next (Supplementary Tables 20, 21). Similarly, excluding adult patients with comorbid COPD from the analysis (6.8% of the total adult population) had a low impact on the results. Although there was a slight increase in the number of patients in steps 0–2 and a slight decrease in the number of patients in steps 3–5, these differences were very small and there were similar proportions of patients in each treatment step vs. the base case (Supplementary Table 22). Again, most patients remained within their treatment step, and there was little change in the number of patients stepping up or down from one 6-month interval to the next (Supplementary Tables 23, 24).

Finally, widening the definition of asthma review to include all Read codes in the QOF business rules (Supplementary Table 25) had little effect on the results in the paediatric or adult groups. When all asthma review codes were included, the number of children with a record of review in the year prior to reference date increased slightly (21.0% with review vs. 18.8% in the base case; Supplementary Table 26). For the 2 years prior to reference date, the proportion with review increased to 36.8 vs. 33.4% in the base case. The results were very similar in adults: 16.4% had review in the year prior to reference date vs. 14.8% in the base case and 29.1% had review in the 2 years prior to reference date vs. 26.7% in the base case (Supplementary Table 27).

## Discussion

We designed this study of UK patients with asthma to understand how real-world patients are managed in primary care, including the distribution of patients across BTS/SIGN steps, how regularly patients move between steps and whether the treatment approach generally follows guideline recommendations. These guidelines recommend a stepwise approach to the management of asthma, with patients starting treatment at the step most appropriate to the initial severity of their disease.^11^ Stepping up and down is recommended as needed, with regular review of patients as treatment is stepped down. Therefore, we would expect that patients move up and down consecutive treatment steps, as dictated by regular review.

The majority of the treated study population (adults and children) were in steps 1 and 2. This is similar to previous studies, for example a recent analysis published by Bloom et al.^12^ Our step 1 patients were only treated with short-acting bronchodilators, but according to the treatment algorithm in the 2016 BTS/SIGN guidelines, patients should start treatment with an inhaled corticosteroid (ICS) at an earlier stage.^14^ Although it was outside of the scope of this study, it would be interesting to follow this group of patients to see if, and how, prescribing patterns change based on these new recommendations.

The majority of patients in both groups remained in their treatment step both within a 6-month interval and from one 6-month interval to the next. This was even more noticeable when the interval was reduced from 6 to 3 months. Similarly, the number of patients stepping up and down was relatively low and reduced throughout the study. However, the number of patients with a record of asthma review in the year or 2 years prior to reference date was very low, including patients in steps 4 and 5. This raises the question: are patients remaining within a treatment step because their disease is truly stable, or because of clinical inertia? It is possible that patients in steps 4 and 5 are being reviewed in secondary care, and that this is not being reported to general practice. It is also possible that patients across all steps are being reviewed but not having this recorded, although this seems unlikely as it would result in loss of payments for GP practices under the UK Quality and Outcomes (QOF) framework. QOF-registered practices reported an average of 71.94% patients receiving asthma review in the preceding 12 months in 2016/17, highlighting a clear disconnect between what is reported in QOF and what observational data from CPRD suggests.^15^ The low numbers of patients stepping up and down may be a result of healthcare professionals’ clinical concerns. For example, they may be reluctant to step-up therapy due to awareness of safety issues with high-dose steroids, or reluctant to step-down therapy due to concerns about leaving patients inadequately controlled or at risk of exacerbations.^16,17^ It is also possible that clinicians are conducting asthma reviews without properly considering whether patients are adequately controlled on the current therapy, or if they should be stepped up or down. It is difficult to speculate on this point, given the descriptive nature of our study, however, the lack of recorded review is suggestive of at least sub-optimal data recording, if not a lack of sufficient asthma review in the real world. In addition, the number of patients with a record of spirometry or peak flow assessment was low in both adults and children (Supplementary Tables S2, S3). BTS/SIGN guidelines recommend that lung function, assessed by spirometry or peak expiratory flow, is monitored and recorded in adults in primary care. Spirometry is also the preferred initial test to assess the presence and severity of airflow obstruction in adults when diagnosing asthma. The low number of patients with a record of spirometry suggests that patients are being diagnosed without spirometry, or that spirometry is not being recorded. In combination with the low number of patients with a record of asthma review, this further supports the conclusion that either data are not well recorded, or patient management differs from BTS/SIGN guideline recommendations.

In the paediatric group, non-consecutive and consecutive steps were equally common (0.1–15.5% and 0.3–12.3%, respectively), and stepping down to step 1 from all other steps (particularly step 5) was a relatively common change. This suggests that paediatric patients do not always move through the treatment pathway in a stepwise manner. The most common non-consecutive steps were steps down (for example step 5–1, step 5–2, step 3–1 and step 4–1), suggesting that non-stepwise management is driven by step down, rather than step up. Although it is a relatively small number of patients, it is potentially concerning that children receiving step 5 therapy are being reduced from high dose ICS to no ICS, a strategy that is not in line with current guidelines. We also saw an increase in the number of patients in step 2 as the study progressed, with this becoming the most common step in the paediatric group by the end of the study. As the study design means that the average age of the population increased throughout the study duration, this may reflect an increase in confidence in prescribing ICS to children as they get older, although our study was not powered to detect differences by age within the cohorts. The adult group showed more stepwise movement than the paediatric group, with consecutive steps being more common than non-consecutive steps. However, as in the paediatric population, there seemed to be a preference for stepping down, particularly to step 1. The preference for stepping down to step 1, where patients are no longer receiving maintenance therapy, is interesting. This could reflect prescribers’ intentions, or it could indicate that asthma patients stop requesting repeat prescriptions for maintenance therapy and only request reliever therapy once they are no longer symptomatic. Our study was not able to capture the impact of repeat vs. new prescriptions, so we can only speculate on this point, although previous research has noted a reluctance to use maintenance therapy among asthma patients when symptoms were absent, which would fit with this suggestion.^18^ The time period of 6 months used to assign treatment steps was chosen to capture all likely ways in which patients could be prescribed combinations of therapy; therefore the multiple steps down seen are likely to reflect clinical practice, rather than being a result of data recording. It would be interesting to follow patients who stepped down multiple steps at a time to see if they experienced worse outcomes, or if they remained stable afterwards. However, this was outside the scope of our current study.

Step 4 was the third most common step in both adults and children, representing around 11% of the treated children and 20% of the treated adults. In the paediatric group, the number of patients in step 4 increased throughout the study period. It would be interesting to see if this trend continues now that tiotropium is approved for asthma patients over 6 years, as previous BTS/SIGN recommendations for tiotropium were based on off-label use. Step 5 was far less common, potentially highlighting concerns around continuous or frequent use of steroids.

Overall, our findings suggest that most patients remain stable within their treatment step for a considerable length of time, with few patients stepping up or down. However, less than a third of patients had a recorded asthma review in the year prior to reference date, despite QOF-reported rates being around 72%.^15^ Therefore, it is possible that the lack of movement between steps is due to patients not having regular asthma review, rather than good asthma control being achieved. This has implications for primary care physicians in the United Kingdom, highlighting a disconnect between what is reported in QOF and what is observable in a real-world database. It also emphasises the need to improve asthma review rates and to give greater consideration to prescribing in these sessions, which may help practitioners improve asthma control for their patients.

This study was conducted using data from the CPRD. Whilst this database strives for high data quality, this relies on accurate data entry by health professionals. It is therefore possible that there may be missing values where data have not been recorded, for example diagnosis of asthma. However, this is unlikely to have a significant impact on the results, as research has shown that people with asthma can be accurately identified from CPRD using Read codes.^19^ Other missing data, such as patient demographics and comorbidities, are not expected to affect our results as these were not being directly studied. It is not possible to obtain secondary care prescribing data, and the focus on primary care only may not capture all patients, as those with more severe disease are likely to be managed in specialist care. However, since prescriptions for most patients should be recorded in general practice the impact of this should be minimal.

This study did not attempt to capture whether patients were compliant to treatment or not, but was instead meant to investigate prescribers’ intention to treat. This is reflected in the study design, for example the use of a long grace period and consideration of periods of discontinuation as part of the step before discontinuation. As with all studies in CPRD, we cannot know if the prescription was collected or whether the patient took the medication. Although, since the initial period of prescription within 180 days was used as a qualifying measure, the likelihood of patients collecting their medication is higher. Lastly, our use of 6-month time windows means that we could overestimate stability by missing patients who change step within these time periods. The impact of this is likely to be low, however, as our sensitivity analysis using 3-month time windows showed very little difference from the base case.

The majority of patients in this study were in steps 1 and 2; most remained within their treatment steps with only a small proportion stepping up or down in the intervals studied. In addition, the proportion of patients with a recorded annual asthma review was low. Together, these findings suggest that a large group of patients may remain in their treatment step due to lack of review, rather than good disease control, and thus that they are potentially not being managed in accordance with BTS/SIGN guidelines. Step 4 was the third most common step in both adults and children, representing around 11% of the treated children and 20% of the treated adults.

## Methods

### Study design

This was a retrospective non-interventional cohort study using data from the UK Clinical Practice Research Datalink (CPRD). The CPRD contains primary care medical records from more than 738 UK GP practices, including diagnoses, prescriptions, referrals and test results. The geographical distribution covers the whole of the UK and is representative of the UK population.^20^ It is a recognised source of primary care-level data for epidemiological studies in the UK, and has been used in over 2,000 publications to date (https://www.cprd.com/home/).

### Population

The population of interest was patients with an active diagnosis of asthma registered at a practice designated as ‘up to standard’ by the CPRD during the study period (1st September 2006 and 31st August 2016). Patients had to have all records classed as ‘acceptable’ by the CPRD, as well as being aged between 5 and 80 years by the reference date (1st June 2016). ‘Acceptable research standards’ and ‘up to standard’ are metrics provided by CPRD to give a measure of the suitability of patient records for research, based on the quality of the patient record and the practice’s data recording. We also required a minimum period of 12 months continuous enrolment in the database prior to the reference date in order to assess treatment patterns and covariates of interest. Patients participating in clinical trials or asthma studies during the study period were excluded.

### Analysis

Patients were categorised into two sub-cohorts following selection, according to their age at reference date. These groups were referred to as children (aged 5–11 years) and adults (aged 12–80 years). This threshold was chosen as an interpretation of the BTS/SIGN guideline, which defines adults as >12 years, and in line with the dosing thresholds for children/adults specified by certain asthma medications (e.g., tiotropium and beclometasone dipropionate). The sub-cohorts were then assigned to five treatment categories, corresponding to the five steps of the 2014 BTS/SIGN guidelines, at 6-month intervals (Supplementary Table 4). We used the 2014 BTS/SIGN guidelines, as although a more recent version was published in 2016; these would not have been available for the majority of the period covered by our study (2006–2016). In addition, a ‘step 0′ was also defined for patients who had a record of asthma diagnosis, but no prescribed asthma treatment. Patients who had their first asthma diagnosis during the study period were classed as ‘undiagnosed’ for the period before their diagnosis; after this, they were included in the relevant treatment step(s) for the duration of the study. Step assignment was based on an algorithm that converted all prescriptions of asthma treatments into a set of treatment sequences (i.e., consecutive prescriptions of the same treatment or combination of treatments, without a gap exceeding a defined period between prescriptions; Supplementary Fig. 2). Assumptions were made to identify the treatment steps based on the defined treatment sequences. These are listed below.

- Grace period: A grace period of 90 days was required between two consecutive prescriptions from the same drug category before this was considered to be a discontinuation.
- Overlap period: A minimum overlap period of 30 days’ prescription of two different asthma drug categories was required for those categories to be considered as being combined.
- Rescue packs: An oral prednisolone prescription of less than 2 weeks’ duration using ≥5 mg strength tablets was considered as a prescription of rescue oral steroids to treat an exacerbation of asthma.^21^
- Continuous/frequent use of oral corticosteroids (OCS): Patients were considered as receiving continuous or frequent OCS if they had at least five consecutive prescriptions of rescue OCS over a period of 6 months and/or at least one prescription of a non-rescue OCS.

Once allocated to a treatment step, each patient was further described as one of the following: stepped up (patient stepped up to a more extensive therapy within a 6-month interval); stepped down (patient stepped down to a less extensive therapy within a 6-month interval); stable (patient remained in the same step of therapy within a 6-month interval). Socio-demographic characteristics of interest were age, gender, region and smoking status. Clinical characteristics of interest were: any spirometry in medical history, any home peak flow monitoring in medical history, peak flow record in last 12 months, best peak flow from July 2015 to August 2016, asthma annual review between August 2015 and August 2016 (yes/no), annual asthma review between August 2014 and August 2016 (yes/no), any record of COPD, hayfever, eczema, rhinosinusitis and eosinophil count (yes/no and value recorded).

Comorbidities and demographic information were analysed descriptively for the two age groups at the different steps, and comparisons between steps were assessed using chi-squared or *t* tests as appropriate. Treatment transitions were assessed retrospectively; patients’ steps were assigned at 6-monthly time points and left-censored at the beginning of the study period or loss of follow-up/first registration date with a practice (whichever occurred first). The proportion of patients stepping up, down or remaining stable was assessed descriptively at each of the 18 time points prior to the reference date.

We also conducted sensitivity analyses, including repeating the categorisation of patients as stepping up, down or remaining stable using a 3-month time period. This was compared with the analysis conducted using a 6-month period to determine the importance of the time frame selected. A second sensitivity analysis excluded patients with any diagnosis of COPD in order to assess any potential confounding effects on prescribing patterns. We also repeated the base-case analysis with a reduced grace period of 30 days. Finally, we included a wider range of Read codes for asthma review (Supplementary Table 25) to see if this changed the result vs. base case.

The data were extracted using CPRD-GOLD, the online version of CPRD, and analysed using SAS software, Version 9.3 (copyright © 2011 SAS Institute Inc.). SAS and all other SAS Institute Inc. product or service names are registered trademarks or trademarks of SAS Institute Inc., Cary, NC, USA. Any missing data or outlier values for dosage/strength and duration of prescription were corrected based on prescription and pack information available in the data sets. All Read, medical and product codes used to in the conduct of this study are available in the Supplementary Material (Supplementary Tables 28–50).

This study was approved by the Independent Scientific Advisory Committee for Medicines and Healthcare products Regulatory Agency database research (reference 17_214), in addition to a scientific committee within the study sponsor.

### Reporting Summary

Further information on research design is available in the Nature Research Reporting Summary linked to this article.

## Supplementary information

Supplementary Information

Reporting summary

## Data Availability

The data sets generated and analysed during this study are available from the corresponding author on reasonable request. The code used to define treatments steps is available in the Supplementary Material (see Supplementary Notes).

## Supplementary information

**Supplementary Information** accompanies the paper on the *npj Primary Care Respiratory Medicine* website (10.1038/s41533-019-0137-7).
