# Supplementary material for: Real-life prescribing of asthmatic treatments in UK general practice over time using 2014 BTS/SIGN steps
Source: NPJ Prim Care Respir Med. 2019 Jul 11;29:25. doi: 10.1038/s41533-019-0137-7 (PMC6624291; doi:10.1038/s41533-019-0137-7)
Supplement: Supplementary file 1 — Supplementary Information [file 41533_2019_137_MOESM1_ESM.docx]

**Supplementary Information**

# Supplementary Figures

Supplementary Figure 1: Patient selection flow chart


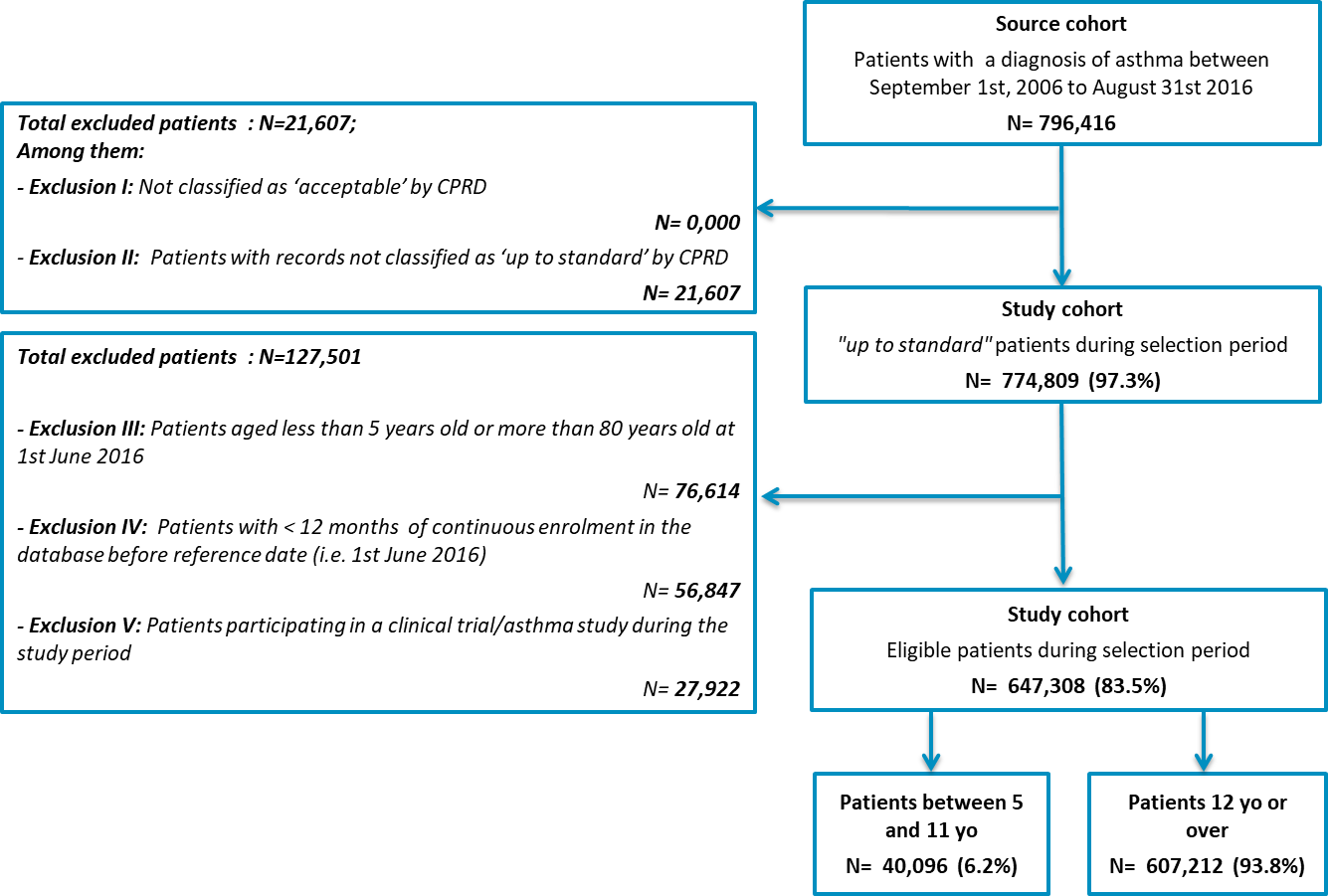


Abbreviations: CPRD, Clinical Practice Research Datalink; yo, years old

Supplementary Figure 2: Algorithm to assign treatment steps

# Supplementary Tables

Supplementary Table 1: Baseline characteristics of all patients at reference date

| **Characteristic** | | **All (N=647,308)** |
| --- | --- | --- |
| **Gender** | **Number of valid values** | **647,268 (100.0%)** |
|  | Male | 296,971 (45.9%) |
|  | Female | 350,297 (54.1%) |
| **Age at reference date** | **Number of valid values** | **647308 (100.0%)** |
|  | Mean (SD) | 40.91 (20.15) |
|  | 95% CI | [40.86 - 40.96] |
|  | Min-Max | [5.0 - 80.0] |
|  | Median | 40 |
|  | Q1-Q3 | [24.0 - 57.0] |
| **Age (categories)** | 5-12 years | 40,096 (6.2%) |
|  | 12-18 years | 56,135 (8.7%) |
|  | 18-40 years | 222,352 (34.4%) |
|  | 40-60 years | 188,331 (29.1%) |
|  | 60-80 years | 140,394 (21.7%) |
| **Region** | **Number of valid values** | **647,308 (100.0%)** |
|  | North East | 9,456 (1.5%) |
|  | North West | 70,047 (10.8%) |
|  | Yorkshire & The Humber | 16,803 (2.6%) |
|  | East Midlands | 17,985 (2.8%) |
|  | West Midlands | 59,963 (9.3%) |
|  | East of England | 55,374 (8.6%) |
|  | South West | 63,198 (9.8%) |
|  | South Central | 74,205 (11.5%) |
|  | London | 68,597 (10.6%) |
|  | South East Coast | 61,013 (9.4%) |
|  | Northern Ireland | 19,439 (3.0%) |
|  | Scotland | 59,089 (9.1%) |
|  | Wales | 72,139 (11.1%) |
| **Annual asthma review and tests** | **Number of valid values** | **647,308 (100.0%)** |
| Annual asthma review | Before 01/06/2016 | 508193 (78.5%) |
|  | 12 months before 01/06/2016 | 97644 (15.1%) |
|  | 24 months before 01/06/2016 | 175241 (27.1%) |
| Spirometry | Before 01/06/2016 | 112,164 (17.3%) |
|  | 12 months before 01/06/2016 | 12,354 (1.9%) |
| Peak flow | Before 01/06/2016 | 103,629 (16.0%) |
|  | 12 months before 01/06/2016 | 5,776 (0.9%) |
| Peak flow (best) | Before 01/06/2016 | 9,323 (1.4%) |
|  | 12 months before 01/06/2016 | 258 (0.0%) |
| **Comorbidities** | **Number of valid values** | **647,308 (100.0%)** |
| Hayfever | Before 01/06/2016 | 105,799 (16.3%) |
|  | 12 months before 01/06/2016 | 3,481 (0.5%) |
| Eczema | Before 01/06/2016 | 86,013 (13.3%) |
|  | 12 months before 01/06/2016 | 3,111 (0.5%) |
| Rhinosinusitis | Before 01/06/2016 | 2,057 (0.3%) |
|  | 12 months before 01/06/2016 | 133 (0.0%) |
| COPD | Before 01/06/2016 | 41,468 (6.4%) |
|  | 12 months before 01/06/2016 | 13,014 (2.0%) |
| **Follow-up duration** | | |
| Follow-up since date on enrolment in database | **Number of valid values** | **647,308 (100.0%)** |
|  | Mean (SD) | 5,996.47 (4,460.34) |
|  | 95% CI | [5,985.61 – 6,007.34] |
|  | Min-Max | [365.0 – 29,367.0] |
|  | Median | 4,851 |
|  | Q1-Q3 | [2,751.0 – 8,224.0] |
| Follow-up since first asthma diagnosis | **Number of valid values** | **645,192 (99.7%)** |
|  | Mean (SD) | 3,827.30 (2105.26) |
|  | 95% CI | [3,822.16 – 3,832.43] |
|  | Min-Max | [0.0 – 10,484.0] |
|  | Median | 3,716 |
|  | Q1-Q3 | [2,277.0 – 5,059.0] |
| Follow-up since first asthma prescription | Number of valid values | 625,944 (96.7%) |
|  | Mean (SD) | 4,065.48 (2,124.35) |
|  | 95% CI | [4,060.22 – 4,070.74] |
|  | Min-Max | [0.0 – 10,491.0] |
|  | Median | 3,905 |
|  | Q1-Q3 | [2,450.0 – 5,451.0] |

Abbreviations: CI, confidence interval; COPD, chronic obstructive pulmonary disease; SD, standard deviation

Supplementary Table 2: Baseline patient characteristics in the paediatric group (aged 5-11 years) at reference date

| **Characteristics** | | **Treatment step at reference date** | | | | | | |  |
| --- | --- | --- | --- | --- | --- | --- | --- | --- | --- |
|  |  | **Step 5** | **Step 4** | **Step 3** | **Step 2** | **Step 1** | **Step 0** | **Undiagnosed** | **All** |
|  |  | **N = 83** | **N = 4,312** | **N = 2,055** | **N = 16,690** | **N = 14,796** | **N = 2,081** | **N = 79** | **N = 40,096** |
| **Gender** | Male | 51 (61.4%) | 2,609 (60.5%) | 1,233 (60.0%) | 10,004 (59.9%) | 8,952 (60.5%) | 1,220 (58.7%) | 44 (55.7%) | 24,113 (60.1%) |
|  | Female | 32 (38.6%) | 1,703 (39.5%) | 822 (40.0%) | 6,686 (40.1%) | 5,844 (39.5%) | 860 (41.3%) | 35 (44.3%) | 15,982 (39.9%) |
| **Age** | Mean (SD) | 8.17 (1.87) | 8.93 (1.78) | 8.23 (1.94) | 8.55 (1.87) | 8.74 (1.85) | 9.16 (1.65) | 8.46 (1.95) | 8.68 (1.86) |
|  | 95% CI | [7.76; 8.58] | [8.88; 8.98] | [8.14; 8.31] | [8.52; 8.58] | [8.71; 8.77] | [9.09; 9.23] | [8.02; 8.89] | [8.66; 8.69] |
|  | Min-Max | [5.0; 11.0] | [5.0; 11.0] | [5.0; 11.0] | [5.0; 11.0] | [5.0; 11.0] | [5.0; 11.0] | [5.0; 11.0] | [5.0; 11.0] |
|  | Median | 8 | 9 | 8 | 9 | 9 | 10 | 8 | 9 |
|  | Q1-Q3 | [6.0; 10.0] | [8.0; 11.0] | [7.0; 10.0] | [7.0; 10.0] | [7.0; 10.0] | [8.0; 11.0] | [7.0; 10.0] | [7.0; 10.0] |
| **Monitoring** | Spirometry, any in medical history | 3 (3.6%) | 133 (3.1%) | 63 (3.1%) | 345 (2.1%) | 319 (2.2%) | 16 (0.8%) | 0 (0.0%) | 879 (2.2%) |
|  | Home peak flow monitoring | 3 (3.6%) | 376 (8.7%) | 163 (7.9%) | 1,270 (7.6%) | 986 (6.7%) | 31 (1.5%) | 0 (0.0%) | 2,829 (7.1%) |
|  | Peak flow record [last 12 months] | 2 (2.4%) | 76 (1.8%) | 37 (1.8%) | 265 (1.6%) | 196 (1.3%) | 4 (0.2%) | 0 (0.0%) | 580 (1.4%) |
|  | Asthma annual review [July 15- Aug 2016] | 7 (8.4%) | 1,065 (24.7%) | 616 (30.0%) | 3,449 (20.7%) | 2,387 (16.1%) | 11 (0.5%) | 0 (0.0%) | 7,535 (18.8%) |
|  | Asthma annual review [July 14- Aug 2016] | 18 (21.7%) | 1,854 (43.0%) | 984 (47.9%) | 6,204 (37.2%) | 4,276 (28.9%) | 54 (2.6%) | 0 (0.0%) | 13,390 (33.4%) |
|  | Eosinophil count | 12 (14.5%) | 693 (16.1%) | 341 (16.6%) | 2,043 (12.2%) | 1,905 (12.9%) | 166 (8.0%) | 10 (12.7%) | 5,170 (12.9%) |
| **Comorbidities** | COPD | 1 (1.2%) | 8 (0.2%) | 3 (0.1%) | 43 (0.3%) | 29 (0.2%) | 10 (0.5%) | 0 (0.0%) | 94 (0.2%) |
|  | Hayfever | 5 (6.0%) | 498 (11.5%) | 270 (13.1%) | 1,623 (9.7%) | 1,402 (9.5%) | 109 (5.2%) | 11 (13.9%) | 3,918 (9.8%) |
|  | Eczema | 8 (9.6%) | 818 (19.0%) | 377 (18.3%) | 3,162 (18.9%) | 2,677 (18.1%) | 289 (13.9%) | 11 (13.9%) | 7,342 (18.3%) |
|  | Rhinosinusitis | 0 (0.0%) | 3 (0.1%) | 1 (0.0%) | 5 (0.0%) | 4 (0.0%) | 0 (0.0%) | 0 (0.0%) | 13 (0.0%) |

Abbreviations: CI, confidence interval; COPD, chronic obstructive pulmonary disease; SD, standard deviation

Supplementary Table 3: Baseline patient characteristics in the adult group (aged 12-80 years) at reference date

| **Characteristics** | | **Treatment step at reference date** | | | | | | | |
| --- | --- | --- | --- | --- | --- | --- | --- | --- | --- |
|  |  | **Step 5** | **Step 4** | **Step 3** | **Step 2** | **Step 1** | **Step 0** | **Undiagnosed** | **All** |
|  |  | **N = 4,418** | **N = 96,956** | **N = 86,008** | **N = 167,143** | **N = 213,607** | **N = 38,586** | **N = 494** | **N = 607,212** |
| **Gender** | Male | 1,603 (36.3%) | 41,334 (42.6%) | 36,926 (42.9%) | 76,205 (45.6%) | 98,760 (46.2%) | 17,825 (46.2%) | 205 (41.5%) | 272,858 (44.9%) |
|  | Female | 2,814 (63.7%) | 55,618 (57.4%) | 49,080 (57.1%) | 90,936 (54.4%) | 114,836 (53.8%) | 20,742 (53.8%) | 289 (58.5%) | 334,315 (55.1%) |
| **Age** | Mean (SD) | 62.39 (15.43) | 53.33 (16.80) | 47.69 (18.49) | 40.06 (18.92) | 39.98 (18.34) | 34.49 (14.85) | 38.97 (18.42) | 43.04 (18.96) |
|  | 95% CI | [61.94; 62.85] | [53.22; 53.43] | [47.57; 47.82] | [39.97; 40.15] | [39.90; 40.06] | [34.34; 34.64] | [37.34; 40.60] | [42.99; 43.09] |
|  | Min-Max | [12.0; 80.0] | [12.0; 80.0] | [12.0; 80.0] | [12.0; 80.0] | [12.0; 80.0] | [12.0; 80.0] | [12.0; 80.0] | [12.0; 80.0] |
|  | Median | 67 | 55 | 49 | 38 | 38 | 31 | 38 | 42 |
|  | Q1-Q3 | [54.0; 74.0] | [41.0; 68.0] | [33.0; 63.0] | [23.0; 55.0] | [24.0; 53.0] | [24.0; 42.0] | [23.0; 54.0] | [27.0; 58.0] |
| **Smoking status** | **Number of valid values** | **4,377 (99.1%)** | **96,253 (99.3%)** | **84,446 (98.2%)** | **158,155 (94.6%)** | **202,875 (95.0%)** | **35,364 (91.6%)** | **443 (0.1%)** | **581,913 (95.8%)** |
|  | Current smokers | 1,877 (42.9%) | 43,970 (45.7%) | 34,022 (40.3%) | 51,511 (32.6%) | 80,536 (39.7%) | 13,401 (37.9%) | 122 (27.5%) | 225,439 (38.7%) |
|  | Ex-smokers | 968 (22.1%) | 16,291 (16.9%) | 13,061 (15.5%) | 19,852 (12.6%) | 23,254 (11.5%) | 3,828 (10.8%) | 68 (15.3%) | 77,322 (13.3%) |
|  | Non-smokers | 1,532 (35.0%) | 35,992 (37.4%) | 37,363 (44.2%) | 86,792 (54.9%) | 99,085 (48.8%) | 18,135 (51.3%) | 253 (57.1%) | 279,152 (48.0%) |
| **Monitoring** | Spirometry, any in medical history | 1,602 (36.3%) | 29,550 (30.5%) | 22,527 (26.2%) | 23,201 (13.9%) | 31,915 (14.9%) | 2,466 (6.4%) | 24 (4.9%) | 111,285 (18.3%) |
|  | Home peak flow monitoring | 707 (16.0%) | 18,334 (18.9%) | 16,866 (19.6%) | 29,979 (17.9%) | 32,609 (15.3%) | 2,302 (6.0%) | 3 (0.6%) | 100,800 (16.6%) |
|  | Peak flow record [last 12 months] | 19 (0.4%) | 886 (0.9%) | 1,130 (1.3%) | 1,657 (1.0%) | 1,471 (0.7%) | 30 (0.1%) | 3 (0.6%) | 5,196 (0.9%) |
|  | Peak flow (best) [July 15- Aug 2016] | 2 (0.0%) | 52 (0.1%) | 57 (0.1%) | 74 (0.0%) | 42 (0.0%) | 1 (0.0%) | 0 (0.0%) | 228 (0.0%) |
|  | Asthma annual review [July 15- Aug 2016] | 584 (13.2%) | 18,109 (18.7%) | 21,264 (24.7%) | 25,611 (15.3%) | 24,335 (11.4%) | 206 (0.5%) | 0 (0.0%) | 90,109 (14.8%) |
|  | Asthma annual review [July 14- Aug 2016] | 1,038 (23.5%) | 31,231 (32.2%) | 36,004 (41.9%) | 46,229 (27.7%) | 46,553 (21.8%) | 796 (2.1%) | 0 (0.0%) | 161,851 (26.7%) |
|  | Eosinophil count | 3,961 (89.7%) | 70,662 (72.9%) | 59,422 (69.1%) | 94,821 (56.7%) | 126,509 (59.2%) | 17,294 (44.8%) | 282 (57.1%) | 372,951 (61.4%) |
| **Comorbidities** | COPD | 945 (21.4%) | 17,672 (18.2%) | 8,347 (9.7%) | 2,739 (1.6%) | 11,259 (5.3%) | 410 (1.1%) | 2 (0.4%) | 41,374 (6.8%) |
|  | Hayfever | 518 (11.7%) | 14,815 (15.3%) | 14,341 (16.7%) | 28,675 (17.2%) | 38,588 (18.1%) | 4,899 (12.7%) | 45 (9.1%) | 101,881 (16.8%) |
|  | Eczema | 540 (12.2%) | 11,415 (11.8%) | 11,236 (13.1%) | 21,595 (12.9%) | 30,201 (14.1%) | 3,648 (9.5%) | 36 (7.3%) | 78,671 (13.0%) |
|  | Rhinosinusitis | 30 (0.7%) | 542 (0.6%) | 452 (0.5%) | 422 (0.3%) | 556 (0.3%) | 42 (0.1%) | 0 (0.0%) | 2,044 (0.3%) |

Abbreviations: CI, confidence interval; COPD, chronic obstructive pulmonary disease; SD, standard deviation

Supplementary Table 4: Definition of BTS/SIGN steps in children and adults

| **BTS classification** | **Treatment options** |
| --- | --- |
| **Children** | |
| Step 5 | Long-term/frequent use of oral corticosteroid |
| Step 4 | High dose ICS (≥400 µg/day of beclomesthasone or equivalent) |
| Step 3 | Low-medium dose ICS (<400 µg/day beclomethasone or equivalent) plus LABA, or  Low-medium dose ICS (<400 µg/day beclomethasone or equivalent) plus LTRA or theophylline |
| Step 2 | Low-medium dose ICS (<400 µg/day of beclomethasone or equivalent) only (no use of LABA, LTRA or theophylline) |
| Step 1 | SABA as required |
| **Adults** | |
| Step 5 | Long-term/frequent use of oral corticosteroid |
| Step 4 | High dose ICS (≥800 µg/day of beclomethasone or equivalent) plus 1 or more of: LABA, LAMA, LTRA theophylline, chromones |
| Step 3 | Low-medium dose ICS (<800 µg/day of beclomethasone or equivalent) plus LABA (ICS/LABA fixed- or free-dose combination), or  Low-medium dose ICS (<800 µg/day of beclomethasone or equivalent) plus LTRA or theophylline |
| Step 2 | Low-medium dose ICS (<800 µg/day of beclomethasone or equivalent) only (no use of LABA, LAMA, LTRA, theophylline, chromones) |
| Step 1 | SABA only |

Abbreviations: ICS, inhaled corticosteroids; LABA, long-acting beta-2 agonist; LAMA, long-acting muscarinic antagonist; LTRA, leukotriene receptor antagonist; SABA, short-acting beta-2 agonist

Supplementary Table 5: Distribution of paediatric patients in treatment steps, at each 6-month time interval

|  | **All patients** | | | | | | | | **Treated patients only** | | | | |
| --- | --- | --- | --- | --- | --- | --- | --- | --- | --- | --- | --- | --- | --- |
| **Interval** | **N** | **Step 5** | **Step 4** | **Step 3** | **Step 2** | **Step 1** | **Step 0** | **Undiagnosed** | **Step 5** | **Step 4** | **Step 3** | **Step 2** | **Step 1** |
| 1 – December 2007 | 14,545 | 0.3% | 2.5% | 1.3% | 10.3% | 20.5% | 3.2% | 61.9% | 0.9% | 7.2% | 3.7% | 29.5% | 58.7% |
| 2 – June 2008 | 17,093 | 0.3% | 2.8% | 1.5% | 12.4% | 23.5% | 3.7% | 55.8% | 0.7% | 6.9% | 3.7% | 30.6% | 58.0% |
| 3 – December 2008 | 19,754 | 0.3% | 3.3% | 1.7% | 15.0% | 25.5% | 3.9% | 50.3% | 0.7% | 7.2% | 3.7% | 32.8% | 55.7% |
| 4 – June 2009 | 22,225 | 0.3% | 3.9% | 1.9% | 16.9% | 27.3% | 4.4% | 45.3% | 0.6% | 7.8% | 3.8% | 33.6% | 54.3% |
| 5 – December 2009 | 24,829 | 0.4% | 4.4% | 2.2% | 19.3% | 29.0% | 4.4% | 40.4% | 0.7% | 8.0% | 4.0% | 34.9% | 52.4% |
| 6 – June 2010 | 27,206 | 0.3% | 5.1% | 2.4% | 21.6% | 30.7% | 4.6% | 35.2% | 0.5% | 8.5% | 4.0% | 35.9% | 51.1% |
| 7 – December 2010 | 29,568 | 0.5% | 5.6% | 2.6% | 23.5% | 31.2% | 4.6% | 32.1% | 0.8% | 8.8% | 4.1% | 37.1% | 49.2% |
| 8 – June 2011 | 31,631 | 0.4% | 6.5% | 3.0% | 25.7% | 31.8% | 4.8% | 27.9% | 0.6% | 9.6% | 4.5% | 38.1% | 47.2% |
| 9 – December 2011 | 33,786 | 0.4% | 6.9% | 3.2% | 27.4% | 32.4% | 4.7% | 24.9% | 0.6% | 9.8% | 4.6% | 39.0% | 46.1% |
| 10 – June 2012 | 35,146 | 0.4% | 7.9% | 3.6% | 29.7% | 33.7% | 5.0% | 19.7% | 0.5% | 10.5% | 4.8% | 39.4% | 44.8% |
| 11 – December 2012 | 36,279 | 0.4% | 8.4% | 4.0% | 32.7% | 34.5% | 5.1% | 15.0% | 0.5% | 10.5% | 5.0% | 40.9% | 43.1% |
| 12 – June 2013 | 37,167 | 0.3% | 8.9% | 4.4% | 34.9% | 34.8% | 5.2% | 11.6% | 0.4% | 10.7% | 5.3% | 41.9% | 41.8% |
| 13 – December 2013 | 38,102 | 0.3% | 8.9% | 4.5% | 36.6% | 35.3% | 5.2% | 9.1% | 0.4% | 10.4% | 5.3% | 42.8% | 41.2% |
| 14 – June 2014 | 38,842 | 0.2% | 9.4% | 4.9% | 38.2% | 35.5% | 5.2% | 6.5% | 0.2% | 10.7% | 5.6% | 43.3% | 40.2% |
| 15 – December 2014 | 39,510 | 0.3% | 9.9% | 5.1% | 39.5% | 35.9% | 5.2% | 4.2% | 0.3% | 10.9% | 5.6% | 43.6% | 39.6% |
| 16 – June 2015 | 40,022 | 0.2% | 10.4% | 5.3% | 40.4% | 36.1% | 5.2% | 2.4% | 0.2% | 11.3% | 5.7% | 43.7% | 39.1% |
| 17 – December 2015 | 40,077 | 0.3% | 10.6% | 5.4% | 41.2% | 36.4% | 5.2% | 1.0% | 0.3% | 11.3% | 5.8% | 43.9% | 38.8% |
| 18 – June 2016 | 40,096 | 0.2% | 10.8% | 5.1% | 41.6% | 36.9% | 5.2% | 0.2% | 0.2% | 11.4% | 5.4% | 44.0% | 39.0% |

Supplementary Table 6: Distribution of adult patients in treatment steps, at each 6-month time interval

|  | **All patients** | | | | | | | | **Treated patients only** | | | | |
| --- | --- | --- | --- | --- | --- | --- | --- | --- | --- | --- | --- | --- | --- |
| **Interval** | **N** | **Step 5** | **Step 4** | **Step 3** | **Step 2** | **Step 1** | **Step 0** | **Undiagnosed** | **Step 5** | **Step 4** | **Step 3** | **Step 2** | **Step 1** |
| 1 – December 2007 | 455,183 | 0.8% | 13.4% | 7.7% | 21.4% | 24.0% | 12.0% | 20.6% | 1.2% | 19.9% | 11.4% | 31.8% | 35.7% |
| 2 – June 2008 | 467,748 | 0.8% | 13.9% | 8.3% | 22.4% | 26.1% | 10.4% | 18.1% | 1.1% | 19.4% | 11.6% | 31.3% | 36.5% |
| 3 – December 2008 | 481,335 | 0.9% | 14.6% | 8.7% | 23.6% | 27.2% | 9.2% | 15.9% | 1.2% | 19.5% | 11.6% | 31.5% | 36.3% |
| 4 – June 2009 | 492,780 | 0.9% | 15.0% | 9.1% | 24.2% | 28.3% | 8.6% | 14.0% | 1.2% | 19.4% | 11.7% | 31.2% | 36.5% |
| 5 – December 2009 | 505,793 | 1.0% | 15.4% | 9.5% | 24.9% | 28.9% | 8.0% | 12.3% | 1.3% | 19.3% | 11.9% | 31.2% | 36.3% |
| 6 – June 2010 | 516,193 | 0.9% | 15.7% | 9.8% | 25.3% | 29.7% | 7.7% | 10.9% | 1.1% | 19.3% | 12.0% | 31.1% | 36.5% |
| 7 – December 2010 | 527,978 | 1.0% | 15.9% | 10.2% | 25.7% | 30.1% | 7.4% | 9.6% | 1.2% | 19.2% | 12.3% | 31.0% | 36.3% |
| 8 – June 2011 | 537,746 | 1.0% | 16.2% | 10.4% | 25.9% | 30.9% | 7.2% | 8.4% | 1.2% | 19.2% | 12.3% | 30.7% | 36.6% |
| 9 – December 2011 | 549,204 | 1.1% | 16.3% | 10.7% | 26.2% | 31.2% | 7.1% | 7.5% | 1.3% | 19.1% | 12.5% | 30.6% | 36.5% |
| 10 – June 2012 | 558,620 | 1.1% | 16.5% | 10.9% | 26.2% | 31.9% | 6.9% | 6.5% | 1.3% | 19.1% | 12.6% | 30.3% | 36.8% |
| 11 – December 2012 | 569,356 | 1.1% | 16.5% | 11.4% | 26.6% | 32.2% | 6.8% | 5.4% | 1.3% | 18.8% | 13.0% | 30.3% | 36.7% |
| 12 – June 2013 | 577,612 | 1.1% | 16.6% | 11.8% | 26.7% | 32.7% | 6.7% | 4.4% | 1.2% | 18.7% | 13.3% | 30.0% | 36.8% |
| 13 – December 2013 | 586,915 | 1.1% | 16.4% | 12.4% | 27.0% | 33.0% | 6.7% | 3.5% | 1.2% | 18.2% | 13.8% | 30.0% | 36.7% |
| 14 – June 2014 | 594,039 | 1.1% | 16.4% | 12.8% | 27.1% | 33.4% | 6.6% | 2.6% | 1.2% | 18.1% | 14.1% | 29.8% | 36.8% |
| 15 – December 2014 | 601,362 | 1.1% | 16.4% | 13.1% | 27.4% | 33.6% | 6.5% | 1.8% | 1.2% | 17.9% | 14.3% | 29.9% | 36.7% |
| 16 – June 2015 | 606,547 | 1.1% | 16.4% | 13.6% | 27.4% | 34.0% | 6.4% | 1.1% | 1.2% | 17.7% | 14.7% | 29.6% | 36.8% |
| 17 – December 2015 | 606,989 | 1.1% | 16.2% | 14.0% | 27.6% | 34.3% | 6.4% | 0.4% | 1.2% | 17.4% | 15.0% | 29.6% | 36.8% |
| 18 – June 2016 | 607,212 | 0.7% | 16.0% | 14.2% | 27.5% | 35.2% | 6.4% | 0.1% | 0.7% | 17.1% | 15.2% | 29.4% | 37.6% |

Supplementary Table 7: Distribution of paediatric patients by step at each time interval (3-month interval)

|  | **All patients** | | | | | | | |
| --- | --- | --- | --- | --- | --- | --- | --- | --- |
| **Interval** | **N** | **Step 5** | **Step 4** | **Step 3** | **Step 2** | **Step 1** | **Step 0** | **Undiagnosed** |
| 1 - [Jun-07; Sep-07] | 13,129 | 0.2% | 2.0% | 1.1% | 8.3% | 17.9% | 3.2% | 67.2% |
| 2 - [Sep-07; Dec-07] | 14,557 | 0.3% | 2.5% | 1.3% | 10.3% | 20.5% | 3.2% | 62.0% |
| 3 - [Dec-07; Mar-08] | 15,892 | 0.3% | 2.6% | 1.4% | 11.4% | 21.9% | 3.7% | 58.7% |
| 4 - [Mar-08; Jun-08] | 17,109 | 0.3% | 2.8% | 1.5% | 12.4% | 23.4% | 3.7% | 55.8% |
| 5 - [Jun-08; Sep-08] | 18,393 | 0.3% | 3.0% | 1.5% | 13.0% | 23.7% | 3.8% | 54.7% |
| 6 - [Sep-08; Dec-08] | 19,774 | 0.3% | 3.3% | 1.7% | 15.0% | 25.4% | 3.9% | 50.3% |
| 7 - [Dec-08; Mar-09] | 21,018 | 0.3% | 3.6% | 1.7% | 16.1% | 26.7% | 4.3% | 47.2% |
| 8 - [Mar-09; Jun-09] | 22,238 | 0.3% | 3.9% | 1.9% | 16.9% | 27.3% | 4.4% | 45.3% |
| 9 - [Jun-09; Sep-09] | 23,483 | 0.3% | 4.0% | 1.9% | 17.3% | 27.4% | 4.4% | 44.7% |
| 10 - [Sep-09; Dec-09] | 24,848 | 0.4% | 4.4% | 2.1% | 19.2% | 29.0% | 4.4% | 40.5% |
| 11 - [Dec-09; Mar-10] | 26,069 | 0.4% | 4.7% | 2.3% | 20.4% | 30.2% | 4.6% | 37.5% |
| 12 - [Mar-10; Jun-10] | 27,223 | 0.3% | 5.1% | 2.4% | 21.6% | 30.7% | 4.6% | 35.2% |
| 13 - [Jun-10; Sep-10] | 28,339 | 0.3% | 5.3% | 2.4% | 22.0% | 30.6% | 4.6% | 34.8% |
| 14 - [Sep-10; Dec-10] | 29,582 | 0.5% | 5.6% | 2.6% | 23.4% | 31.2% | 4.6% | 32.1% |
| 15 - [Dec-10; Mar-11] | 30,613 | 0.4% | 6.1% | 2.7% | 24.7% | 31.8% | 4.7% | 29.5% |
| 16 - [Mar-11; Jun-11] | 31,647 | 0.4% | 6.5% | 3.0% | 25.7% | 31.8% | 4.8% | 27.9% |
| 17 - [Jun-11; Sep-11] | 32,676 | 0.4% | 6.5% | 3.0% | 26.1% | 31.9% | 4.8% | 27.4% |
| 18 - [Sep-11; Dec-11] | 33,796 | 0.4% | 6.9% | 3.2% | 27.4% | 32.4% | 4.7% | 24.9% |
| 19 - [Dec-11; Mar-12] | 34,601 | 0.5% | 7.5% | 3.3% | 28.6% | 33.1% | 4.8% | 22.2% |
| 20 - [Mar-12; Jun-12] | 35,150 | 0.4% | 7.9% | 3.6% | 29.7% | 33.7% | 5.0% | 19.7% |
| 21 - [Jun-12; Sep-12] | 35,706 | 0.3% | 8.0% | 3.8% | 30.8% | 34.2% | 5.0% | 17.7% |
| 22 - [Sep-12; Dec-12] | 36,282 | 0.4% | 8.4% | 4.0% | 32.7% | 34.5% | 5.1% | 15.0% |
| 23 - [Dec-12; Mar-13] | 36,751 | 0.3% | 8.6% | 4.2% | 34.1% | 34.5% | 5.2% | 13.0% |
| 24 - [Mar-13; Jun-13] | 37,171 | 0.3% | 8.9% | 4.4% | 34.8% | 34.8% | 5.2% | 11.6% |
| 25 - [Jun-13; Sep-13] | 37,610 | 0.3% | 8.7% | 4.3% | 35.3% | 35.3% | 5.2% | 10.9% |
| 26 - [Sep-13; Dec-13] | 38,106 | 0.3% | 8.9% | 4.5% | 36.6% | 35.3% | 5.2% | 9.1% |
| 27 - [Dec-13; Mar-14] | 38,472 | 0.3% | 9.1% | 4.7% | 37.5% | 35.3% | 5.2% | 7.8% |
| 28 - [Mar-14; Jun-14] | 38,848 | 0.2% | 9.4% | 4.9% | 38.2% | 35.5% | 5.2% | 6.5% |
| 29 - [Jun-14; Sep-14] | 39,188 | 0.2% | 9.6% | 4.9% | 38.6% | 35.8% | 5.2% | 5.7% |
| 30 - [Sep-14; Dec-14] | 39,511 | 0.3% | 9.9% | 5.1% | 39.5% | 35.9% | 5.2% | 4.2% |
| 31 - [Dec-14; Mar-15] | 39,791 | 0.3% | 10.2% | 5.2% | 40.1% | 35.9% | 5.2% | 3.1% |
| 32 - [Mar-15; Jun-15] | 40,024 | 0.2% | 10.4% | 5.3% | 40.4% | 36.1% | 5.2% | 2.4% |
| 33 - [Jun-15; Sep-15] | 40,054 | 0.2% | 10.4% | 5.2% | 40.8% | 36.4% | 5.2% | 1.7% |
| 34 - [Sep-15; Dec-15] | 40,077 | 0.3% | 10.6% | 5.4% | 41.2% | 36.4% | 5.2% | 1.0% |
| 35 - [Dec-15; Mar-16] | 40,089 | 0.3% | 10.8% | 5.3% | 41.4% | 36.5% | 5.2% | 0.5% |
| 36 - [Mar-16; Jun-16] | 40,096 | 0.2% | 10.8% | 5.1% | 41.6% | 36.9% | 5.2% | 0.2% |

Supplementary Table 8: Distribution of adult patients by step at each time interval (3-month interval)

| **Interval** | **N** | **Step 5** | **Step 4** | **Step 3** | **Step 2** | **Step 1** | **Step 0** | **Un-diagnosed** |
| --- | --- | --- | --- | --- | --- | --- | --- | --- |
| 1 - [Jun-07; Sep-07] | 447,374 | 0.8% | 13.0% | 7.4% | 20.2% | 22.8% | 13.5% | 22.4% |
| 2 - [Sep-07; Dec-07] | 455,266 | 0.8% | 13.4% | 7.7% | 21.4% | 24.0% | 12.0% | 20.7% |
| 3 - [Dec-07; Mar-08] | 461,800 | 0.8% | 13.7% | 8.0% | 21.9% | 25.2% | 11.1% | 19.2% |
| 4 - [Mar-08; Jun-08] | 467,815 | 0.8% | 13.9% | 8.3% | 22.4% | 26.1% | 10.4% | 18.1% |
| 5 - [Jun-08; Sep-08] | 474,509 | 0.9% | 14.2% | 8.4% | 22.9% | 26.8% | 9.7% | 17.1% |
| 6 - [Sep-08; Dec-08] | 481,397 | 0.9% | 14.5% | 8.7% | 23.6% | 27.2% | 9.2% | 15.9% |
| 7 - [Dec-08; Mar-09] | 487,215 | 0.9% | 14.8% | 8.8% | 23.9% | 27.8% | 8.9% | 14.9% |
| 8 - [Mar-09; Jun-09] | 492,833 | 0.9% | 15.0% | 9.1% | 24.2% | 28.3% | 8.6% | 14.0% |
| 9 - [Jun-09; Sep-09] | 499,233 | 0.9% | 15.2% | 9.2% | 24.4% | 28.7% | 8.3% | 13.3% |
| 10 - [Sep-09; Dec-09] | 505,846 | 1.0% | 15.4% | 9.5% | 24.9% | 28.9% | 8.0% | 12.3% |
| 11 - [Dec-09; Mar-10] | 511,215 | 0.9% | 15.6% | 9.6% | 25.1% | 29.3% | 7.8% | 11.6% |
| 12 - [Mar-10; Jun-10] | 516,236 | 0.9% | 15.7% | 9.8% | 25.3% | 29.7% | 7.7% | 10.9% |
| 13 - [Jun-10; Sep-10] | 521,881 | 0.9% | 15.8% | 9.9% | 25.4% | 30.1% | 7.6% | 10.4% |
| 14 - [Sep-10; Dec-10] | 528,028 | 1.0% | 15.9% | 10.1% | 25.7% | 30.1% | 7.4% | 9.6% |
| 15 - [Dec-10; Mar-11] | 533,057 | 1.0% | 16.1% | 10.3% | 25.8% | 30.6% | 7.3% | 8.9% |
| 16 - [Mar-11; Jun-11] | 537,793 | 1.0% | 16.2% | 10.4% | 25.9% | 30.9% | 7.2% | 8.4% |
| 17 - [Jun-11; Sep-11] | 543,307 | 1.0% | 16.2% | 10.5% | 25.9% | 31.2% | 7.2% | 8.1% |
| 18 - [Sep-11; Dec-11] | 549,258 | 1.1% | 16.3% | 10.7% | 26.2% | 31.2% | 7.1% | 7.5% |
| 19 - [Dec-11; Mar-12] | 553,922 | 1.1% | 16.4% | 10.8% | 26.3% | 31.6% | 7.0% | 6.9% |
| 20 - [Mar-12; Jun-12] | 558,684 | 1.1% | 16.5% | 10.9% | 26.2% | 31.9% | 6.9% | 6.5% |
| 21 - [Jun-12; Sep-12] | 563,852 | 1.1% | 16.5% | 11.1% | 26.3% | 32.1% | 6.9% | 6.0% |
| 22 - [Sep-12; Dec-12] | 569,418 | 1.1% | 16.5% | 11.4% | 26.6% | 32.2% | 6.8% | 5.4% |
| 23 - [Dec-12; Mar-13] | 573,608 | 1.1% | 16.5% | 11.6% | 26.7% | 32.5% | 6.8% | 4.8% |
| 24 - [Mar-13; Jun-13] | 577,649 | 1.1% | 16.6% | 11.8% | 26.7% | 32.7% | 6.7% | 4.4% |
| 25 - [Jun-13; Sep-13] | 582,154 | 1.1% | 16.4% | 12.0% | 26.7% | 33.1% | 6.7% | 4.0% |
| 26 - [Sep-13; Dec-13] | 586,971 | 1.1% | 16.4% | 12.4% | 27.0% | 33.0% | 6.7% | 3.5% |
| 27 - [Dec-13; Mar-14] | 590,493 | 1.1% | 16.4% | 12.6% | 27.1% | 33.2% | 6.6% | 3.0% |
| 28 - [Mar-14; Jun-14] | 594,085 | 1.1% | 16.4% | 12.8% | 27.1% | 33.4% | 6.6% | 2.6% |
| 29 - [Jun-14; Sep-14] | 597,761 | 1.1% | 16.4% | 13.0% | 27.2% | 33.6% | 6.5% | 2.3% |
| 30 - [Sep-14; Dec-14] | 601,412 | 1.1% | 16.4% | 13.1% | 27.4% | 33.6% | 6.5% | 1.8% |
| 31 - [Dec-14; Mar-15] | 604,165 | 1.1% | 16.4% | 13.4% | 27.4% | 33.9% | 6.5% | 1.4% |
| 32 - [Mar-15; Jun-15] | 606,583 | 1.1% | 16.4% | 13.6% | 27.4% | 34.0% | 6.4% | 1.1% |
| 33 - [Jun-15; Sep-15] | 606,850 | 1.0% | 16.3% | 13.8% | 27.5% | 34.3% | 6.4% | 0.7% |
| 34 - [Sep-15; Dec-15] | 606,989 | 1.1% | 16.2% | 14.0% | 27.6% | 34.3% | 6.4% | 0.4% |
| 35 - [Dec-15; Mar-16] | 607,106 | 1.0% | 16.2% | 14.2% | 27.6% | 34.4% | 6.4% | 0.2% |
| 36 - [Mar-16; Jun-16] | 607,212 | 0.7% | 16.0% | 14.2% | 27.5% | 35.2% | 6.4% | 0.1% |

Supplementary Table 9: Proportion of paediatric patients stepping up, down or remaining stable within a 3-month interval (patients in steps 0-5)

| **Interval** | **N** | **Up** | **Down** | **Stable** |
| --- | --- | --- | --- | --- |
| 1- [Jun-07; Sep-07] | 3,392 | 6.0% | 3.5% | 90.5% |
| 2- [Sep-07; Dec-07] | 3,881 | 9.7% | 5.2% | 85.1% |
| 3- [Dec-07; Mar-08] | 5,065 | 7.8% | 4.5% | 87.7% |
| 4- [Mar-08; Jun-08] | 5,979 | 6.3% | 3.9% | 89.8% |
| 5- [Jun-08; Sep-08] | 6,925 | 5.5% | 3.6% | 90.9% |
| 6- [Sep-08; Dec-08] | 7,628 | 9.9% | 4.9% | 85.1% |
| 7- [Dec-08; Mar-09] | 9,049 | 7.1% | 4.3% | 88.6% |
| 8- [Mar-09; Jun-09] | 10,183 | 5.9% | 3.8% | 90.3% |
| 9- [Jun-09; Sep-09] | 11,179 | 4.9% | 3.3% | 91.8% |
| 10- [Sep-09; Dec-09] | 11,952 | 9.2% | 5.0% | 85.8% |
| 11- [Dec-09; Mar-10] | 13,689 | 7.3% | 4.6% | 88.1% |
| 12- [Mar-10; Jun-10] | 15,111 | 6.9% | 3.9% | 89.2% |
| 13- [Jun-10; Sep-10] | 16,382 | 4.4% | 3.1% | 92.4% |
| 14- [Sep-10; Dec-10] | 17,163 | 8.0% | 4.7% | 87.3% |
| 15- [Dec-10; Mar-11] | 18,723 | 6.8% | 4.2% | 89.1% |
| 16- [Mar-11; Jun-11] | 20,132 | 6.1% | 3.4% | 90.5% |
| 17- [Jun-11; Sep-11] | 21,300 | 4.4% | 3.4% | 92.2% |
| 18- [Sep-11; Dec-11] | 22,155 | 7.4% | 4.6% | 88.1% |
| 19- [Dec-11; Mar-12] | 23,765 | 6.5% | 4.0% | 89.5% |
| 20- [Mar-12; Jun-12] | 25,249 | 5.5% | 3.7% | 90.8% |
| 21- [Jun-12; Sep-12] | 26,474 | 5.1% | 3.5% | 91.4% |
| 22- [Sep-12; Dec-12] | 27,578 | 6.9% | 4.5% | 88.6% |
| 23- [Dec-12; Mar-13] | 29,004 | 5.6% | 3.7% | 90.7% |
| 24- [Mar-13; Jun-13] | 30,072 | 4.6% | 3.5% | 91.9% |
| 25- [Jun-13; Sep-13] | 30,918 | 3.4% | 3.6% | 93.0% |
| 26- [Sep-13; Dec-13] | 31,555 | 5.6% | 4.1% | 90.3% |
| 27- [Dec-13; Mar-14] | 32,627 | 4.8% | 3.5% | 91.7% |
| 28- [Mar-14; Jun-14] | 33,465 | 4.3% | 3.4% | 92.3% |
| 29- [Jun-14; Sep-14] | 34,299 | 3.5% | 3.0% | 93.5% |
| 30- [Sep-14; Dec-14] | 34,919 | 4.9% | 3.7% | 91.4% |
| 31- [Dec-14; Mar-15] | 35,799 | 4.2% | 3.2% | 92.6% |
| 32- [Mar-15; Jun-15] | 36,474 | 3.4% | 2.9% | 93.7% |
| 33- [Jun-15; Sep-15] | 36,982 | 2.7% | 2.5% | 94.8% |
| 34- [Sep-15; Dec-15] | 37,291 | 3.6% | 2.9% | 93.4% |
| 35- [Dec-15; Mar-16] | 37,584 | 3.0% | 2.7% | 94.3% |
| 36- [Mar-16; Jun-16] | 37,795 | 2.2% | 2.4% | 95.4% |

Supplementary Table 10: Proportion of adult patients stepping up, down or remaining stable within a 3-month interval (patients in steps 0-5)

| **Interval** | **N** | **Up** | **Down** | **Stable** |
| --- | --- | --- | --- | --- |
| 1 - [Jun-07 ; Sep-07] | 265,546 | 5.9% | 5.1% | 89.0% |
| 2 - [Sep-07 ; Dec-07] | 286,857 | 6.5% | 5.0% | 88.4% |
| 3 - [Dec-07 ; Mar-08] | 306,667 | 5.5% | 4.8% | 89.7% |
| 4 - [Mar-08 ; Jun-08] | 321,635 | 5.2% | 4.5% | 90.3% |
| 5 - [Jun-08 ; Sep-08] | 334,489 | 5.1% | 4.4% | 90.5% |
| 6 - [Sep-08 ; Dec-08] | 347,271 | 5.9% | 4.4% | 89.7% |
| 7 - [Dec-08 ; Mar-09] | 360,761 | 4.7% | 4.3% | 91.0% |
| 8 - [Mar-09 ; Jun-09] | 371,422 | 4.6% | 4.0% | 91.4% |
| 9 - [Jun-09 ; Sep-09] | 381,405 | 4.5% | 3.9% | 91.6% |
| 10 - [Sep-09 ; Dec-09] | 391,440 | 5.1% | 4.0% | 90.9% |
| 11 - [Dec-09 ; Mar-10] | 402,917 | 4.3% | 3.8% | 91.9% |
| 12 - [Mar-10 ; Jun-10] | 411,877 | 4.1% | 3.7% | 92.1% |
| 13 - [Jun-10 ; Sep-10] | 420,385 | 3.6% | 3.4% | 92.9% |
| 14 - [Sep-10 ; Dec-10] | 428,395 | 4.4% | 3.5% | 92.1% |
| 15 - [Dec-10 ; Mar-11] | 437,947 | 3.9% | 3.6% | 92.5% |
| 16 - [Mar-11 ; Jun-11] | 446,401 | 3.5% | 3.3% | 93.2% |
| 17 - [Jun-11 ; Sep-11] | 453,538 | 3.3% | 3.1% | 93.6% |
| 18 - [Sep-11 ; Dec-11] | 460,606 | 3.8% | 3.1% | 93.1% |
| 19 - [Dec-11 ; Mar-12] | 469,237 | 3.4% | 3.2% | 93.4% |
| 20 - [Mar-12 ; Jun-12] | 476,838 | 3.2% | 3.0% | 93.8% |
| 21 - [Jun-12 ; Sep-12] | 483,828 | 3.3% | 3.1% | 93.5% |
| 22 - [Sep-12 ; Dec-12] | 491,644 | 3.6% | 3.1% | 93.4% |
| 23 - [Dec-12 ; Mar-13] | 499,998 | 3.1% | 3.0% | 93.9% |
| 24 - [Mar-13 ; Jun-13] | 507,177 | 2.9% | 2.8% | 94.3% |
| 25 - [Jun-13 ; Sep-13] | 513,369 | 2.7% | 2.8% | 94.5% |
| 26 - [Sep-13 ; Dec-13] | 519,874 | 3.2% | 2.6% | 94.2% |
| 27 - [Dec-13 ; Mar-14] | 527,106 | 2.7% | 2.6% | 94.7% |
| 28 - [Mar-14 ; Jun-14] | 533,488 | 2.6% | 2.4% | 95.0% |
| 29 - [Jun-14 ; Sep-14] | 539,439 | 2.3% | 2.3% | 95.4% |
| 30 - [Sep-14 ; Dec-14] | 545,212 | 2.5% | 2.2% | 95.3% |
| 31 - [Dec-14 ; Mar-15] | 551,275 | 2.3% | 2.3% | 95.4% |
| 32 - [Mar-15 ; Jun-15] | 556,762 | 2.1% | 2.1% | 95.9% |
| 33 - [Jun-15 ; Sep-15] | 561,106 | 1.8% | 1.9% | 96.2% |
| 34 - [Sep-15 ; Dec-15] | 563,576 | 2.0% | 1.8% | 96.2% |
| 35 - [Dec-15 ; Mar-16] | 565,476 | 1.7% | 1.7% | 96.6% |
| 36 - [Mar-16 ; Jun-16] | 567,029 | 1.3% | 2.3% | 96.4% |

Supplementary Table 11: Transitions between treatment steps from one interval (T-1) to the next (T), by treatment in paediatric patients (3-month interval)

|  | | **TO: (Time interval T)** | | | | | |
| --- | --- | --- | --- | --- | --- | --- | --- |
|  |  | **Step 5** | **Step 4** | **Step 3** | **Step 2** | **Step 1** | **Step 0** |
| **FROM: (Time interval T-1)** | **Step 5** | 74.2% | 5.5% | 3.0% | 7.3% | 10.0% | 0.0% |
|  | **Step 4** | 0.2% | 92.9% | 0.6% | 1.4% | 4.9% | 0.0% |
|  | **Step 3** | 0.2% | 1.5% | 87.2% | 6.0% | 5.2% | 0.0% |
|  | **Step 2** | 0.1% | 0.7% | 1.0% | 93.0% | 5.3% | 0.0% |
|  | **Step 1** | 0.1% | 1.4% | 0.7% | 7.2% | 90.6% | 0.0% |
|  | **Step 0** | 0.0% | 0.1% | 0.0% | 0.6% | 1.0% | 98.2% |

Supplementary Table 12: Transitions between treatment steps from one interval (T-1) to the next (T), by treatment in adult patients (3-month interval)

|  | | **TO: (Time interval T)** | | | | | |
| --- | --- | --- | --- | --- | --- | --- | --- |
|  |  | **Step 5** | **Step 4** | **Step 3** | **Step 2** | **Step 1** | **Step 0** |
| **FROM: (Time interval T-1)** | **Step 5** | 85.4% | 7.7% | 2.5% | 1.2% | 3.2% | 0.0% |
|  | **Step 4** | 0.5% | 94.7% | 1.0% | 0.4% | 3.4% | 0.0% |
|  | **Step 3** | 0.2% | 1.2% | 94.0% | 1.1% | 3.5% | 0.0% |
|  | **Step 2** | 0.1% | 0.5% | 0.8% | 94.0% | 4.6% | 0.0% |
|  | **Step 1** | 0.1% | 1.8% | 1.4% | 4.2% | 92.6% | 0.0% |
|  | **Step 0** | 0.0% | 0.2% | 0.2% | 1.2% | 2.3% | 96.1% |

Supplementary Table 13: Distribution of paediatric patients by step at each time interval (grace period of 30 days)

| **Interval** | **N** | **Step 5** | **Step 4** | **Step 3** | **Step 2** | **Step 1** | **Step 0** | **Undiagnosed** |
| --- | --- | --- | --- | --- | --- | --- | --- | --- |
| 1- [Dec-07; Jun-07] | 14,545 | 0.2% | 2.5% | 1.2% | 10.3% | 20.7% | 3.2% | 61.9% |
| 2- [Jun-08; Dec-07] | 17,093 | 0.2% | 2.8% | 1.4% | 12.7% | 23.4% | 3.7% | 55.8% |
| 3- [Dec-08; Jun-08] | 19,754 | 0.2% | 3.3% | 1.4% | 15.3% | 25.6% | 3.9% | 50.3% |
| 4- [Jun-09; Dec-08] | 22,225 | 0.2% | 3.9% | 1.6% | 17.3% | 27.3% | 4.4% | 45.3% |
| 5- [Dec-09; Jun-09] | 24,829 | 0.3% | 4.3% | 1.8% | 19.5% | 29.1% | 4.4% | 40.4% |
| 6- [Jun-10; Dec-09] | 27,206 | 0.3% | 5.1% | 2.1% | 22.1% | 30.5% | 4.6% | 35.2% |
| 7- [Dec-10; Jun-10] | 29,568 | 0.3% | 5.6% | 2.2% | 23.9% | 31.3% | 4.6% | 32.1% |
| 8- [Jun-11; Dec-10] | 31,631 | 0.3% | 6.5% | 2.5% | 26.3% | 31.7% | 4.8% | 27.9% |
| 9- [Dec-11; Jun-11] | 33,786 | 0.4% | 6.9% | 2.6% | 27.9% | 32.5% | 4.7% | 24.9% |
| 10- [Jun-12; Dec-11] | 35,146 | 0.3% | 7.8% | 3.0% | 30.6% | 33.5% | 5.0% | 19.7% |
| 11- [Dec-12; Jun-12] | 36,279 | 0.3% | 8.3% | 3.3% | 33.4% | 34.6% | 5.1% | 15.0% |
| 12- [Jun-13; Dec-12] | 37,167 | 0.3% | 8.9% | 3.7% | 35.9% | 34.4% | 5.2% | 11.6% |
| 13- [Dec-13; Jun-13] | 38,102 | 0.3% | 8.9% | 3.8% | 37.5% | 35.1% | 5.2% | 9.1% |
| 14- [Jun-14; Dec-13] | 38,842 | 0.2% | 9.4% | 4.1% | 39.4% | 35.1% | 5.2% | 6.5% |
| 15- [Dec-14; Jun-14] | 39,510 | 0.2% | 9.8% | 4.3% | 40.6% | 35.6% | 5.2% | 4.2% |
| 16- [Jun-15; Dec-14] | 40,022 | 0.2% | 10.3% | 4.6% | 41.7% | 35.6% | 5.2% | 2.4% |
| 17- [Dec-15; Jun-15] | 40,077 | 0.2% | 10.6% | 4.6% | 42.5% | 35.9% | 5.2% | 1.0% |
| 18- [Jun-16; Dec-15] | 40,096 | 0.2% | 10.9% | 4.4% | 43.3% | 35.9% | 5.2% | 0.2% |

Supplementary Table 14: Distribution of adult patients by step at each time interval (grace period of 30 days)

| **Interval** | **N** | **Step 5** | **Step 4** | **Step 3** | **Step 2** | **Step 1** | **Step 0** | **Un-diagnosed** |
| --- | --- | --- | --- | --- | --- | --- | --- | --- |
| 1 - [Dec-07; Jun-07] | 455,183 | 0.5% | 13.0% | 7.1% | 21.7% | 25.1% | 12.0% | 20.6% |
| 2 - [Jun-08; Dec-07] | 467,748 | 0.5% | 13.5% | 7.6% | 22.8% | 27.0% | 10.4% | 18.1% |
| 3 - [Dec-08; Jun-08] | 481,335 | 0.6% | 14.1% | 8.1% | 23.9% | 28.2% | 9.2% | 15.9% |
| 4 - [Jun-09; Dec-08] | 492,780 | 0.6% | 14.6% | 8.5% | 24.6% | 29.2% | 8.6% | 14.0% |
| 5 - [Dec-09; Jun-09] | 505,793 | 0.6% | 15.1% | 8.9% | 25.3% | 29.8% | 8.0% | 12.3% |
| 6 - [Jun-10; Dec-09] | 516,193 | 0.6% | 15.4% | 9.2% | 25.8% | 30.4% | 7.7% | 10.9% |
| 7 - [Dec-10; Jun-10] | 527,978 | 0.7% | 15.6% | 9.6% | 26.2% | 30.9% | 7.4% | 9.6% |
| 8 - [Jun-11; Dec-10] | 537,746 | 0.7% | 15.9% | 9.9% | 26.3% | 31.6% | 7.2% | 8.4% |
| 9 - [Dec-11; Jun-11] | 549,204 | 0.7% | 16.1% | 10.2% | 26.6% | 31.9% | 7.1% | 7.5% |
| 10 - [Jun-12; Dec-11] | 558,620 | 0.8% | 16.2% | 10.5% | 26.7% | 32.5% | 6.9% | 6.5% |
| 11 - [Dec-12; Jun-12] | 569,356 | 0.8% | 16.3% | 10.9% | 27.0% | 32.8% | 6.8% | 5.4% |
| 12 - [Jun-13; Dec-12] | 577,612 | 0.8% | 16.3% | 11.4% | 27.2% | 33.2% | 6.7% | 4.4% |
| 13 - [Dec-13; Jun-13] | 586,915 | 0.8% | 16.2% | 11.9% | 27.4% | 33.5% | 6.7% | 3.5% |
| 14 - [Jun-14; Dec-13] | 594,039 | 0.8% | 16.2% | 12.4% | 27.6% | 33.9% | 6.6% | 2.6% |
| 15 - [Dec-14; Jun-14] | 601,362 | 0.9% | 16.2% | 12.8% | 27.8% | 34.0% | 6.5% | 1.8% |
| 16 - [Jun-15; Dec-14] | 606,547 | 0.8% | 16.2% | 13.2% | 27.9% | 34.3% | 6.4% | 1.1% |
| 17 - [Dec-15; Jun-15] | 606,989 | 0.8% | 16.1% | 13.6% | 28.1% | 34.5% | 6.4% | 0.4% |
| 18 - [Jun-16; Dec-15] | 607,212 | 0.7% | 15.9% | 13.9% | 28.1% | 34.9% | 6.4% | 0.1% |

Supplementary Table 15: Proportion of paediatric patients stepping up, down or remaining stable within a 6-month interval (patients in steps 0-5; grace period of 30 days)

| **Interval** | **N** | **Up** | **Down** | **Stable** |
| --- | --- | --- | --- | --- |
| 1 - [Dec-07; Jun-07] | 3,392 | 14.0% | 7.9% | 78.1% |
| 2 - [Jun-08; Dec-07] | 5,065 | 13.4% | 7.7% | 78.9% |
| 3 - [Dec-08; Jun-08] | 6,925 | 13.4% | 7.6% | 79.0% |
| 4 - [Jun-09; Dec-08] | 9,049 | 12.4% | 7.3% | 80.3% |
| 5 - [Dec-09; Jun-09] | 11,179 | 12.8% | 7.9% | 79.4% |
| 6 - [Jun-10; Dec-09] | 13,689 | 13.3% | 7.5% | 79.2% |
| 7 - [Dec-10; Jun-10] | 16,382 | 11.3% | 7.6% | 81.1% |
| 8 - [Jun-11; Dec-10] | 18,723 | 12.2% | 6.9% | 80.9% |
| 9 - [Dec-11; Jun-11] | 21,300 | 10.7% | 7.4% | 81.9% |
| 10 - [Jun-12; Dec-11] | 23,765 | 11.4% | 7.1% | 81.5% |
| 11 - [Dec-12; Jun-12] | 26,474 | 10.6% | 7.4% | 82.1% |
| 12 - [Jun-13; Dec-12] | 29,004 | 10.1% | 6.7% | 83.3% |
| 13 - [Dec-13; Jun-13] | 30,918 | 8.3% | 7.4% | 84.3% |
| 14 - [Jun-14; Dec-13] | 32,627 | 8.6% | 6.5% | 84.9% |
| 15 - [Dec-14; Jun-14] | 34,299 | 7.8% | 6.3% | 85.9% |
| 16 - [Jun-15; Dec-14] | 35,799 | 7.3% | 5.6% | 87.1% |
| 17 - [Dec-15; Jun-15] | 36,982 | 5.9% | 5.2% | 88.9% |
| 18 - [Jun-16; Dec-15] | 37,584 | 5.2% | 4.7% | 90.1% |

Supplementary Table 16: Transitions between treatment steps from one interval (T-1) to the next (T), by treatment in paediatric patients (grace period of 30 days)

|  | | **TO: (Time interval T)** | | | | | |
| --- | --- | --- | --- | --- | --- | --- | --- |
|  |  | **Step 5** | **Step 4** | **Step 3** | **Step 2** | **Step 1** | **Step 0** |
| **FROM: (Time interval T-1)** | **Step 5** | 63.6% | 5.7% | 2.1% | 12.0% | 16.6% | 0.0% |
|  | **Step 4** | 0.1% | 86.6% | 1.1% | 3.0% | 9.1% | 0.0% |
|  | **Step 3** | 0.1% | 3.0% | 73.1% | 14.3% | 9.4% | 0.0% |
|  | **Step 2** | 0.1% | 1.4% | 1.7% | 87.0% | 9.8% | 0.0% |
|  | **Step 1** | 0.1% | 2.7% | 1.1% | 13.7% | 82.4% | 0.0% |
|  | **Step 0** | 0.0% | 0.2% | 0.1% | 1.2% | 1.8% | 96.7% |

Supplementary Table 17: Proportion of adult patients stepping up, down or remaining stable within a 6-month interval (patients in steps 0-5; grace period of 30 days)

| **Interval** | **N** | **Up** | **Down** | **Stable** |
| --- | --- | --- | --- | --- |
| 1 - [Dec-07; Jun-07] | 265,546 | 11.3% | 9.3% | 79.5% |
| 2 - [Jun-08; Dec-07] | 306,667 | 10.0% | 8.5% | 81.5% |
| 3 - [Dec-08; Jun-08] | 334,489 | 10.0% | 8.1% | 82.0% |
| 4 - [Jun-09; Dec-08] | 360,761 | 8.6% | 7.6% | 83.8% |
| 5 - [Dec-09; Jun-09] | 381,405 | 8.8% | 7.3% | 83.9% |
| 6 - [Jun-10; Dec-09] | 402,917 | 7.8% | 6.9% | 85.3% |
| 7 - [Dec-10; Jun-10] | 420,385 | 7.3% | 6.5% | 86.2% |
| 8 - [Jun-11; Dec-10] | 437,947 | 6.8% | 6.4% | 86.8% |
| 9 - [Dec-11; Jun-11] | 453,538 | 6.6% | 5.8% | 87.6% |
| 10 - [Jun-12; Dec-11] | 469,237 | 6.1% | 5.7% | 88.2% |
| 11 - [Dec-12; Jun-12] | 483,828 | 6.3% | 5.7% | 88.0% |
| 12 - [Jun-13; Dec-12] | 499,998 | 5.7% | 5.3% | 89.1% |
| 13 - [Dec-13; Jun-13] | 513,369 | 5.3% | 5.1% | 89.6% |
| 14 - [Jun-14; Dec-13] | 527,106 | 4.9% | 4.7% | 90.4% |
| 15 - [Dec-14; Jun-14] | 539,439 | 4.6% | 4.1% | 91.3% |
| 16 - [Jun-15; Dec-14] | 551,275 | 4.2% | 4.0% | 91.9% |
| 17 - [Dec-15; Jun-15] | 561,106 | 3.6% | 3.4% | 93.0% |
| 18 - [Jun-16; Dec-15] | 565,476 | 3.0% | 3.3% | 93.7% |

Supplementary Table 18: Transitions between treatment steps from one interval (T-1) to the next (T), by treatment in adult patients (grace period of 30 days)

|  | | **TO: (Time interval T)** | | | | | |
| --- | --- | --- | --- | --- | --- | --- | --- |
|  |  | **Step 5** | **Step 4** | **Step 3** | **Step 2** | **Step 1** | **Step 0** |
| **FROM: (Time interval T-1)** | **Step 5** | 74.5% | 11.3% | 3.9% | 2.8% | 7.5% | 0.0% |
|  | **Step 4** | 0.5% | 89.9% | 2.0% | 0.9% | 6.7% | 0.0% |
|  | **Step 3** | 0.3% | 2.3% | 88.5% | 2.4% | 6.5% | 0.0% |
|  | **Step 2** | 0.1% | 1.0% | 1.6% | 89.1% | 8.2% | 0.0% |
|  | **Step 1** | 0.2% | 3.4% | 2.4% | 7.4% | 86.5% | 0.0% |
|  | **Step 0** | 0.0% | 0.5% | 0.3% | 2.5% | 4.3% | 92.3% |

Supplementary Table 19: Distribution of paediatric patients by step at each time interval (excluding patients with COPD)

| **Interval** | **N** | **Step 5** | **Step 4** | **Step 3** | **Step 2** | **Step 1** | **Step 0** | **Undiagnosed** |
| --- | --- | --- | --- | --- | --- | --- | --- | --- |
| 1 - [Dec-07; Jun-07] | 14,522 | 0.3% | 2.4% | 1.3% | 10.3% | 20.6% | 3.2% | 61.9% |
| 2 - [Jun-08; Dec-07] | 17,066 | 0.3% | 2.8% | 1.5% | 12.4% | 23.5% | 3.7% | 55.8% |
| 3 - [Dec-08; Jun-08] | 19,723 | 0.3% | 3.3% | 1.7% | 15.0% | 25.5% | 3.9% | 50.3% |
| 4 - [Jun-09; Dec-08] | 22,185 | 0.3% | 3.9% | 1.9% | 16.9% | 27.4% | 4.4% | 45.3% |
| 5 - [Dec-09; Jun-09] | 24,781 | 0.4% | 4.3% | 2.1% | 19.3% | 29.0% | 4.4% | 40.4% |
| 6 - [Jun-10; Dec-09] | 27,150 | 0.3% | 5.1% | 2.4% | 21.6% | 30.7% | 4.6% | 35.2% |
| 7 - [Dec-10; Jun-10] | 29,506 | 0.5% | 5.6% | 2.6% | 23.5% | 31.2% | 4.6% | 32.1% |
| 8 - [Jun-11; Dec-10] | 31,562 | 0.4% | 6.4% | 3.0% | 25.8% | 31.8% | 4.7% | 27.9% |
| 9 - [Dec-11; Jun-11] | 33,712 | 0.4% | 6.9% | 3.2% | 27.4% | 32.4% | 4.7% | 24.9% |
| 10 - [Jun-12; Dec-11] | 35,066 | 0.4% | 7.9% | 3.6% | 29.7% | 33.8% | 5.0% | 19.7% |
| 11 - [Dec-12; Jun-12] | 36,193 | 0.4% | 8.3% | 4.0% | 32.7% | 34.5% | 5.1% | 15.0% |
| 12 - [Jun-13; Dec-12] | 37,080 | 0.3% | 8.8% | 4.4% | 34.8% | 34.8% | 5.2% | 11.6% |
| 13 - [Dec-13; Jun-13] | 38,013 | 0.3% | 8.9% | 4.5% | 36.6% | 35.3% | 5.2% | 9.1% |
| 14 - [Jun-14; Dec-13] | 38,749 | 0.2% | 9.4% | 4.9% | 38.2% | 35.6% | 5.2% | 6.5% |
| 15 - [Dec-14; Jun-14] | 39,416 | 0.3% | 9.9% | 5.1% | 39.5% | 35.9% | 5.2% | 4.2% |
| 16 - [Jun-15; Dec-14] | 39,928 | 0.2% | 10.3% | 5.3% | 40.4% | 36.1% | 5.2% | 2.4% |
| 17 - [Dec-15 ; Jun-15] | 39,983 | 0.3% | 10.6% | 5.4% | 41.2% | 36.4% | 5.2% | 1.0% |
| 18 - [Jun-16 ; Dec-15] | 40,002 | 0.2% | 10.7% | 5.1% | 41.6% | 36.9% | 5.2% | 0.2% |

Supplementary Table 20: Proportion of paediatric patients stepping up, down or remaining stable within a 6-month interval (patients in steps 0-5; excluding patients with COPD)

| **Interval** | **N** | **Up** | **Down** | **Stable** |
| --- | --- | --- | --- | --- |
| 1 - [Dec-07; Jun-07] | 3,386 | 13.7% | 7.4% | 78.9% |
| 2 - [Jun-08; Dec-07] | 5,058 | 12.3% | 7.1% | 80.6% |
| 3 - [Dec-08; Jun-08] | 6,915 | 13.4% | 7.0% | 79.5% |
| 4 - [Jun-09; Dec-08] | 9,034 | 11.2% | 6.7% | 82.1% |
| 5 - [Dec-09; Jun-09] | 11,159 | 12.3% | 7.1% | 80.6% |
| 6 - [Jun-10; Dec-09] | 13,662 | 12.1% | 6.8% | 81.1% |
| 7 - [Dec-10; Jun-10] | 16,347 | 11.1% | 6.8% | 82.1% |
| 8 - [Jun-11; Dec-10] | 18,685 | 11.1% | 6.3% | 82.7% |
| 9 - [Dec-11; Jun-11] | 21,257 | 10.2% | 6.7% | 83.1% |
| 10 - [Jun-12; Dec-11] | 23,712 | 10.3% | 6.3% | 83.4% |
| 11 - [Dec-12; Jun-12] | 26,414 | 10.1% | 6.7% | 83.2% |
| 12 - [Jun-13; Dec-12] | 28,936 | 8.7% | 6.0% | 85.2% |
| 13 - [Dec-13; Jun-13] | 30,847 | 7.6% | 6.6% | 85.8% |
| 14 - [Jun-14; Dec-13] | 32,553 | 7.9% | 5.9% | 86.2% |
| 15 - [Dec-14; Jun-14] | 34,217 | 7.3% | 5.7% | 87.0% |
| 16 - [Jun-15; Dec-14] | 35,715 | 6.5% | 5.2% | 88.3% |
| 17 - [Dec-15; Jun-15] | 36,898 | 5.5% | 4.7% | 89.8% |
| 18 - [Jun-16; Dec-15] | 37,500 | 4.5% | 4.5% | 91.0% |

Supplementary Table 21: Transitions between treatment steps from one interval (T-1) to the next (T), by treatment in paediatric patients (excluding patients with COPD)

|  | | **TO: (Time interval T)** | | | | | |
| --- | --- | --- | --- | --- | --- | --- | --- |
|  |  | **Step 5** | **Step 4** | **Step 3** | **Step 2** | **Step 1** | **Step 0** |
| **FROM: (Time interval T-1)** | **Step 5** | 59.0% | 9.0% | 4.9% | 11.5% | 15.5% | 0.0% |
|  | **Step 4** | 0.3% | 87.9% | 1.2% | 2.6% | 8.0% | 0.0% |
|  | **Step 3** | 0.3% | 2.9% | 78.6% | 10.2% | 8.1% | 0.0% |
|  | **Step 2** | 0.1% | 1.3% | 1.6% | 87.9% | 9.1% | 0.0% |
|  | **Step 1** | 0.1% | 2.4% | 1.1% | 12.3% | 84.0% | 0.0% |
|  | **Step 0** | 0.0% | 0.2% | 0.1% | 1.2% | 1.8% | 96.7% |

Supplementary Table 22: Distribution of adult patients by step at each time interval (excluding patients with COPD)

| **Interval** | **N** | **Step 5** | **Step 4** | **Step 3** | **Step 2** | **Step 1** | **Step 0** | **Un-diagnosed** |
| --- | --- | --- | --- | --- | --- | --- | --- | --- |
| 1 - [Dec-07; Jun-07] | 420,617 | 0.006% | 11.7% | 7.5% | 22.3% | 24.4% | 12.7% | 20.9% |
| 2 - [Jun-08; Dec-07] | 432,651 | 0.006% | 12.2% | 8.0% | 23.4% | 26.5% | 11.0% | 18.3% |
| 3 - [Dec-08; Jun-08] | 445,694 | 0.006% | 12.7% | 8.5% | 24.7% | 27.8% | 9.7% | 16.1% |
| 4 - [Jun-09; Dec-08] | 456,626 | 0.006% | 13.1% | 8.8% | 25.3% | 28.9% | 9.1% | 14.2% |
| 5 - [Dec-09; Jun-09] | 469,096 | 0.007% | 13.5% | 9.2% | 26.1% | 29.5% | 8.5% | 12.5% |
| 6 - [Jun-10; Dec-09] | 478,917 | 0.007% | 13.7% | 9.6% | 26.5% | 30.3% | 8.1% | 11.0% |
| 7 - [Dec-10; Jun-10] | 490,195 | 0.007% | 13.9% | 9.9% | 27.0% | 30.8% | 7.9% | 9.8% |
| 8 - [Jun-11; Dec-10] | 499,532 | 0.007% | 14.1% | 10.2% | 27.2% | 31.6% | 7.7% | 8.6% |
| 9 - [Dec-11; Jun-11] | 510,497 | 0.008% | 14.2% | 10.5% | 27.5% | 31.9% | 7.5% | 7.7% |
| 10 - [Jun-12; Dec-11] | 519,435 | 0.008% | 14.4% | 10.7% | 27.6% | 32.6% | 7.4% | 6.6% |
| 11 - [Dec-12; Jun-12] | 529,684 | 0.008% | 14.4% | 11.2% | 27.9% | 32.9% | 7.2% | 5.5% |
| 12 - [Jun-13; Dec-12] | 537,547 | 0.008% | 14.4% | 11.6% | 28.1% | 33.4% | 7.1% | 4.5% |
| 13 - [Dec-13; Jun-13] | 546,427 | 0.008% | 14.2% | 12.2% | 28.4% | 33.7% | 7.1% | 3.7% |
| 14 - [Jun-14; Dec-13] | 553,224 | 0.008% | 14.2% | 12.6% | 28.6% | 34.1% | 7.0% | 2.7% |
| 15 - [Dec-14; Jun-14] | 560,220 | 0.008% | 14.3% | 12.9% | 28.9% | 34.3% | 6.9% | 1.9% |
| 16 - [Jun-15; Dec-14] | 565,185 | 0.008% | 14.3% | 13.3% | 28.9% | 34.8% | 6.8% | 1.1% |
| 17 - [Dec-15; Jun-15] | 565,619 | 0.008% | 14.2% | 13.6% | 29.1% | 35.0% | 6.8% | 0.5% |
| 18 - [Jun-16; Dec-15] | 565,838 | 0.006% | 14.0% | 13.7% | 29.1% | 35.8% | 6.7% | 0.1% |

Supplementary Table 23: Proportion of adult patients stepping up, down or remaining stable within a 6-month interval (patients in steps 0-5; excluding patients with COPD)

| **Interval** | **N** | **Up** | **Down** | **Stable** |
| --- | --- | --- | --- | --- |
| 1 - [Dec-07; Jun-07] | 240,367 | 10.1% | 8.0% | 81.9% |
| 2 - [Jun-08; Dec-07] | 279,529 | 8.6% | 7.4% | 83.9% |
| 3 - [Dec-08; Jun-08] | 305,872 | 8.9% | 7.1% | 84.0% |
| 4 - [Jun-09; Dec-08] | 330,980 | 7.4% | 6.5% | 86.1% |
| 5 - [Dec-09; Jun-09] | 350,282 | 7.8% | 6.4% | 85.8% |
| 6 - [Jun-10; Dec-09] | 370,691 | 6.7% | 6.0% | 87.3% |
| 7 - [Dec-10; Jun-10] | 387,030 | 6.4% | 5.6% | 88.1% |
| 8 - [Jun-11; Dec-10] | 403,485 | 5.8% | 5.5% | 88.7% |
| 9 - [Dec-11; Jun-11] | 418,161 | 5.7% | 5.0% | 89.3% |
| 10 - [Jun-12; Dec-11] | 433,024 | 5.2% | 4.9% | 89.9% |
| 11 - [Dec-12; Jun-12] | 446,826 | 5.5% | 4.9% | 89.6% |
| 12 - [Jun-13; Dec-12] | 462,158 | 4.8% | 4.6% | 90.7% |
| 13 - [Dec-13; Jun-13] | 474,842 | 4.6% | 4.3% | 91.1% |
| 14 - [Jun-14; Dec-13] | 487,849 | 4.2% | 4.0% | 91.8% |
| 15 - [Dec-14; Jun-14] | 499,603 | 3.9% | 3.5% | 92.6% |
| 16 - [Jun-15; Dec-14] | 510,893 | 3.5% | 3.4% | 93.1% |
| 17 - [Dec-15; Jun-15] | 520,318 | 3.1% | 2.9% | 94.0% |
| 18 - [Jun-16; Dec-15] | 524,568 | 2.5% | 3.1% | 94.4% |

Supplementary Table 24: Transitions between treatment steps from one interval (T-1) to the next (T), by treatment in adult patients (excluding patients with COPD)

|  | | **TO: (Time interval T)** | | | | | |
| --- | --- | --- | --- | --- | --- | --- | --- |
|  |  | **Step 5** | **Step 4** | **Step 3** | **Step 2** | **Step 1** | **Step 0** |
| **FROM: (Time interval T-1)** | **Step 5** | 80.0% | 8.6% | 3.9% | 2.6% | 4.8% | 0.0% |
|  | **Step 4** | 0.4% | 91.4% | 1.9% | 1.0% | 5.3% | 0.0% |
|  | **Step 3** | 0.2% | 1.9% | 90.6% | 1.9% | 5.3% | 0.0% |
|  | **Step 2** | 0.1% | 0.8% | 1.4% | 90.2% | 7.5% | 0.0% |
|  | **Step 1** | 0.1% | 2.3% | 2.0% | 7.1% | 88.5% | 0.0% |
|  | **Step 0** | 0.0% | 0.5% | 0.3% | 2.4% | 4.3% | 92.5% |

Supplementary Table 25: Asthma review codes

| **Cluster Name** | **Description** | **Read code** | **CTV3 code** | **Cluster Version** |
| --- | --- | --- | --- | --- |
| **Used in base case analysis** | | | | |
| REV_COD | Asthma review codes | 66YJ. , 66YK. , 66YQ. , 66YR. , 8B3j. , 9OJA. | XaIeq , XaIu5 , XaIu6 , XaIer , XaIfK , XE2Nb | 100 |
| **Used in sensitivity analysis** | | | | |
| ASTEXC_COD | Asthma exception reporting codes | 9hA..% , 9OJ2. | XaJ4W% , 9OJ2. | 100 |
| AST_COD | Asthma diagnosis codes | H33..% (excluding H333.) , H3120 , H3B.. , 173A. | H33..% (excluding H44..% , H441. , H440.% , X1025% , X1023 , XaKdk , XaJFG , Xa1hD) , X1020 , Xac33 | 101 |
| ASTSPIR_COD | Spirometry codes for Asthma | 33G1. , 33H1. , 33I1. , 33J1. , 33K1. , 745D4 , 663J. , 8HRC. | XaFrX , XaIUh , XaIUj , XaIUk , XaIUg , XaXeg , 663J. , XaK02 | 100 |
| PEFR_COD | PEFR codes | 33950 , 339n. , 339A. , 339B. , 339c. , 339d. , 339g. , 66YX. , 66YY. , 66Yc. | XaJEg , XaXHh , XaIxT , XaIxS , X77RW , XaEHe , XaEGA , XaIxD% , XaJvW | 100 |
| SMOK_COD | Smoking habit codes | 137..-137D. , 137F.-137H. , 137J. , 137K. , 137M.-137T. , 137V. , 137X.-137h. (excluding 137g.) , 137j. , 137l. , 137m. , 137o. | Ub0oo% (excluding XE0oo , XaIQi% , Ub0oq , 137L. , XaQzw , XaXP9 , XaXP8 , XaXP6 , Ub0oo , XaIuQ , Ub0p2 , Ub0p3) | 100 |
| REV_COD | Asthma review codes | 66YJ. , 66YK. , 66YQ. , 66YR. , 8B3j. , 9OJA. | XaIeq , XaIu5 , XaIu6 , XaIer , XaIfK , XE2Nb | 100 |
| RCPEXCER_COD | Asthma Exercise Codes | 6635. , 663P. , 663Q. , 663e. , 663e0 , 663e1 , 663f. , 663w. , 663x. , 663P0 , 663P1 , 663P2 | 6635. , 663P. , 663Q. , 663e. , 663e0 , 663e1 , 663f. , XaINf , XaINg , XaXZs , XaXZu , XaXZx | 100 |

Supplementary Table 26: Paediatric patients with a record of asthma annual review using all codes as per QOF business rules

| **Characteristics** | **Treatment step at reference** | | | | | | **All** |
| --- | --- | --- | --- | --- | --- | --- | --- |
|  | **Step 5** | **Step 4** | **Step 3** | **Step 2** | **Step 1** | **Step 0** |  |
| **Base case** | | | | | | | |
| Asthma annual review [July 2015-August 2016] | 7 (8.4%) | 1,065 (24.7%) | 616 (30.0%) | 3,449 (20.7%) | 2,387 (16.1%) | 11 (0.5%) | 7,535 (18.8%) |
| Asthma annual review [July 2014- August 2016] | 18 (21.7%) | 1,854 (43.0%) | 984 (47.9%) | 6,204 (37.2%) | 4,276 (28.9%) | 54 (2.6%) | 13,390 (33.4%) |
| **Sensitivity analysis** | | | | | | | |
| Asthma annual review [July 2015-August 2016] | 8 (9.6%) | 1,216 (28.2%) | 686 (33.4%) | 3,852 (23.1%) | 2,638 (17.8%) | 11 (0.5%) | 8,411 (21.0%) |
| Asthma annual review [July 2014- August 2016] | 19 (22.9%) | 2,046 (47.4%) | 1,071 (52.1%) | 6,808 (40.8%) | 4,743 (32.1%) | 60 (2.9%) | 14,747 (36.8%) |

Supplementary Table 27: Adult patients with a record of asthma annual review using all codes as per QOF business rules

| **Characteristics** | **Treatment step at reference** | | | | | | **All** |
| --- | --- | --- | --- | --- | --- | --- | --- |
|  | **Step 5** | **Step 4** | **Step 3** | **Step 2** | **Step 1** | **Step 0** |  |
| **Base case** | | | | | | | |
| Asthma annual review [July 2015-August 2016] | 584 (13.2%) | 18,109 (18.7%) | 21,264 (24.7%) | 25,611 (15.3%) | 24,335 (11.4%) | 206 (0.5%) | 90,109 (14.8%) |
| Asthma annual review [July 2014- August 2016] | 1,038 (23.5%) | 31,231 (32.2%) | 36,004 (41.9%) | 46,229 (27.7%) | 46,553 (21.8%) | 796 (2.1%) | 161,851 (26.7%) |
| **Sensitivity analysis** | | | | | | | |
| Asthma annual review [July 2015-August 2016] | 637 (14.4%) | 20,034 (20.7%) | 23,618 (27.5%) | 28,237 (16.9%) | 26,916 (12.6%) | 241 (0.6%) | 99,683 (16.4%) |
| Asthma annual review [July 2014- August 2016] | 1,131 (25.6%) | 34,010 (35.1%) | 39,170 (45.5%) | 50,360 (30.1%) | 51,160 (24.0%) | 946 (2.5%) | 176,777 (29.1%) |

Supplementary Table 28: Codes used to identify asthma

| **Medcode** | **Read code** | **Read term** |
| --- | --- | --- |
| 78 | H33..00 | Asthma |
| 81 | 663..11 | Asthma monitoring |
| 185 | H333.00 | Acute exacerbation of asthma |
| 232 | H33z100 | Asthma attack |
| 233 | H33z011 | Severe asthma attack |
| 1208 | H330.12 | Childhood asthma |
| 1555 | H33..11 | Bronchial asthma |
| 2290 | H330.11 | Allergic asthma |
| 3018 | 663V100 | Mild asthma |
| 3366 | 663V300 | Severe asthma |
| 3458 | 663V000 | Occasional asthma |
| 3665 | H331.11 | Late onset asthma |
| 4442 | H33z.00 | Asthma unspecified |
| 4606 | H33zz11 | Exercise induced asthma |
| 4892 | H33z000 | Status asthmaticus NOS |
| 5267 | H331.00 | Intrinsic asthma |
| 5627 | H330011 | Hay fever with asthma |
| 5798 | H312000 | Chronic asthmatic bronchitis |
| 5867 | 173A.00 | Exercise induced asthma |
| 6707 | H330111 | Extrinsic asthma with asthma attack |
| 7058 | 8H2P.00 | Emergency admission, asthma |
| 7146 | H330.00 | Extrinsic (atopic) asthma |
| 7191 | 663P.00 | Asthma limiting activities |
| 7378 | 663U.00 | Asthma management plan given |
| 7416 | 663N.00 | Asthma disturbing sleep |
| 7731 | H330.14 | Pollen asthma |
| 8335 | H33z111 | Asthma attack NOS |
| 8355 | 9OJA.11 | Asthma monitored |
| 9018 | 663y.00 | Number of asthma exacerbations in past year |
| 9552 | 66Y5.00 | Change in asthma management plan |
| 9663 | 66Y9.00 | Step up change in asthma management plan |
| 10043 | 66YJ.00 | Asthma annual review |
| 10274 | 8B3j.00 | Asthma medication review |
| 10487 | 663j.00 | Asthma - currently active |
| 11370 | 1O2..00 | Asthma confirmed |
| 12987 | H33z200 | Late-onset asthma |
| 13064 | 663V.00 | Asthma severity |
| 13065 | 663V200 | Moderate asthma |
| 13175 | 663N200 | Asthma disturbs sleep frequently |
| 13176 | 66YK.00 | Asthma follow-up |
| 14777 | H330000 | Extrinsic asthma without status asthmaticus |
| 15248 | H330.13 | Hay fever with asthma |
| 16070 | H33zz00 | Asthma NOS |
| 16667 | 8795 | Asthma control step 2 |
| 16785 | 8794 | Asthma control step 1 |
| 18207 | H33zz13 | Allergic bronchitis NEC |
| 18223 | 66YA.00 | Step down change in asthma management plan |
| 18224 | 8796 | Asthma control step 3 |
| 18323 | H331111 | Intrinsic asthma with asthma attack |
| 19167 | 66YQ.00 | Asthma monitoring by nurse |
| 19519 | 663p.00 | Asthma treatment compliance unsatisfactory |
| 19520 | 663n.00 | Asthma treatment compliance satisfactory |
| 20860 | 8798 | Asthma control step 5 |
| 20886 | 8797 | Asthma control step 4 |
| 21232 | H33zz12 | Allergic asthma NEC |
| 22752 | 173c.00 | Occupational asthma |
| 24479 | 663d.00 | Emergency asthma admission since last appointment |
| 24506 | 8791 | Further asthma - drug prevent. |
| 24884 | 663u.00 | Asthma causes daytime symptoms 1 to 2 times per week |
| 25181 | 663e.00 | Asthma restricts exercise |
| 25791 | 8CR0.00 | Asthma clinical management plan |
| 25796 | H332.00 | Mixed asthma |
| 26501 | 663s.00 | Asthma never causes daytime symptoms |
| 26503 | 663v.00 | Asthma causes daytime symptoms most days |
| 26504 | 663f.00 | Asthma never restricts exercise |
| 26506 | 6.63e100 | Asthma severely restricts exercise |
| 26861 | 6.63E+02 | Asthma sometimes restricts exercise |
| 27926 | H330100 | Extrinsic asthma with status asthmaticus |
| 29325 | H331000 | Intrinsic asthma without status asthmaticus |
| 30458 | 66YR.00 | Asthma monitoring by doctor |
| 30815 | 663N000 | Asthma causing night waking |
| 31167 | 66YP.00 | Asthma night-time symptoms |
| 31225 | 663t.00 | Asthma causes daytime symptoms 1 to 2 times per month |
| 32727 | H33z.00 | Hyperactive airway disease |
| 38143 | 663O000 | Asthma never disturbs sleep |
| 38144 | 663w.00 | Asthma limits walking up hills or stairs |
| 38145 | 663x.00 | Asthma limits walking on the flat |
| 38146 | 663N100 | Asthma disturbs sleep weekly |
| 39478 | H35y700 | Wood asthma |
| 39570 | 663r.00 | Asthma causes night symptoms 1 to 2 times per month |
| 40823 | H334.00 | Brittle asthma |
| 41017 | 1780 | Aspirin induced asthma |
| 41020 | 66YC.00 | Absent from work or school due to asthma |
| 42824 | 663q.00 | Asthma daytime symptoms |
| 45073 | H331z00 | Intrinsic asthma NOS |
| 45782 | H330z00 | Extrinsic asthma NOS |
| 46529 | 9OJ1.00 | Attends asthma monitoring |
| 47337 | 663m.00 | Asthma accident and emergency attendance since last visit |
| 47684 | H47y000 | Detergent asthma |
| 58196 | H331100 | Intrinsic asthma with status asthmaticus |
| 73522 | 173d.00 | Work aggravated asthma |
| 93353 | H35y600 | Sequoiosis (red-cedar asthma) |
| 93736 | 388t.00 | Royal College of Physicians asthma assessment |
| 98185 | 38DL.00 | Asthma control test |
| 99793 | 8CMA000 | Patient has a written asthma personal action plan |
| 100107 | 679J000 | Health education - asthma self management |
| 100397 | 38DT.00 | Asthma control questionnaire |
| 100509 | 9NNX.00 | Under care of asthma specialist nurse |
| 100740 | 679J100 | Health education - structured asthma discussion |
| 102170 | 66Yp.00 | Asthma review using Roy College of Physicians three questions |
| 102209 | 38DV.00 | Mini asthma quality of life questionnaire |
| 102301 | 1787 | Asthma trigger - seasonal |
| 102341 | 1781 | Asthma trigger - pollen |
| 102395 | 66Yr.00 | Asthma causes symptoms most nights |
| 102400 | 66Yq.00 | Asthma causes night time symptoms 1 to 2 times per week |
| 102449 | 1789 | Asthma trigger - respiratory infection |
| 102713 | 663P000 | Asthma limits activities 1 to 2 times per month |
| 102871 | 178B.00 | Asthma trigger - exercise |
| 102888 | 663P100 | Asthma limits activities 1 to 2 times per week |
| 102952 | 1783 | Asthma trigger - warm air |
| 103318 | 679J200 | Health education - structured patient focused asthma discuss |
| 103321 | 1786 | Asthma trigger - animals |
| 103612 | 66Ys.00 | Asthma never causes night symptoms |
| 103631 | 388t000 | Royal College Physician asthma assessment 3 question score |
| 103813 | 1788 | Asthma trigger - cold air |
| 103944 | 178A.00 | Asthma trigger - airborne dust |
| 103945 | 1785 | Asthma trigger - damp |
| 103952 | 1784 | Asthma trigger - emotion |
| 103955 | 1782 | Asthma trigger - tobacco smoke |
| 103998 | 663P200 | Asthma limits activities most days |
| 105420 | 661N100 | Asthma self-management plan review |
| 105674 | 661M100 | Asthma self-management plan agreed |
| 106805 | H335.00 | Chronic asthma with fixed airflow obstruction |
| 107167 | 66Yu.00 | Number days absent from school due to asthma in past 6 month |
| 109958 | H3B..00 | Asthma-chronic obstructive pulmonary disease overlap syndrome |

Supplementary Table 29: ICS product codes

| **Product code** | **Product name** | **Drug substance name** | **Substance strength** | **ICS per dose (mcg)** | **BNF code** | **BDP per dose (mcg)** |
| --- | --- | --- | --- | --- | --- | --- |
| 1861 | AeroBec 100 Autohaler (Meda Pharmaceuticals Ltd) | Beclometasone dipropionate | 100 mcg/1 dose | 100 | 3020000 | 100 |
| 4499 | Aerobec 250 mcg/actuation Pressurised inhalation (Meda Pharmaceuticals Ltd) | Beclometasone dipropionate | 250 mcg/1 dose | 250 | 3020000 | 250 |
| 2159 | AeroBec 50 Autohaler (Meda Pharmaceuticals Ltd) | Beclometasone dipropionate | 50 mcg/1 dose | 50 | 3020000 | 50 |
| 39200 | AeroBec Forte 250 Autohaler (Meda Pharmaceuticals Ltd) | Beclometasone dipropionate | 250 mcg/1 dose | 250 | 3020000 | 250 |
| 6839 | Alvesco 160 inhaler (Takeda UK Ltd) | Ciclesonide | 160 mcg/1 dose | 160 | 3020000 | 213.3333328 |
| 21224 | Alvesco 80 inhaler (Takeda UK Ltd) | Ciclesonide | 80 mcg/1 dose | 80 | 3020000 | 106.6666664 |
| 4601 | Asmabec 100 Clickhaler (Focus Pharmaceuticals Ltd) | Beclometasone dipropionate | 100 mcg/1 dose | 100 | 3020000 | 100 |
| 9477 | Asmabec 100 mcg/actuation Spacehaler (Celltech Pharma Europe Ltd) | Beclometasone Dipropionate | 100 mcg/actuation | 100 | 3020000 | 100 |
| 14567 | Asmabec 250 Clickhaler (Focus Pharmaceuticals Ltd) | Beclometasone dipropionate | 250 mcg/1 dose | 250 | 3020000 | 250 |
| 14590 | Asmabec 250 mcg/actuation Spacehaler (Celltech Pharma Europe Ltd) | Beclometasone Dipropionate | 250 mcg/actuation | 250 | 3020000 | 250 |
| 9577 | Asmabec 50 Clickhaler (Focus Pharmaceuticals Ltd) | Beclometasone dipropionate | 50 mcg/1 dose | 50 | 3020000 | 50 |
| 19389 | Asmabec 50 mcg/actuation Spacehaler (Celltech Pharma Europe Ltd) | Beclometasone Dipropionate | 50 mcg/actuation | 50 | 3020000 | 50 |
| 16433 | Asmanex 200 mcgs/dose Twisthaler (Merck Sharp & Dohme Ltd) | Mometasone furoate | 200 mcgs/1 dose | 400 | 3020000 | 800 |
| 17590 | Asmanex 400 mcgs/dose Twisthaler (Merck Sharp & Dohme Ltd) | Mometasone furoate | 400 mcgs/1 dose | 400 | 3020000 | 800 |
| 19031 | Bdp 100 mcg/actuation Spacehaler (Celltech Pharma Europe Ltd) | Beclometasone Dipropionate | 100 mcg/actuation | 100 | 3020000 | 100 |
| 14524 | Bdp 250 mcg/actuation Spacehaler (Celltech Pharma Europe Ltd) | Beclometasone Dipropionate | 250 mcg/actuation | 250 | 3020000 | 250 |
| 18394 | Bdp 50 mcg/actuation Spacehaler (Celltech Pharma Europe Ltd) | Beclometasone Dipropionate | 50 mcg/actuation | 50 | 3020000 | 50 |
| 895 | Beclazone 100 Easi-Breathe inhaler (Teva UK Ltd) | Beclometasone dipropionate | 100 mcg/1 dose | 100 | 3020000 | 100 |
| 1100 | Beclazone 100 inhaler (Teva UK Ltd) | Beclometasone dipropionate | 100 mcg/1 dose | 100 | 3020000 | 100 |
| 13815 | Beclazone 100 mcg/actuation Inhalation powder (Actavis UK Ltd) | Beclometasone dipropionate | 100 mcg/1 dose | 100 | 3020000 | 100 |
| 1885 | Beclazone 200 inhaler (Teva UK Ltd) | Beclometasone dipropionate | 200 mcg/1 dose | 250 | 3020000 | 250 |
| 1243 | Beclazone 250 Easi-Breathe inhaler (Teva UK Ltd) | Beclometasone dipropionate | 250 mcg/1 dose | 250 | 3020000 | 250 |
| 1551 | Beclazone 250 inhaler (Teva UK Ltd) | Beclometasone dipropionate | 250 mcg/1 dose | 250 | 3020000 | 250 |
| 4803 | Beclazone 250 mcg/actuation Inhalation powder (Actavis UK Ltd) | Beclometasone dipropionate | 250 mcg/1 dose | 250 | 3020000 | 250 |
| 1725 | Beclazone 50 Easi-Breathe inhaler (Teva UK Ltd) | Beclometasone dipropionate | 50 mcg/1 dose | 50 | 3020000 | 50 |
| 2992 | Beclazone 50 inhaler (Teva UK Ltd) | Beclometasone dipropionate | 50 mcg/1 dose | 50 | 3020000 | 50 |
| 9599 | Beclazone 50 mcg/actuation Inhalation powder (Actavis UK Ltd) | Beclometasone dipropionate | 50 mcg/1 dose | 50 | 3020000 | 50 |
| 47943 | Beclazone easi-breathe (roi) 100 mcg/actuation Pressurised inhalation (Ivax Pharmaceuticals Ireland) | Beclometasone Dipropionate | 100 mcg/actuation | 100 | 3020000 | 100 |
| 67735 | Beclazone easi-breathe (roi) 250 mcg/actuation Pressurised inhalation (Ivax Pharmaceuticals Ireland) | Beclometasone Dipropionate | 250 mcg/actuation |  | 3020000 |  |
| 57589 | Becloforte 250 mcgs/dose inhaler (Dowelhurst Ltd) | Beclometasone dipropionate | 250 mcg/1 dose |  | 3020000 |  |
| 1236 | Becloforte 250 mcgs/dose inhaler (GlaxoSmithKline UK Ltd) | Beclometasone dipropionate | 250 mcg/1 dose | 250 | 3020000 | 250 |
| 2892 | Becloforte 400 mcg disks (GlaxoSmithKline UK Ltd) | Beclometasone dipropionate | 400 mcg | 400 | 3020000 | 400 |
| 3363 | Becloforte 400 mcg disks with Diskhaler (GlaxoSmithKline UK Ltd) | Beclometasone dipropionate | 400 mcg | 400 | 3020000 | 400 |
| 1552 | Becloforte easi-breathe 250 mcg/actuation Pressurised inhalation (Allen & Hanburys Ltd) | Beclometasone dipropionate | 250 mcg/1 dose | 250 | 3020000 | 250 |
| 3119 | Becloforte integra 250 mcg/actuation Inhaler with compact spacer (Glaxo Laboratories Ltd) | Beclometasone Dipropionate | 250 mcg/actuation | 250 | 3020000 | 250 |
| 8111 | Becloforte vm 250 mcg/actuation VM pack (Allen & Hanburys Ltd) | Beclometasone dipropionate | 250 mcg/1 dose | 250 | 3020000 | 250 |
| 15706 | Beclometasone 100 mcgs/actuation vortex inhaler | Beclometasone Dipropionate | 100 mcgs/actuation | 100 | 3020000 | 100 |
| 35652 | Beclometasone 100 mcg inhalation powder blisters | Beclometasone dipropionate | 100 mcg | 100 | 3020000 | 100 |
| 35580 | Beclometasone 100 mcg inhalation powder blisters with device | Beclometasone dipropionate | 100 mcg | 100 | 3020000 | 100 |
| 4759 | Beclometasone 100 mcg inhalation powder capsules | Beclometasone dipropionate | 100 mcg | 100 | 3020000 | 100 |
| 28640 | Beclometasone 100 mcg/actuation Inhalation powder (Actavis UK Ltd) | Beclometasone dipropionate | 100 mcg/1 dose | 100 | 3020000 | 100 |
| 33849 | Beclometasone 100 mcg/actuation Inhalation powder (Neo Laboratories Ltd) | Beclometasone dipropionate | 100 mcg/1 dose | 100 | 3020000 | 100 |
| 27679 | Beclometasone 100 mcg/actuation Pressurised inhalation (Approved Prescription Services Ltd) | Beclometasone dipropionate | 100 mcg/1 dose | 100 | 3020000 | 100 |
| 4365 | Beclometasone 100 mcgs disc | Beclometasone Dipropionate | 100 mcgs | 100 | 3020000 | 100 |
| 19121 | Beclometasone 100 mcgs with Salbutamol 200 mcgs inhalation capsules | Beclometasone Dipropionate | 100 mcgs + 200 mcgs | 100 | 03010101/03020000 | 100 |
| 3150 | Beclometasone 100 mcgs/actuation extrafine particle cfc free inhaler | Beclometasone Dipropionate | 100 mcgs/actuation | 100 | 3020000 | 100 |
| 1734 | Beclometasone 100 mcgs/dose breath actuated inhaler | Beclometasone dipropionate | 100 mcg/1 dose | 100 | 3020000 | 100 |
| 9921 | Beclometasone 100 mcgs/dose breath actuated inhaler CFC free | Beclometasone dipropionate | 100 mcg/1 dose | 100 | 3020000 | 100 |
| 5522 | Beclometasone 100 mcgs/dose dry powder inhaler | Beclometasone dipropionate | 100 mcg/1 dose | 100 | 3020000 | 100 |
| 38 | Beclometasone 100 mcgs/dose inhaler | Beclometasone dipropionate | 100 mcg/1 dose | 100 | 3020000 | 100 |
| 25204 | Beclometasone 100 mcgs/dose inhaler (A A H Pharmaceuticals Ltd) | Beclometasone dipropionate | 100 mcg/1 dose | 100 | 3020000 | 100 |
| 21482 | Beclometasone 100 mcgs/dose inhaler (Generics (UK) Ltd) | Beclometasone dipropionate | 100 mcg/1 dose | 100 | 3020000 | 100 |
| 26063 | Beclometasone 100 mcgs/dose inhaler (Teva UK Ltd) | Beclometasone dipropionate | 100 mcg/1 dose | 100 | 3020000 | 100 |
| 15326 | Beclometasone 100 mcgs/dose inhaler CFC free | Beclometasone dipropionate | 100 mcg/1 dose | 100 | 3020000 | 100 |
| 62518 | Beclometasone 100 mcgs/dose inhaler CFC free (Ennogen Healthcare Ltd) | Beclometasone dipropionate | 100 mcg/1 dose |  | 3020000 |  |
| 46157 | Beclometasone 200 Cyclocaps (Teva UK Ltd) | Beclometasone dipropionate | 200 mcg | 200 | 3020000 | 200 |
| 35113 | Beclometasone 200 mcg inhalation powder blisters | Beclometasone dipropionate | 200 mcg | 200 | 3020000 | 200 |
| 35293 | Beclometasone 200 mcg inhalation powder blisters with device | Beclometasone dipropionate | 200 mcg | 200 | 3020000 | 200 |
| 9233 | Beclometasone 200 mcg inhalation powder capsules | Beclometasone dipropionate | 200 mcg | 200 | 3020000 | 200 |
| 2893 | Beclometasone 200 mcgs disc | Beclometasone Dipropionate | 200 mcgs | 200 | 3020000 | 200 |
| 19376 | Beclometasone 200 mcgs with Salbutamol 400 mcgs inhalation capsules | Beclometasone Dipropionate | 200 mcgs + 400 mcgs | 200 | 03010101/03020000 | 200 |
| 5521 | Beclometasone 200 mcgs/dose dry powder inhaler | Beclometasone dipropionate | 200 mcg/1 dose | 200 | 3020000 | 200 |
| 1259 | Beclometasone 200 mcgs/dose inhaler | Beclometasone dipropionate | 200 mcg/1 dose | 200 | 3020000 | 200 |
| 34794 | Beclometasone 200 mcgs/dose inhaler (A A H Pharmaceuticals Ltd) | Beclometasone dipropionate | 200 mcg/1 dose | 200 | 3020000 | 200 |
| 14321 | Beclometasone 200 mcgs/dose inhaler CFC free | Beclometasone Dipropionate | 200 mcgs/actuation | 200 | 3020000 | 200 |
| 34315 | Beclometasone 250 mcg/actuation Inhalation powder (Actavis UK Ltd) | Beclometasone dipropionate | 250 mcg/1 dose | 250 | 3020000 | 250 |
| 34859 | Beclometasone 250 mcg/actuation Inhalation powder (Neo Laboratories Ltd) | Beclometasone dipropionate | 250 mcg/1 dose | 250 | 3020000 | 250 |
| 28073 | Beclometasone 250 mcg/actuation Pressurised inhalation (Approved Prescription Services Ltd) | Beclometasone dipropionate | 250 mcg/1 dose | 250 | 3020000 | 250 |
| 19401 | Beclometasone 250 mcgs/actuation inhaler and compact spacer | Beclometasone Dipropionate | 250 mcgs/actuation | 250 | 3020000 | 250 |
| 9571 | Beclometasone 250 mcgs/actuation vortex inhaler | Beclometasone Dipropionate | 250 mcgs/actuation | 250 | 3020000 | 250 |
| 2600 | Beclometasone 250 mcgs/dose breath actuated inhaler | Beclometasone dipropionate | 250 mcg/1 dose | 250 | 3020000 | 250 |
| 5804 | Beclometasone 250 mcgs/dose dry powder inhaler | Beclometasone dipropionate | 250 mcg/1 dose | 250 | 3020000 | 250 |
| 1242 | Beclometasone 250 mcgs/dose inhaler | Beclometasone dipropionate | 250 mcg/1 dose | 250 | 3020000 | 250 |
| 33258 | Beclometasone 250 mcgs/dose inhaler (A A H Pharmaceuticals Ltd) | Beclometasone dipropionate | 250 mcg/1 dose | 250 | 3020000 | 250 |
| 29325 | Beclometasone 250 mcgs/dose inhaler (Generics (UK) Ltd) | Beclometasone dipropionate | 250 mcg/1 dose | 250 | 3020000 | 250 |
| 30210 | Beclometasone 250 mcgs/dose inhaler (Teva UK Ltd) | Beclometasone dipropionate | 250 mcg/1 dose | 250 | 3020000 | 250 |
| 21005 | Beclometasone 250 mcgs/dose inhaler CFC free | Beclometasone dipropionate | 250 mcg/1 dose | 250 | 3020000 | 250 |
| 41269 | Beclometasone 400 Cyclocaps (Teva UK Ltd) | Beclometasone dipropionate | 400 mcg | 400 | 3020000 | 400 |
| 2148 | Beclometasone 400 mcg disc | Beclometasone Dipropionate | 400 mcgs | 400 | 3020000 | 400 |
| 35288 | Beclometasone 400 mcg inhalation powder blisters | Beclometasone dipropionate | 400 mcg | 400 | 3020000 | 400 |
| 35107 | Beclometasone 400 mcg inhalation powder blisters with device | Beclometasone dipropionate | 400 mcg | 400 | 3020000 | 400 |
| 7653 | Beclometasone 400 mcg inhalation powder capsules | Beclometasone dipropionate | 400 mcg | 400 | 3020000 | 400 |
| 41412 | Beclometasone 400 mcgs/actuation inhaler | Beclometasone Dipropionate | 400 mcgs/actuation | 400 | 3020000 | 400 |
| 11497 | Beclometasone 400 mcgs/dose dry powder inhaler | Beclometasone dipropionate | 400 mcg/1 dose | 400 | 3020000 | 400 |
| 32874 | Beclometasone 50 mcg/actuation Inhalation powder (Actavis UK Ltd) | Beclometasone dipropionate | 50 mcg/1 dose | 50 | 3020000 | 50 |
| 34428 | Beclometasone 50 mcg/actuation Inhalation powder (Neo Laboratories Ltd) | Beclometasone dipropionate | 50 mcg/1 dose | 50 | 3020000 | 50 |
| 30238 | Beclometasone 50 mcg/actuation Pressurised inhalation (Approved Prescription Services Ltd) | Beclometasone dipropionate | 50 mcg/1 dose | 50 | 3020000 | 50 |
| 3556 | Beclometasone 50 mcgs with salbutamol 100 mcgs/inhalation inhaler | Beclometasone Dipropionate | 50 mcgs + 100 mcgs/inhalation | 50 | 03010101/03020000 | 50 |
| 10090 | Beclometasone 50 mcgs/actuation extrafine particle cfc free inhaler | Beclometasone Dipropionate | 50 mcgs/actuation | 50 | 3020000 | 50 |
| 2160 | Beclometasone 50 mcgs/dose breath actuated inhaler | Beclometasone dipropionate | 50 mcg/1 dose | 50 | 3020000 | 50 |
| 11732 | Beclometasone 50 mcgs/dose breath actuated inhaler CFC free | Beclometasone dipropionate | 50 mcg/1 dose | 50 | 3020000 | 50 |
| 5992 | Beclometasone 50 mcgs/dose dry powder inhaler | Beclometasone dipropionate | 50 mcg/1 dose | 50 | 3020000 | 50 |
| 3018 | Beclometasone 50 mcgs/dose inhaler | Beclometasone dipropionate | 50 mcg/1 dose | 50 | 3020000 | 50 |
| 34919 | Beclometasone 50 mcgs/dose inhaler (A A H Pharmaceuticals Ltd) | Beclometasone dipropionate | 50 mcg/1 dose | 50 | 3020000 | 50 |
| 63585 | Beclometasone 50 mcgs/dose inhaler (Almus Pharmaceuticals Ltd) | Beclometasone dipropionate | 50 mcg/1 dose |  | 3020000 |  |
| 31774 | Beclometasone 50 mcgs/dose inhaler (Generics (UK) Ltd) | Beclometasone dipropionate | 50 mcg/1 dose | 50 | 3020000 | 50 |
| 34739 | Beclometasone 50 mcgs/dose inhaler (Teva UK Ltd) | Beclometasone dipropionate | 50 mcg/1 dose | 50 | 3020000 | 50 |
| 16584 | Beclometasone 50 mcgs/dose inhaler CFC free | Beclometasone dipropionate | 50 mcg/1 dose | 50 | 3020000 | 50 |
| 7964 | Beclometasone 50 mcgs/ml nebuliser suspension | Beclometasone Dipropionate | 50 mcgs/ml | 50 | 3020000 | 50 |
| 11198 | Beclometasons 50 mcgs/actuation vortex inhaler | Beclometasone Dipropionate | 50 mcgs/actuation | 50 | 3020000 | 50 |
| 35408 | Becodisks 100 mcg (GlaxoSmithKline UK Ltd) | Beclometasone dipropionate | 100 mcg | 100 | 3020000 | 100 |
| 2229 | Becodisks 100 mcg Disc (Allen & Hanburys Ltd) | Beclometasone Dipropionate | 100 mcg | 100 | 3020000 | 100 |
| 35106 | Becodisks 100 mcg with Diskhaler (GlaxoSmithKline UK Ltd) | Beclometasone dipropionate | 100 mcg | 100 | 3020000 | 100 |
| 35071 | Becodisks 200 mcg (GlaxoSmithKline UK Ltd) | Beclometasone dipropionate | 200 mcg | 200 | 3020000 | 200 |
| 67265 | Becodisks 200 mcg (Lexon (UK) Ltd) | Beclometasone dipropionate | 200 mcg |  | 3020000 |  |
| 56471 | Becodisks 200 mcg (Mawdsley-Brooks & Company Ltd) | Beclometasone dipropionate | 200 mcg |  | 3020000 |  |
| 883 | Becodisks 200 mcg Disc (Allen & Hanburys Ltd) | Beclometasone Dipropionate | 200 mcg | 200 | 3020000 | 200 |
| 35430 | Becodisks 200 mcg with Diskhaler (GlaxoSmithKline UK Ltd) | Beclometasone dipropionate | 200 mcg | 200 | 3020000 | 200 |
| 35299 | Becodisks 400 mcg (GlaxoSmithKline UK Ltd) | Beclometasone dipropionate | 400 mcg | 400 | 3020000 | 400 |
| 56462 | Becodisks 400 mcg (Waymade Healthcare Plc) | Beclometasone dipropionate | 400 mcg |  | 3020000 |  |
| 1951 | Becodisks 400 mcg Disc (Allen & Hanburys Ltd) | Beclometasone Dipropionate | 400 mcg | 400 | 3020000 | 400 |
| 35118 | Becodisks 400 mcg with Diskhaler (GlaxoSmithKline UK Ltd) | Beclometasone dipropionate | 400 mcg | 400 | 3020000 | 400 |
| 99 | Becotide 100 inhaler (GlaxoSmithKline UK Ltd) | Beclometasone dipropionate | 100 mcg/1 dose | 100 | 3020000 | 100 |
| 67234 | Becotide 100 inhaler (Waymade Healthcare Plc) | Beclometasone dipropionate | 100 mcg/1 dose |  | 3020000 |  |
| 3947 | Becotide 100 mcg Rotacaps (GlaxoSmithKline UK Ltd) | Beclometasone dipropionate | 100 mcg | 100 | 3020000 | 100 |
| 1258 | Becotide 200 inhaler (GlaxoSmithKline UK Ltd) | Beclometasone dipropionate | 200 mcg/1 dose | 200 | 3020000 | 200 |
| 1537 | Becotide 200 mcg Rotacaps (GlaxoSmithKline UK Ltd) | Beclometasone dipropionate | 200 mcg | 200 | 3020000 | 200 |
| 3075 | Becotide 400 mcg Rotacaps (GlaxoSmithKline UK Ltd) | Beclometasone dipropionate | 400 mcg | 400 | 3020000 | 400 |
| 62341 | Becotide 50 inhaler (Dowelhurst Ltd) | Beclometasone dipropionate | 50 mcg/1 dose |  | 3020000 |  |
| 1406 | Becotide 50 inhaler (GlaxoSmithKline UK Ltd) | Beclometasone dipropionate | 50 mcg/1 dose | 50 | 3020000 | 50 |
| 1269 | Becotide 50 mcg/ml Nebuliser liquid (Allen & Hanburys Ltd) | Beclometasone Dipropionate | 50 mcg/ml | 50 | 3020000 | 50 |
| 896 | Becotide easi-breathe 100 mcg/actuation Pressurised inhalation (Allen & Hanburys Ltd) | Beclometasone dipropionate | 100 mcg/1 dose | 100 | 3020000 | 100 |
| 1727 | Becotide easi-breathe 50 mcg/actuation Pressurised inhalation (Allen & Hanburys Ltd) | Beclometasone dipropionate | 50 mcg/1 dose | 50 | 3020000 | 50 |
| 7724 | Betamethasone valerate 100 mcgs/actuation inhaler | Betamethasone Valerate | 100 mcgs/actuation |  | 3020000 |  |
| 3065 | Bextasol Inhalation powder (Allen & Hanburys Ltd) | Betamethasone Valerate |  |  | 3020000 |  |
| 35631 | Budelin Novolizer 200 mcgs/dose inhalation powder (Meda Pharmaceuticals Ltd) | Budesonide | 200 mcg/1 dose | 200 | 3020000 | 200 |
| 35724 | Budelin Novolizer 200 mcgs/dose inhalation powder refill (Meda Pharmaceuticals Ltd) | Budesonide | 200 mcg/1 dose | 200 | 3020000 | 200 |
| 8433 | Budesonide 100 mcgs/actuation inhaler | Budesonide | 100 mcgs/actuation | 100 | 3020000 | 100 |
| 7788 | Budesonide 100 mcgs/dose dry powder inhaler | Budesonide | 100 mcg/1 dose | 100 | 3020000 | 100 |
| 39102 | Budesonide 100 mcgs/dose inhaler CFC free | Budesonide | 100 mcgs | 100 | 0 | 100 |
| 4942 | Budesonide 1mg/2ml nebuliser liquid unit dose vials | Budesonide | 0.5 mcg/1ml | 1000 | 3020000 | 1000 |
| 65448 | Budesonide 1mg/2ml nebuliser liquid unit dose vials (A A H Pharmaceuticals Ltd) | Budesonide | 500 mcg/1ml |  | 3020000 |  |
| 18537 | Budesonide 200 mcg inhalation powder capsules | Budesonide | 200 mcg | 200 | 0 | 200 |
| 16054 | Budesonide 200 mcgs/actuation breath actuated powder inhaler | Budesonide | 200 mcgs/actuation | 200 | 3020000 | 200 |
| 3570 | Budesonide 200 mcgs/actuation refill canister | Budesonide | 200 mcgs/actuation | 200 | 3020000 | 200 |
| 35602 | Budesonide 200 mcgs/dose dry powder inhalation cartridge | Budesonide | 200 mcgs | 200 | 3020000 | 200 |
| 35510 | Budesonide 200 mcgs/dose dry powder inhalation cartridge with device | Budesonide | 200 mcg/1 dose | 200 | 3020000 | 200 |
| 2092 | Budesonide 200 mcgs/dose dry powder inhaler | Budesonide | 200 mcg/1 dose | 200 | 3020000 | 200 |
| 909 | Budesonide 200 mcgs/dose inhaler | Budesonide | 200 mcg/1 dose | 200 | 0 | 200 |
| 39879 | Budesonide 200 mcgs/dose inhaler CFC free | Budesonide | 200 mcg/1 dose | 200 | 0 | 200 |
| 10321 | Budesonide 400 mcg inhalation powder capsules | Budesonide | 400 mcg | 400 | 0 | 400 |
| 14700 | Budesonide 400 mcgs/actuation inhaler | Budesonide | 400 mcgs/actuation | 400 | 3020000 | 400 |
| 1642 | Budesonide 400 mcgs/dose dry powder inhaler | Budesonide | 400 mcg/1 dose | 400 | 3020000 | 400 |
| 67315 | Budesonide 400 mcgs/dose Turbohaler (Waymade Healthcare Plc) | Budesonide | 400 mcg/1 dose |  | 3020000 |  |
| 4801 | Budesonide 500 mcgs/2ml nebuliser liquid unit dose vials | Budesonide | 2.5 mcg/1ml | 500 | 3020000 | 500 |
| 61975 | Budesonide 500 mcgs/2ml nebuliser liquid unit dose vials (Almus Pharmaceuticals Ltd) | Budesonide | 250 mcg/1ml |  | 3020000 |  |
| 947 | Budesonide 50 mcgs/actuation refill canister | Budesonide | 50 mcgs/actuation | 50 | 3020000 | 50 |
| 959 | Budesonide 50 mcgs/dose inhaler | Budesonide | 50 mcg/1 dose | 50 | 0 | 50 |
| 10102 | Ciclesonide 160 mcgs/dose inhaler CFC free | Ciclesonide | 160 mcg/1 dose | 160 | 3020000 | 213.3333328 |
| 7356 | Ciclesonide 80 mcgs/dose inhaler CFC free | Ciclesonide | 80 mcgs/1 dose | 80 | 3020000 | 106.6666664 |
| 13290 | Clenil Modulite 100 mcgs/dose inhaler (Chiesi Ltd) | Beclometasone dipropionate | 100 mcg/1 dose | 100 | 3020000 | 100 |
| 48340 | Clenil Modulite 100 mcgs/dose inhaler (Mawdsley-Brooks & Company Ltd) | Beclometasone dipropionate | 100 mcg/1 dose | 100 | 3020000 | 100 |
| 16151 | Clenil Modulite 200 mcgs/dose inhaler (Chiesi Ltd) | Beclometasone Dipropionate | 200 mcgs/actuation | 200 | 3020000 | 200 |
| 49412 | Clenil Modulite 200 mcgs/dose inhaler (Mawdsley-Brooks & Company Ltd) |  |  |  | 3020000 |  |
| 16148 | Clenil Modulite 250 mcgs/dose inhaler (Chiesi Ltd) | Beclometasone dipropionate | 250 mcg/1 dose | 250 | 3020000 | 250 |
| 61664 | Clenil Modulite 250 mcgs/dose inhaler (Waymade Healthcare Plc) | Beclometasone dipropionate | 250 mcg/1 dose |  | 3020000 |  |
| 16158 | Clenil Modulite 50 mcgs/dose inhaler (Chiesi Ltd) | Beclometasone dipropionate | 50 mcg/1 dose | 50 | 3020000 | 50 |
| 49367 | Clenil Modulite 50 mcgs/dose inhaler (Mawdsley-Brooks & Company Ltd) | Beclometasone dipropionate | 50 mcg/1 dose | 50 | 3020000 | 50 |
| 17654 | Easyhaler Beclometasone 200 mcgs/dose dry powder inhaler (Orion Pharma (UK) Ltd) | Beclometasone dipropionate | 200 mcg/1 dose | 200 | 3020000 | 200 |
| 17670 | Easyhaler Budesonide 100 mcgs/dose dry powder inhaler (Orion Pharma (UK) Ltd) | Budesonide | 100 mcg/1 dose | 100 | 3020000 | 100 |
| 27188 | Easyhaler Budesonide 200 mcgs/dose dry powder inhaler (Orion Pharma (UK) Ltd) | Budesonide | 200 mcg/1 dose | 200 | 3020000 | 200 |
| 30649 | Easyhaler Budesonide 400 mcgs/dose dry powder inhaler (Orion Pharma (UK) Ltd) | Budesonide | 400 mcg/1 dose | 400 | 3020000 | 400 |
| 3927 | Filair 100 inhaler (Meda Pharmaceuticals Ltd) | Beclometasone dipropionate | 100 mcg/1 dose | 100 | 3020000 | 100 |
| 3743 | Filair 50 inhaler (Meda Pharmaceuticals Ltd) | Beclometasone dipropionate | 50 mcg/1 dose | 50 | 3020000 | 50 |
| 3993 | Filair Forte 250 mcgs/dose inhaler (Meda Pharmaceuticals Ltd) | Beclometasone dipropionate | 250 mcg/1 dose | 250 | 3020000 | 250 |
| 5551 | Flixotide 0.5mg/2ml Nebules (GlaxoSmithKline UK Ltd) | Fluticasone propionate | 250 mcg/1ml | 500 | 3020000 | 1000 |
| 67319 | Flixotide 0.5mg/2ml Nebules (Waymade Healthcare Plc) | Fluticasone propionate | 250 mcg/1ml |  | 3020000 |  |
| 3989 | Flixotide 100 mcg Disc (Allen & Hanburys Ltd) | Fluticasone Propionate | 100 mcg | 100 | 3020000 | 200 |
| 36090 | Flixotide 100 mcg disks (GlaxoSmithKline UK Ltd) | Fluticasone propionate | 100 mcg | 100 | 3020000 | 200 |
| 35225 | Flixotide 100 mcg disks with Diskhaler (GlaxoSmithKline UK Ltd) | Fluticasone propionate | 100 mcg | 100 | 3020000 | 200 |
| 42928 | Flixotide 100 mcgs/dose Accuhaler (GlaxoSmithKline UK Ltd) | Fluticasone propionate | 100 mcg/1 dose | 100 | 3020000 | 200 |
| 56477 | Flixotide 100 mcgs/dose Accuhaler (Waymade Healthcare Plc) | Fluticasone propionate | 100 mcg/1 dose |  | 3020000 |  |
| 1676 | Flixotide 125 mcg/actuation Inhalation powder (Allen & Hanburys Ltd) | Fluticasone Propionate | 125 mcg/actuation | 125 | 3020000 | 250 |
| 56474 | Flixotide 125 mcgs/dose Evohaler (DE Pharmaceuticals) | Fluticasone propionate | 125 mcg/1 dose |  | 3020000 |  |
| 57555 | Flixotide 125 mcgs/dose Evohaler (Dowelhurst Ltd) | Fluticasone propionate | 125 mcg/1 dose |  | 3020000 |  |
| 5718 | Flixotide 125 mcgs/dose Evohaler (GlaxoSmithKline UK Ltd) | Fluticasone propionate | 125 mcg/1 dose | 125 | 3020000 | 250 |
| 67237 | Flixotide 125 mcgs/dose Evohaler (Lexon (UK) Ltd) | Fluticasone propionate | 125 mcg/1 dose |  | 3020000 |  |
| 1424 | Flixotide 250 mcg Disc (Allen & Hanburys Ltd) | Fluticasone Propionate | 250 mcg | 250 | 3020000 | 500 |
| 35611 | Flixotide 250 mcg disks (GlaxoSmithKline UK Ltd) | Fluticasone propionate | 250 mcg | 250 | 3020000 | 500 |
| 35461 | Flixotide 250 mcg disks with Diskhaler (GlaxoSmithKline UK Ltd) | Fluticasone propionate | 250 mcg | 250 | 3020000 | 500 |
| 1412 | Flixotide 250 mcg/actuation Inhalation powder (Allen & Hanburys Ltd) | Fluticasone Propionate | 250 mcg/actuation | 250 | 3020000 | 500 |
| 42994 | Flixotide 250 mcgs/dose Accuhaler (GlaxoSmithKline UK Ltd) | Fluticasone propionate | 250 mcg/1 dose | 250 | 3020000 | 500 |
| 57525 | Flixotide 250 mcgs/dose Accuhaler (Stephar (U.K.) Ltd) | Fluticasone propionate | 250 mcg/1 dose |  | 3020000 |  |
| 56484 | Flixotide 250 mcgs/dose Accuhaler (Waymade Healthcare Plc) | Fluticasone propionate | 250 mcg/1 dose |  | 3020000 |  |
| 5683 | Flixotide 250 mcgs/dose Evohaler (GlaxoSmithKline UK Ltd) | Fluticasone propionate | 250 mcg/1 dose | 250 | 3020000 | 500 |
| 51815 | Flixotide 250 mcgs/dose Evohaler (Waymade Healthcare Plc) | Fluticasone propionate | 250 mcg/1 dose | 250 | 3020000 | 500 |
| 3289 | Flixotide 25 mcgs/dose inhaler (GlaxoSmithKline UK Ltd) | Fluticasone propionate | 25 mcg/1 dose | 25 | 3020000 | 50 |
| 16305 | Flixotide 2mg/2ml Nebules (GlaxoSmithKline UK Ltd) | Fluticasone propionate | 1mg/1ml | 2000 | 3020000 | 4000 |
| 1426 | Flixotide 500 mcg Disc (Allen & Hanburys Ltd) | Fluticasone Propionate | 500 mcg | 500 | 3020000 | 1000 |
| 35374 | Flixotide 500 mcg disks (GlaxoSmithKline UK Ltd) | Fluticasone propionate | 500 mcg | 500 | 3020000 | 1000 |
| 35392 | Flixotide 500 mcg disks with Diskhaler (GlaxoSmithKline UK Ltd) | Fluticasone propionate | 500 mcg | 500 | 3020000 | 1000 |
| 43074 | Flixotide 500 mcgs/dose Accuhaler (GlaxoSmithKline UK Ltd) | Fluticasone propionate | 500 mcg/1 dose | 500 | 3020000 | 1000 |
| 56499 | Flixotide 500 mcgs/dose Accuhaler (Waymade Healthcare Plc) | Fluticasone propionate | 500 mcg/1 dose |  | 3020000 |  |
| 8635 | Flixotide 50 mcg Disc (Allen & Hanburys Ltd) | Fluticasone Propionate | 50 mcg | 50 | 3020000 | 100 |
| 35986 | Flixotide 50 mcg disks (GlaxoSmithKline UK Ltd) | Fluticasone propionate | 50 mcg | 50 | 3020000 | 100 |
| 36290 | Flixotide 50 mcg disks with Diskhaler (GlaxoSmithKline UK Ltd) | Fluticasone propionate | 50 mcg | 50 | 3020000 | 100 |
| 1518 | Flixotide 50 mcg/actuation Inhalation powder (Allen & Hanburys Ltd) | Fluticasone Propionate | 50 mcg/actuation | 50 | 3020000 | 100 |
| 57579 | Flixotide 50 mcgs/dose Accuhaler (DE Pharmaceuticals) | Fluticasone propionate | 50 mcg/1 dose |  | 3020000 |  |
| 42985 | Flixotide 50 mcgs/dose Accuhaler (GlaxoSmithKline UK Ltd) | Fluticasone propionate | 50 mcg/1 dose | 50 | 3020000 | 100 |
| 67253 | Flixotide 50 mcgs/dose Accuhaler (Mawdsley-Brooks & Company Ltd) | Fluticasone propionate | 50 mcg/1 dose |  | 3020000 |  |
| 56475 | Flixotide 50 mcgs/dose Accuhaler (Sigma Pharmaceuticals Plc) | Fluticasone propionate | 50 mcg/1 dose |  | 3020000 |  |
| 5309 | Flixotide 50 mcgs/dose Evohaler (GlaxoSmithKline UK Ltd) | Fluticasone propionate | 50 mcg/1 dose | 50 | 3020000 | 100 |
| 53057 | Flixotide 50 mcgs/dose Evohaler (Lexon (UK) Ltd) | Fluticasone propionate | 50 mcg/1 dose | 50 | 3020000 | 100 |
| 4926 | Flixotide accuhaler 100 100 mcg/inhalation Inhalation powder (Allen & Hanburys Ltd) | Fluticasone propionate | 100 mcg/1 dose | 100 | 3020000 | 200 |
| 911 | Flixotide accuhaler 250 250 mcg/inhalation Inhalation powder (Allen & Hanburys Ltd) | Fluticasone propionate | 250 mcg/1 dose | 250 | 3020000 | 500 |
| 5580 | Flixotide accuhaler 50 50 mcg/inhalation Inhalation powder (Allen & Hanburys Ltd) | Fluticasone propionate | 50 mcg/1 dose | 50 | 3020000 | 100 |
| 2440 | Flixotide accuhaler 500 500 mcg/inhalation Inhalation powder (Allen & Hanburys Ltd) | Fluticasone propionate | 500 mcg/1 dose | 500 | 3020000 | 1000 |
| 4131 | Fluticasone 100 mcg Disc | Fluticasone Propionate | 100 mcg | 100 | 3020000 | 200 |
| 35772 | Fluticasone 100 mcg inhalation powder blisters | Fluticasone propionate | 100 mcg | 100 | 3020000 | 200 |
| 35638 | Fluticasone 100 mcg inhalation powder blisters with device | Fluticasone propionate | 100 mcg | 100 | 3020000 | 200 |
| 5885 | Fluticasone 100 mcgs/dose dry powder inhaler | Fluticasone propionate | 100 mcg/1 dose | 100 | 3020000 | 200 |
| 4132 | Fluticasone 125 mcg/actuation Pressurised inhalation | Fluticasone Propionate | 125 mcg/actuation | 125 | 3020000 | 250 |
| 5975 | Fluticasone 125 mcgs/dose inhaler CFC free | Fluticasone propionate | 125 mcg/1 dose | 125 | 3020000 | 250 |
| 7638 | Fluticasone 250 mcg Disc | Fluticasone Propionate | 250 mcg | 250 | 3020000 | 500 |
| 35905 | Fluticasone 250 mcg inhalation powder blisters | Fluticasone propionate | 250 mcg | 250 | 3020000 | 500 |
| 36401 | Fluticasone 250 mcg inhalation powder blisters with device | Fluticasone propionate | 250 mcg | 250 | 3020000 | 500 |
| 2951 | Fluticasone 250 mcg/actuation Pressurised inhalation | Fluticasone Propionate | 250 mcg/actuation | 250 | 3020000 | 500 |
| 7948 | Fluticasone 250 mcgs/dose dry powder inhaler | Fluticasone propionate | 250 mcg/1 dose | 250 | 3020000 | 500 |
| 49772 | Fluticasone 250 mcgs/dose Evohaler (Sigma Pharmaceuticals Plc) | Fluticasone propionate | 250 mcg/1 dose | 250 | 3020000 | 500 |
| 5822 | Fluticasone 250 mcgs/dose inhaler CFC free | Fluticasone propionate | 250 mcg/1 dose | 250 | 3020000 | 500 |
| 2723 | Fluticasone 25 mcgs/dose inhaler | Fluticasone propionate | 25 mcg/1 dose | 25 | 3020000 | 50 |
| 11478 | Fluticasone 2mg/2ml nebuliser liquid unit dose vials | Fluticasone propionate | 1mg/1ml | 2000 | 3020000 | 4000 |
| 7891 | Fluticasone 500 mcg Disc | Fluticasone Propionate | 500 mcg | 500 | 3020000 | 1000 |
| 36462 | Fluticasone 500 mcg inhalation powder blisters | Fluticasone propionate | 500 mcg | 500 | 3020000 | 1000 |
| 35700 | Fluticasone 500 mcg inhalation powder blisters with device | Fluticasone propionate | 500 mcg | 500 | 3020000 | 1000 |
| 17465 | Fluticasone 500 mcgs/2ml nebuliser liquid unit dose vials | Fluticasone propionate | 250 mcg/1ml | 500 | 3020000 | 1000 |
| 2282 | Fluticasone 500 mcgs/dose dry powder inhaler | Fluticasone propionate | 500 mcg/1 dose | 500 | 3020000 | 1000 |
| 7602 | Fluticasone 50 mcg Disc | Fluticasone Propionate | 50 mcg | 50 | 3020000 | 100 |
| 37447 | Fluticasone 50 mcg inhalation powder blisters | Fluticasone propionate | 50 mcg | 50 | 3020000 | 100 |
| 36021 | Fluticasone 50 mcg inhalation powder blisters with device | Fluticasone propionate | 50 mcg | 50 | 3020000 | 100 |
| 4688 | Fluticasone 50 mcg/actuation Pressurised inhalation | Fluticasone Propionate | 50 mcg/actuation | 50 | 3020000 | 100 |
| 9164 | Fluticasone 50 mcgs/dose dry powder inhaler | Fluticasone propionate | 50 mcg/1 dose | 50 | 3020000 | 100 |
| 5223 | Fluticasone 50 mcgs/dose inhaler CFC free | Fluticasone propionate | 50 mcg/1 dose | 50 | 3020000 | 100 |
| 16018 | Mometasone 200 mcgs/dose dry powder inhaler | Mometasone furoate | 200 mcgs/1 dose | 200 | 3020000 | 400 |
| 10254 | Mometasone 400 mcgs/dose dry powder inhaler | Mometasone furoate | 400 mcgs/1 dose | 400 | 3020000 | 800 |
| 23741 | Novolizer budesonide 200 mcg/actuation Pressurised inhalation (Meda Pharmaceuticals Ltd) | Budesonide | 200 mcg/actuation | 200 | 3020000 | 200 |
| 1959 | Pulmicort 0.5mg Respules (AstraZeneca UK Ltd) | Budesonide | 2.5 mcg/1ml | 500 | 3020000 | 500 |
| 52732 | Pulmicort 0.5mg Respules (Necessity Supplies Ltd) | Budesonide | 2.5 mcg/1ml | 500 | 3020000 | 500 |
| 50037 | Pulmicort 0.5mg Respules (Waymade Healthcare Plc) | Budesonide | 2.5 mcg/1ml | 500 | 3020000 | 500 |
| 960 | Pulmicort 100 Turbohaler (AstraZeneca UK Ltd) | Budesonide | 100 mcg/1 dose | 100 | 3020000 | 100 |
| 67322 | Pulmicort 100 Turbohaler (Waymade Healthcare Plc) | Budesonide | 100 mcg/1 dose |  | 3020000 |  |
| 39099 | Pulmicort 100 mcgs/dose inhaler CFC free (AstraZeneca UK Ltd) | Budesonide | 100 mcg/1 dose | 100 | 0 | 100 |
| 1956 | Pulmicort 1mg Respules (AstraZeneca UK Ltd) | Budesonide | 0.5 mcg/1ml | 1000 | 3020000 | 1000 |
| 67261 | Pulmicort 1mg Respules (Sigma Pharmaceuticals Plc) | Budesonide | 500 mcg/1ml |  | 3020000 |  |
| 956 | Pulmicort 200 Turbohaler (AstraZeneca UK Ltd) | Budesonide | 200 mcg/1 dose | 200 | 3020000 | 200 |
| 60937 | Pulmicort 200 Turbohaler (Dowelhurst Ltd) | Budesonide | 200 mcg/1 dose |  | 3020000 |  |
| 56498 | Pulmicort 200 Turbohaler (Waymade Healthcare Plc) | Budesonide | 200 mcg/1 dose |  | 3020000 |  |
| 454 | Pulmicort 200 mcg Inhaler (AstraZeneca UK Ltd) | Budesonide | 200 mcg/1 dose | 200 | 3020000 | 200 |
| 2125 | Pulmicort 200 mcg Refill canister (AstraZeneca UK Ltd) | Budesonide | 200 mcg | 200 | 3020000 | 200 |
| 49711 | Pulmicort 200 mcgs/dose inhaler (AstraZeneca UK Ltd) | Budesonide | 200 mcg/1 dose | 200 | 3020000 | 200 |
| 40057 | Pulmicort 200 mcgs/dose inhaler CFC free (AstraZeneca UK Ltd) | Budesonide | 200 mcg/1 dose | 200 | 3020000 | 200 |
| 908 | Pulmicort 400 Turbohaler (AstraZeneca UK Ltd) | Budesonide | 400 mcg/1 dose | 400 | 3020000 | 400 |
| 67239 | Pulmicort 400 Turbohaler (Waymade Healthcare Plc) | Budesonide | 400 mcg/1 dose |  | 3020000 |  |
| 4545 | Pulmicort LS 50 mcg Refill canister (AstraZeneca UK Ltd) | Budesonide | 50 mcg | 50 | 3020000 | 50 |
| 1680 | Pulmicort LS 50 mcgs/dose inhaler (AstraZeneca UK Ltd) | Budesonide | 50 mcg/1 dose | 50 | 3020000 | 50 |
| 14757 | Pulvinal Beclometasone Dipropionate 100 mcgs/dose dry powder inhaler (Chiesi Ltd) | Beclometasone dipropionate | 100 mcg/1 dose | 100 | 3020000 | 100 |
| 13037 | Pulvinal Beclometasone Dipropionate 200 mcgs/dose dry powder inhaler (Chiesi Ltd) | Beclometasone dipropionate | 200 mcg/1 dose | 200 | 3020000 | 200 |
| 14736 | Pulvinal Beclometasone Dipropionate 400 mcgs/dose dry powder inhaler (Chiesi Ltd) | Beclometasone dipropionate | 400 mcg/1 dose | 400 | 3020000 | 400 |
| 51480 | Qvar 100 Autohaler (Doncaster Pharmaceuticals Ltd) | Beclometasone dipropionate | 100 mcg/1 dose | 100 | 3020000 | 200 |
| 52806 | Qvar 100 Autohaler (Lexon (UK) Ltd) | Beclometasone dipropionate | 100 mcg/1 dose | 100 | 3020000 | 200 |
| 54399 | Qvar 100 Autohaler (Sigma Pharmaceuticals Plc) | Beclometasone dipropionate | 100 mcg/1 dose | 100 | 3020000 | 200 |
| 53480 | Qvar 100 Autohaler (Stephar (U.K.) Ltd) | Beclometasone dipropionate | 100 mcg/1 dose | 100 | 3020000 | 200 |
| 4413 | Qvar 100 Autohaler (Teva UK Ltd) | Beclometasone dipropionate | 100 mcg/1 dose | 100 | 3020000 | 200 |
| 50287 | Qvar 100 inhaler (Doncaster Pharmaceuticals Ltd) | Beclometasone dipropionate | 100 mcg/1 dose | 100 | 3020000 | 200 |
| 51681 | Qvar 100 inhaler (Sigma Pharmaceuticals Plc) | Beclometasone dipropionate | 100 mcg/1 dose | 100 | 3020000 | 200 |
| 2335 | Qvar 100 inhaler (Teva UK Ltd) | Beclometasone dipropionate | 100 mcg/1 dose | 100 | 3020000 | 200 |
| 51234 | Qvar 100 inhaler (Waymade Healthcare Plc) | Beclometasone dipropionate | 100 mcg/1 dose | 100 | 3020000 | 200 |
| 50129 | Qvar 100 mcgs/dose Easi-Breathe inhaler (Doncaster Pharmaceuticals Ltd) | Beclometasone dipropionate | 100 mcg/1 dose | 100 | 3020000 | 200 |
| 48709 | Qvar 100 mcgs/dose Easi-Breathe inhaler (Sigma Pharmaceuticals Plc) | Beclometasone dipropionate | 100 mcg/1 dose | 100 | 3020000 | 200 |
| 18848 | Qvar 100 mcgs/dose Easi-Breathe inhaler (Teva UK Ltd) | Beclometasone dipropionate | 100 mcg/1 dose | 100 | 3020000 | 200 |
| 3220 | Qvar 50 Autohaler (Teva UK Ltd) | Beclometasone dipropionate | 50 mcg/1 dose | 50 | 3020000 | 100 |
| 54207 | Qvar 50 inhaler (Doncaster Pharmaceuticals Ltd) | Beclometasone dipropionate | 50 mcg/1 dose | 50 | 3020000 | 100 |
| 51415 | Qvar 50 inhaler (Mawdsley-Brooks & Company Ltd) | Beclometasone dipropionate | 50 mcg/1 dose | 50 | 3020000 | 100 |
| 3546 | Qvar 50 inhaler (Teva UK Ltd) | Beclometasone dipropionate | 50 mcg/1 dose | 50 | 3020000 | 100 |
| 56493 | Qvar 50 mcgs/dose Easi-Breathe inhaler (Sigma Pharmaceuticals Plc) | Beclometasone dipropionate | 50 mcg/1 dose |  | 3020000 |  |
| 14294 | Qvar 50 mcgs/dose Easi-Breathe inhaler (Teva UK Ltd) | Beclometasone dipropionate | 50 mcg/1 dose | 50 | 3020000 | 100 |
| 24898 | Spacehaler BDP 100 mcg/actuation Spacehaler (Celltech Pharma Europe Ltd) | Beclometasone Dipropionate | 100 mcg/actuation | 100 | 3020000 | 100 |
| 20825 | Spacehaler BDP 250 mcg/actuation Spacehaler (Celltech Pharma Europe Ltd) | Beclometasone Dipropionate | 250 mcg/actuation | 250 | 3020000 | 250 |
| 28761 | Spacehaler BDP 50 mcg/actuation Spacehaler (Celltech Pharma Europe Ltd) | Beclometasone Dipropionate | 50 mcg/actuation | 50 | 3020000 | 50 |

Supplementary Table 30: Theophylline product codes

| **Product code** | **Product name** | **Drug substance name** | **Substance strength** | **BNF code** |
| --- | --- | --- | --- | --- |
| 218 | AMINOPHYLLINE 100 MG CAP | Aminophylline |  | 3010300 |
| 8057 | Aminophylline 100mg modified-release tablets | Aminophylline Hydrate | 100mg | 3010300 |
| 8056 | Aminophylline 100mg tablets | Aminophylline | 100mg | 0 |
| 27842 | AMINOPHYLLINE 2 ML INJ | Aminophylline |  | 3010300 |
| 22080 | AMINOPHYLLINE 20 ML INJ | Aminophylline |  | 3010300 |
| 17140 | Aminophylline 200mg tablets | Aminophylline | 200mg | 3010300 |
| 30596 | Aminophylline 225mg Modified-release tablet (Actavis UK Ltd) | Aminophylline | 225mg | 3010300 |
| 29273 | Aminophylline 225mg Modified-release tablet (Hillcross Pharmaceuticals Ltd) | Aminophylline | 225mg | 3010300 |
| 555 | Aminophylline 225mg modified-release tablets | Aminophylline | 225mg | 0 |
| 14991 | Aminophylline 250mg/10ml injection | Aminophylline | 250mg/10ml | 3010300 |
| 50018 | Aminophylline 250mg/10ml solution for injection ampoules | Aminophylline | 25mg/1ml | 3010300 |
| 57228 | Aminophylline 250mg/10ml solution for injection ampoules (A A H Pharmaceuticals Ltd) | Aminophylline | 25mg/1ml | 3010300 |
| 58600 | Aminophylline 250mg/10ml solution for injection ampoules (AMCo) | Aminophylline | 25mg/1ml | 3010300 |
| 42910 | Aminophylline 250mg/10ml solution for injection ampoules (Martindale Pharmaceuticals Ltd) | Aminophylline | 25mg/1ml | 3010300 |
| 28241 | Aminophylline 250mg/10ml solution for injection Minijet pre-filled syringes (UCB Pharma Ltd) | Aminophylline | 25mg/1ml | 0 |
| 10561 | Aminophylline 250mg/ml injection | Aminophylline | 250mg/ml | 3010300 |
| 42511 | Aminophylline 25mg/ml Injection (Celltech Pharma Europe Ltd) | Aminophylline | 25mg/ml | 3010300 |
| 4514 | Aminophylline 350mg modified-release tablets | Aminophylline Hydrate | 350mg | 3010300 |
| 25125 | Aminophylline 360mg suppositories | Aminophylline | 360mg | 3010300 |
| 61346 | Aminophylline 360mg suppositories (Special Order) |  |  | 3010300 |
| 20225 | AMINOPHYLLINE 500 MG INJ | Aminophylline |  | 3010300 |
| 6988 | Aminophylline hydrate 100mg modified-release tablets | Aminophylline Hydrate | 100mg | 0 |
| 17002 | Aminophylline hydrate 225mg modified-release tablets | Aminophylline hydrate | 225mg | 3010300 |
| 16994 | Aminophylline hydrate 350mg modified-release tablets | Aminophylline hydrate | 350mg | 3010300 |
| 25937 | AMINOPHYLLINE INTRAMUSCULAR 500 MG INJ | Aminophylline |  | 3010300 |
| 23572 | Aminophylline sr 225mg Modified-release tablet (IVAX Pharmaceuticals UK Ltd) | Aminophylline | 225mg | 3010300 |
| 13529 | Amnivent-225 SR tablets (Ashbourne Pharmaceuticals Ltd) | Aminophylline | 225mg | 0 |
| 10831 | Biophylline 125mg/5ml Oral solution (Lorex Synthelabo Ltd) | Theophylline Sodium Glycinate | 125mg/5ml | 3010300 |
| 24418 | Biophylline 350mg Tablet (Lorex Synthelabo Ltd) | Theophylline | 350mg | 3010300 |
| 24674 | Biophylline 500mg Tablet (Lorex Synthelabo Ltd) | Theophylline | 500mg | 3010300 |
| 4591 | Choledyl 100mg Tablet (Parke-davis Research Laboratories) | Choline Hydrogen Tartrate | 100mg | 3010300 |
| 4592 | Choledyl 200mg Tablet (Parke-davis Research Laboratories) | Choline Hydrogen Tartrate | 200mg | 3010300 |
| 3187 | Choledyl 62.5mg/5ml Oral solution (Parke-davis Research Laboratories) | Choline Hydrogen Tartrate | 62.5mg/5ml | 3010300 |
| 18288 | Choline theophyllinate 100mg tablets | Choline Hydrogen Tartrate | 100mg | 3010300 |
| 7832 | Choline theophyllinate 200mg tablets | Choline Hydrogen Tartrate | 200mg | 3010300 |
| 15561 | EPHEDRINE 11MG/THEOPHYLLINE 120MG 11 MG TAB | Theophylline/Ephedrine hydrochloride |  | 3010300 |
| 24035 | EPHEDRINE 15MG/THEOPHYLLINE 120MG 15 MG TAB | Theophylline/Ephedrine hydrochloride |  | 3010300 |
| 8705 | EPHEDRINE HCL 24MG/THEOPHYLLINE 120MG MG TAB | Theophylline/Ephedrine hydrochloride |  | 3010300 |
| 28786 | EPHEDRINE HCL 25MG/AMINOPHYLLINE 130MG MG CAP | Theophylline/Ephedrine hydrochloride |  | 3010300 |
| 26724 | EPHEDRINE HCL/AMINOPHYLLINE E/C TAB | Theophylline/Ephedrine hydrochloride |  | 3010300 |
| 7477 | Franol Plus tablets (Sanofi) | Theophylline/Ephedrine sulfate | 120mg + 15mg | 0 |
| 2609 | Franol tablets (Sanofi) | Theophylline/Ephedrine hydrochloride | 120mg + 11mg | 0 |
| 21769 | Lasma 300mg Tablet (Pharmax Ltd) | Theophylline | 300mg | 3010300 |
| 14739 | Norphyllin SR 225mg tablets (Teva UK Ltd) | Aminophylline | 225mg | 0 |
| 7841 | Nuelin 125mg tablets (3M Health Care Ltd) | Theophylline | 125mg | 0 |
| 10331 | Nuelin 60mg/5ml liquid (3M Health Care Ltd) | Theophylline sodium glycinate | 12mg/1ml | 3010300 |
| 2995 | Nuelin SA 175mg tablets (Meda Pharmaceuticals Ltd) | Theophylline | 175mg | 3010300 |
| 5261 | Nuelin SA 250 tablets (Meda Pharmaceuticals Ltd) | Theophylline | 250mg | 3010300 |
| 12699 | Pecram 225mg Modified-release tablet (Novartis Consumer Health UK Ltd) | Aminophylline | 225mg | 3010300 |
| 590 | Phyllocontin Continus 225mg tablets (Napp Pharmaceuticals Ltd) | Aminophylline hydrate | 225mg | 3010300 |
| 8806 | Phyllocontin continus 350mg Tablet (Napp Pharmaceuticals Ltd) | Aminophylline hydrate | 350mg | 3010300 |
| 39040 | Phyllocontin Forte Continus 350mg tablets (Napp Pharmaceuticals Ltd) | Aminophylline hydrate | 350mg | 3010300 |
| 10407 | Phyllocontin Paediatric Continus 100mg tablets (Napp Pharmaceuticals Ltd) | Aminophylline hydrate | 100mg | 0 |
| 11993 | Pro-vent 300mg Capsule (Wellcome Medical Division) | Theophylline | 300mg | 3010300 |
| 863 | Slo-phyllin 125mg Capsule (Lipha Pharmaceuticals Ltd) | Theophylline | 125mg | 3010300 |
| 15284 | Slo-Phyllin 125mg capsules (Merck Serono Ltd) | Theophylline | 125mg | 3010300 |
| 2757 | Slo-phyllin 250mg Capsule (Lipha Pharmaceuticals Ltd) | Theophylline | 250mg | 3010300 |
| 6315 | Slo-Phyllin 250mg capsules (Merck Serono Ltd) | Theophylline | 250mg | 3010300 |
| 1097 | Slo-phyllin 60mg Capsule (Lipha Pharmaceuticals Ltd) | Theophylline | 60mg | 3010300 |
| 11719 | Slo-Phyllin 60mg capsules (Merck Serono Ltd) | Theophylline | 60mg | 3010300 |
| 27944 | Tedral Oral solution (Parke-davis Research Laboratories) | Theophylline/Ephedrine Hydrochloride |  | 3010400 |
| 12274 | Tedral Tablet (Parke-davis Research Laboratories) | Theophylline/Ephedrine Hydrochloride |  | 3010400 |
| 24023 | Theodrox Tablet (3M Health Care Ltd) | Aluminium Hydroxide/Aminophylline |  | 3010300 |
| 7731 | Theo-Dur 200mg tablets (AstraZeneca UK Ltd) | Theophylline | 200mg | 3010300 |
| 7730 | Theo-Dur 300mg tablets (AstraZeneca UK Ltd) | Theophylline | 300mg | 3010300 |
| 1832 | Theograd 350mg Tablet (Abbott Laboratories Ltd) | Theophylline | 350mg | 3010300 |
| 8955 | THEOPHYLLINE 100 MG TAB | Theophylline | 100mg | 3010300 |
| 15365 | Theophylline 10mg/5ml SF elixir | Theophylline Sodium Glycinate | 10mg/5ml | 3010300 |
| 15153 | Theophylline 120mg / Ephedrine hydrochloride 11mg tablets | Theophylline/Ephedrine hydrochloride | 120mg + 11mg | 0 |
| 26860 | Theophylline 120mg / Ephedrine sulfate 15mg tablets | Theophylline/Ephedrine sulfate | 120mg + 15mg | 0 |
| 879 | Theophylline 125mg modified-release capsules | Theophylline | 125mg | 3010300 |
| 4593 | Theophylline 125mg tablets | Theophylline | 125mg | 0 |
| 10723 | Theophylline 125mg/5ml syrup | Theophylline Sodium Glycinate | 125mg/5ml | 3010300 |
| 3388 | Theophylline 175mg modified-release tablets | Theophylline | 175mg | 3010300 |
| 273 | THEOPHYLLINE 200 MG CAP | Theophylline |  | 3010300 |
| 1833 | Theophylline 200mg modified-release tablets | Theophylline | 200mg | 3010300 |
| 2147 | Theophylline 250mg modified-release capsules | Theophylline | 250mg | 3010300 |
| 7733 | Theophylline 250mg modified-release tablets | Theophylline | 250mg | 3010300 |
| 48484 | Theophylline 250mg/5ml oral suspension | Theophylline | 50mg/1ml | 3010300 |
| 15409 | THEOPHYLLINE 3 MG SOL | Theophylline |  | 3010300 |
| 12240 | Theophylline 300mg modified release capsules | Theophylline | 300mg | 3010300 |
| 7732 | Theophylline 300mg modified-release tablets | Theophylline | 300mg | 3010300 |
| 9092 | Theophylline 350mg modified release tablets | Theophylline | 350mg | 3010300 |
| 1834 | Theophylline 400mg modified-release tablets | Theophylline | 400mg | 3010300 |
| 38120 | Theophylline 500mg modified release tablets | Theophylline | 500mg | 3010300 |
| 880 | Theophylline 60mg modified-release capsules | Theophylline | 60mg | 3010300 |
| 10433 | Theophylline 60mg/5ml oral solution | Theophylline sodium glycinate | 12mg/1ml | 3010300 |
| 51430 | Theophylline 60mg/5ml oral suspension | Theophylline |  | 3010300 |
| 10744 | THEOPHYLLINE 80 MG ELI | Theophylline |  | 3010300 |
| 25093 | THEOPHYLLINE S/R | Theophylline |  | 3010300 |
| 1423 | Uniphyllin Continus 200mg tablets (Napp Pharmaceuticals Ltd) | Theophylline | 200mg | 3010300 |
| 5941 | Uniphyllin Continus 300mg tablets (Napp Pharmaceuticals Ltd) | Theophylline | 300mg | 3010300 |
| 5453 | Uniphyllin Continus 400mg tablets (Napp Pharmaceuticals Ltd) | Theophylline | 400mg | 3010300 |

Supplementary Table 31: LTRA product codes

| **Product code** | **Product name** | **Drug substance name** | **Substance strength** | **Formulation** | **Route of administration** | **BNF code** | **BNF header** |
| --- | --- | --- | --- | --- | --- | --- | --- |
| 622 | Montelukast 4mg chewable tablets sugar free | Montelukast sodium | 4mg | Chewable tablet | Oral | 3030200 | Leukotriene Receptor Antagonists |
| 695 | Singulair 10mg tablets (Merck Sharp & Dohme Ltd) | Montelukast sodium | 10mg | Tablet | Oral | 3030200 | Leukotriene Receptor Antagonists |
| 808 | Montelukast 10mg tablets | Montelukast sodium | 10mg | Tablet | Oral | 3030200 | Leukotriene Receptor Antagonists |
| 1973 | Accolate 20mg tablers (AstraZeneca Uk Ltd) | Zafirlukast | 20mg | Tablet | Oral | 3030200 | Leukotriene Receptor Antagonists |
| 5594 | Singulair Paediatric 5mg chewable tablets (Merck Sharp & Dohme Ltd) | Montelukast sodium | 5mg | Chewable tablet | Oral | 3030200 | Leukotriene Receptor Antagonists |
| 5957 | Montelukast 5mg chewable tablets sugar free | Montelukast sodium | 5mg | Chewable tablet | Oral | 3030200 | Leukotriene Receptor Antagonists |
| 7088 | Montelukast 4mg granules sachets sugar free | Montelukast sodium | 4mg | Granules | Oral | 3030200 | Leukotriene Receptor Antagonists |
| 7132 | Zafirlukast 20mg tablets | Zafirlukast | 20mg | Tablet | Oral | 3030200 | Leukotriene Receptor Antagonists |
| 14162 | Singulair Paediatric 4mg chewable tablets (Merck Sharp & Dohme Ltd) | Montelukast sodium | 4mg | Chewable tablet | Oral | 3030200 | Leukotriene Receptor Antagonists |
| 14200 | Singulair Paediatric 4mg granules sachets (Merck Sharp & Dohme Ltd) | Montelukast sodium | 4mg | Granules | Oral | 3030200 | Leukotriene Receptor Antagonists |
| 17701 | Zafirlukast | Zafirlukast |  |  | Oral | 3030200 | Leukotriene Receptor Antagonists |
| 48396 | Singulair 10mg tablets (Necessity Supplies Ltd) | Montelukast sodium | 10mg | Tablet | Oral | 3030200 | Leukotriene Receptor Antagonists |
| 56604 | Montelukast 4mg chewable tablets sugar free (Actavis UK Ltd) | Montelukast sodium | 4mg | Chewable tablet | Oral | 3030200 | Leukotriene Receptor Antagonists |
| 56756 | Montelukast 4mg granules sachets sugar free (A A H Pharmaceuticals Ltd) | Montelukast sodium | 4mg | Granules | Oral | 3030200 | Leukotriene Receptor Antagonists |
| 57621 | Singulair Paediatric 4mg granules sachets (Mawdsley-Brooks & Company Ltd) | Montelukast sodium | 4mg | Granules | Oral | 3030200 | Leukotriene Receptor Antagonists |
| 59263 | Montelukast 10mg tablets (Teva UK Ltd) | Montelukast sodium | 10mg | Tablet | Oral | 3030200 | Leukotriene Receptor Antagonists |
| 59819 | Montelukast 10mg tablets (Actavis UK Ltd) | Montelukast sodium | 10mg | Tablet | Oral | 3030200 | Leukotriene Receptor Antagonists |
| 59968 | Montelukast 5mg chewable tablets sugar free (Teva UK Ltd) | Montelukast sodium | 5mg | Chewable tablet | Oral | 3030200 | Leukotriene Receptor Antagonists |
| 60331 | Montelukast 10mg tablets (Ranbaxy (UK) Ltd) | Montelukast sodium | 10mg | Tablet | Oral | 3030200 | Leukotriene Receptor Antagonists |
| 62410 | Montelukast 10mg tablets (Alliance Healthcare (Distribution) Ltd) | Montelukast sodium | 10mg | Tablet | Oral | 3030200 | Leukotriene Receptor Antagonists |
| 62490 | Montelukast 10mg tablets (A A H Pharmaceuticals Ltd) | Montelukast sodium | 10mg | Tablet | Oral | 3030200 | Leukotriene Receptor Antagonists |
| 63457 | Montelukast 5mg chewable tablets sugar free (Accord Healthcare Ltd) | Montelukast sodium | 5mg | Chewable tablet | Oral | 3030200 | Leukotriene Receptor Antagonists |
| 64444 | Singulair 10mg tablets (DE Pharmaceuticals) | Montelukast sodium | 10mg | Tablet | Oral | 3030200 | Leukotriene Receptor Antagonists |
| 64648 | Montelukast 10mg tablets (Milpharm Ltd) | Montelukast sodium | 10mg | Tablet | Oral | 3030200 | Leukotriene Receptor Antagonists |
| 65038 | Montelukast 10mg tablets (Accord Healthcare Ltd) | Montelukast sodium | 10mg | Tablet | Oral | 3030200 | Leukotriene Receptor Antagonists |
| 66410 | Montelukast 4mg granules sachets sugar free (Teva UK Ltd) | Montelukast sodium | 4mg | Granules | Oral | 3030200 | Leukotriene Receptor Antagonists |
| 67561 | Montelukast 4mg chewable tablets sugar free (Accord Healthcare Ltd) | Montelukast sodium | 4mg | Chewable tablet | Oral | 3030200 | Leukotriene Receptor Antagonists |
| 68212 | Montelukast 4mg chewable tablets sugar free (Alliance Healthcare (Distribution) Ltd) | Montelukast sodium | 4mg | Chewable tablet | Oral | 3030200 | Leukotriene Receptor Antagonists |
| 68366 | Montelukast 5mg chewable tablets sugar free (Actavis UK Ltd) | Montelukast sodium | 5mg | Chewable tablet | Oral | 3030200 | Leukotriene Receptor Antagonists |
| 68498 | Montelukast 10mg tablets (Dr Reddy's Laboratories (UK) Ltd) | Montelukast sodium | 10mg | Tablet | Oral | 3030200 | Leukotriene Receptor Antagonists |

Supplementary Table 32: Chromones product codes

| **Product code** | **Product name** | **Drug substance name** | **Substance strength** | **BNF code** |
| --- | --- | --- | --- | --- |
| 314 | Intal 5mg/dose inhaler (Aventis Pharma) | Sodium cromoglicate | 5mg/1dose | 0 |
| 964 | Sodium cromoglicate 5mg/dose inhaler | Sodium cromoglicate | 5mg/1dose | 0 |
| 1419 | Rynacrom Capsule (Rpr / Fisons) | Sodium Cromoglicate |  | 12020100 |
| 1422 | Cromogen 5mg/dose inhaler (Teva UK Ltd) | Sodium cromoglicate | 5mg/1dose | 0 |
| 1629 | Intal 20mg/2ml nebuliser solution unit dose vials (Aventis Pharma) | Sodium cromoglicate | 10mg/1ml | 3030100 |
| 1683 | Intal 20mg Spincaps (Sanofi) | Sodium cromoglicate | 20mg | 0 |
| 1728 | Cromogen 5mg/dose Easi-Breathe inhaler (Teva UK Ltd) | Sodium cromoglicate | 5mg/1dose | 0 |
| 2158 | Sodium cromoglicate 5mg/dose breath actuated inhaler | Sodium cromoglicate | 5mg/1dose | 0 |
| 2911 | Sodium cromoglicate 20mg inhalation powder capsules | Sodium cromoglicate | 20mg | 0 |
| 3585 | Steri-neb cromogen 10mg/ml Nebuliser liquid (IVAX Pharmaceuticals UK Ltd) | Sodium cromoglicate | 10mg/1ml | 3030100 |
| 3688 | Tilade 2mg/dose inhaler (Sanofi) | Nedocromil sodium | 2mg/1dose | 0 |
| 4100 | Intal autohaler 5 5mg/inhalation Pressurised inhalation (Aventis Pharma) | Sodium cromoglicate | 5mg/1dose | 3030100 |
| 4647 | Intal 5mg/dose Syncroner with spacer (Aventis Pharma) | Sodium cromoglicate | 5mg/1dose | 0 |
| 7594 | Sodium cromoglicate inhalation capsules | Sodium Cromoglicate |  | 12020100 |
| 7929 | Sodium cromoglicate 100mg capsules | Sodium cromoglicate | 100mg | 1050400 |
| 7972 | Intal 5mg/dose Fisonair with spacer (Sanofi) | Sodium cromoglicate | 5mg/1dose | 0 |
| 8215 | Tilade 2mg/inhalation Inhalation powder (Sanofi) | Nedocromil Sodium | 2mg/inhalation | 3030100 |
| 8498 | Sodium cromoglicate 20mg/2ml nebuliser liquid unit dose vials | Sodium cromoglicate | 10mg/1ml | 3030100 |
| 8608 | Nedocromil sodium 2mg/inhalation inhaler | Nedocromil Sodium | 2mg/inhalation | 3030100 |
| 10483 | Nalcrom 100mg capsules (Sanofi) | Sodium cromoglicate | 100mg | 1050400 |
| 10597 | Tilade 2mg/dose Syncroner with spacer (Sanofi) | Nedocromil sodium | 2mg/1dose | 0 |
| 13256 | Nedocromil 2mg/dose inhaler CFC free | Nedocromil Sodium | 2mg/inhalation | 3030100 |
| 14603 | Sodium cromoglicate 5mg/dose inhaler with spacer | Sodium cromoglicate | 5mg/1dose | 0 |
| 15765 | Sodium cromoglicate 5mg/inhalation inhaler & spacer | Sodium Cromoglicate | 5mg/inhalation | 3030100 |
| 25119 | Tilade 2mg/dose inhaler CFC free (Sanofi) | Nedocromil Sodium | 2mg/inhalation | 3030100 |
| 37615 | Sodium cromoglicate 1mg/inhalation inhaler | Sodium Cromoglicate | 1mg/inhalation | 3030100 |
| 38471 | Sodium cromoglicate 5mg/dose inhaler CFC free | Sodium cromoglicate | 5mg/1dose | 3030100 |
| 38501 | Intal 5mg/dose inhaler CFC free (Sanofi) | Sodium cromoglicate | 5mg/1dose | 3030100 |
| 56585 | Sodium cromoglicate 100mg/5ml oral solution | Sodium cromoglicate | 20mg/1ml | 01050400/11040200/12020100/12020151 |
| 59898 | Nedocromil 2mg/dose inhaler | Nedocromil sodium | 2mg/1dose | 0 |
| 63644 | Nedocromil 2mg/dose inhaler with spacer | Nedocromil sodium | 2mg/1dose | 0 |
| 2610 | Intal compound Capsule (Rhone-Poulenc Rorer Ltd) | Isoprenaline Hydrochloride/Sodium Cromoglicate |  | 03010102/03030100 |

Supplementary Table 33: Combination chromones/beta-2 agonist product codes

| **Product code** | **Product name** | **Drug substance name** | **Substance strength** | **BNF code** |
| --- | --- | --- | --- | --- |
| 24380 | Sodium cromoglicate 1mg/dose / Salbutamol 100micrograms/dose inhaler with spacer | Sodium cromoglicate/Salbutamol sulfate | 1mg/1dose + 100microgram/1dose | 0 |
| 8267 | Sodium cromoglicate 1mg/dose / Salbutamol 100micrograms/dose inhaler | Salbutamol sulfate/Sodium cromoglicate | 100microgram/1dose + 1mg/1dose | 0 |
| 10360 | Aerocrom inhaler (Castlemead Healthcare Ltd) | Salbutamol sulfate/Sodium cromoglicate | 100microgram/1dose + 1mg/1dose | 0 |
| 18314 | Aerocrom Syncroner with spacer (Castlemead Healthcare Ltd) | Sodium cromoglicate/Salbutamol sulfate | 1mg/1dose + 100microgram/1dose | 0 |
| 24380 | Sodium cromoglicate 1mg/dose / Salbutamol 100micrograms/dose inhaler with spacer | Sodium cromoglicate/Salbutamol sulfate | 1mg/1dose + 100microgram/1dose | 0 |
| 54641 | Isoprenaline sulphate with sodium cromoglicate inhalation Capsule | Isoprenaline Hydrochloride/Sodium Cromoglicate |  | 03010102/03030100 |
| 20180 | Sodium cromoglicate with isoprenaline inhalation capsules | Isoprenaline Hydrochloride/Sodium Cromoglicate |  | 03010102/03030100 |

Supplementary Table 34: Oral corticosteroids product codes

| **Product code** | **Product name** | **Drug substance name** | **Substance strength** | **BNF code** |
| --- | --- | --- | --- | --- |
| 186 | Dexamethasone 500micrograms/5ml oral solution | Dexamethasone | 100microgram/1ml | 03010000/04065300/06030200 |
| 1280 | Dexamethasone 2mg tablets | Dexamethasone | 2mg | 06030200/64090000 |
| 4779 | Dexamethasone 500microgram tablets | Dexamethasone | 500microgram | 06030200/64090000 |
| 5157 | Dexamethasone 2mg/5ml oral solution | Dexamethasone | 2mg/5ml | 6030200 |
| 9994 | Decadron 500microgram tablets (Merck Sharp & Dohme Ltd) | Dexamethasone | 500microgram | 06030200/64090000 |
| 21903 | Oradexon-organon 2mg Tablet (Organon Laboratories Ltd) | Dexamethasone | 2mg | 6030200 |
| 34801 | Dexamethasone 0.5mg/5ml Oral solution (Rosemont Pharmaceuticals Ltd) | Dexamethasone | 100microgram/1ml | 6030200 |
| 34880 | Dexamethasone 2mg tablets (Aspen Pharma Trading Ltd) | Dexamethasone | 2mg | 06030200/64090000 |
| 34915 | Dexamethasone 500microgram tablets (Organon Laboratories Ltd) | Dexamethasone | 500microgram | 06030200/64090000 |
| 36055 | Dexamethasone 2mg Tablet (Hillcross Pharmaceuticals Ltd) | Dexamethasone | 2mg | 6030200 |
| 45234 | Dexamethasone 100microgram capsules | Dexamethasone | 100microgram | 03010000/04065300/06030200 |
| 52396 | Dexamethasone 1mg/5ml oral solution | Dexamethasone | 200microgram/1ml | 03010000/04065300/06030200 |
| 53207 | Dexamethasone tablets | Dexamethasone |  | 6030200 |
| 54793 | Dexamethasone 2mg/5ml oral suspension | Dexamethasone | 400microgram/1ml | 03010000/04065300/06030200 |
| 55401 | Dexamethasone 500microgram tablets (A A H Pharmaceuticals Ltd) | Dexamethasone | 500microgram | 06030200/64090000 |
| 56347 | Dexamethasone 5mg/5ml oral solution | Dexamethasone | 1mg/1ml | 03010000/04065300/06030200 |
| 60120 | Dexamethasone 2mg tablets (Alliance Healthcare (Distribution) Ltd) | Dexamethasone | 2mg | 06030200/64090000 |
| 62909 | Dexamethasone 2mg tablets (A A H Pharmaceuticals Ltd) | Dexamethasone | 2mg | 06030200/64090000 |
| 68182 | Dexamethasone 2mg tablets (Teva UK Ltd) | Dexamethasone | 2mg | 06030200/64090000 |
| 68489 | Dexamethasone 4mg tablets | Dexamethasone | 4mg | 6030200 |
| 68593 | Dexamethasone 5mg/5ml oral suspension | Dexamethasone | 1mg/1ml | 03010000/04065300/06030200 |
| 4943 | Dexamethasone 2mg/5ml oral solution sugar free | Dexamethasone sodium phosphate | 400microgram/1ml | 03010000/04065300/06030200 |
| 21218 | Dexsol 2mg/5ml oral solution (Rosemont Pharmaceuticals Ltd) | Dexamethasone sodium phosphate | 400microgram/1ml | 03010000/04065300/06030200 |
| 21668 | Decadron shock pak 20 20mg/ml Shock pack (Merck Sharp & Dohme Ltd) | Dexamethasone Sodium Phosphate | 20mg/ml | 6030200 |
| 26300 | Dexamethasone 20mg/ml shock pack | Dexamethasone Sodium Phosphate | 20mg/ml | 6030200 |
| 58474 | Dexamethasone 2mg/5ml oral solution sugar free (A A H Pharmaceuticals Ltd) | Dexamethasone sodium phosphate | 400microgram/1ml | 03010000/04065300/06030200 |
| 64050 | Martapan 2mg/5ml oral solution (Martindale Pharmaceuticals Ltd) | Dexamethasone sodium phosphate | 400microgram/1ml | 03010000/04065300/06030200 |
| 64766 | Dexamethasone 20mg/5ml oral solution sugar free | Dexamethasone sodium phosphate | 4mg/1ml | 03010000/04065300/06030200 |
| 66524 | Dexamethasone 4mg soluble tablets sugar free | Dexamethasone sodium phosphate | 4mg | 06030200/64090000 |
| 68860 | Dexamethasone 8mg soluble tablets sugar free (A A H Pharmaceuticals Ltd) | Dexamethasone sodium phosphate | 8mg | 06030200/64090000 |
| 44 | Prednisolone 5mg gastro-resistant tablets | Prednisolone | 5mg | 01050200/03020000/05011000/05040800/06030200/08020200/10010201 |
| 95 | Prednisolone 5mg tablets | Prednisolone | 5mg | 01050200/03020000/05011000/05040800/06030200/08020200/10010201 |
| 557 | Prednisolone 2.5mg gastro-resistant tablets | Prednisolone | 2.5mg | 01050200/03020000/05011000/05040800/06030200/08020200/10010201 |
| 578 | Prednisolone 1mg tablets | Prednisolone | 1mg | 01050200/03020000/05011000/05040800/06030200/08020200/10010201 |
| 2368 | Prednisolone 2.5mg tablet | Prednisolone | 2.5mg | 6030200 |
| 2390 | PREDNISOLONE E/C 1 MG TAB | Prednisolone |  | 0 |
| 2704 | Prednisolone 25mg tablets | Prednisolone | 25mg | 01050200/03020000/06030200/08020200/10010201 |
| 2799 | PREDNISOLONE 10 MG TAB | Prednisolone |  | 0 |
| 3059 | PREDNISOLONE 50 MG TAB | Prednisolone | 50mg | 0 |
| 5490 | Deltacortril 5mg gastro-resistant tablets (Alliance Pharmaceuticals Ltd) | Prednisolone | 5mg | 01050200/03020000/05011000/05040800/06030200/08020200/10010201 |
| 5913 | Deltacortril 2.5mg gastro-resistant tablets (Alliance Pharmaceuticals Ltd) | Prednisolone | 2.5mg | 01050200/03020000/05011000/05040800/06030200/08020200/10010201 |
| 7584 | PREDNISOLONE 4 MG TAB | Prednisolone | 4mg | 0 |
| 7710 | PREDNISOLONE 15 MG TAB | Prednisolone |  | 0 |
| 9727 | Prednisolone 50mg tablets | Prednisolone | 50mg | 0 |
| 13522 | PREDNISOLONE 2 MG TAB | Prednisolone |  | 0 |
| 20095 | Precortisyl forte 25mg Tablet (Aventis Pharma) | Prednisolone | 25mg | 6030200 |
| 21417 | Prednisolone 5mg tablets (A A H Pharmaceuticals Ltd) | Prednisolone | 5mg | 01050200/03020000/05011000/05040800/06030200/08020200/10010201 |
| 23512 | Precortisyl 5mg Tablet (Hoechst Marion Roussel) | Prednisolone | 5mg | 6030200 |
| 25272 | Precortisyl 1mg Tablet (Hoechst Marion Roussel) | Prednisolone | 1mg | 6030200 |
| 27962 | Deltastab 1mg Tablet (Waymade Healthcare Plc) | Prednisolone | 1mg | 6030200 |
| 28375 | Prednisolone 2.5mg gastro-resistant tablets (A A H Pharmaceuticals Ltd) | Prednisolone | 2.5mg | 01050200/03020000/05011000/05040800/06030200/08020200/10010201 |
| 28376 | Prednisolone 2.5mg Gastro-resistant tablet (Biorex Laboratories Ltd) | Prednisolone | 2.5mg | 6030200 |
| 28859 | Deltastab 5mg Tablet (Waymade Healthcare Plc) | Prednisolone | 5mg | 6030200 |
| 29333 | Prednisolone 5mg tablets (Actavis UK Ltd) | Prednisolone | 5mg | 01050200/03020000/05011000/05040800/06030200/08020200/10010201 |
| 31532 | Prednisolone 5mg gastro-resistant tablets (A A H Pharmaceuticals Ltd) | Prednisolone | 5mg | 01050200/03020000/05011000/05040800/06030200/08020200/10010201 |
| 32803 | Prednisolone 5mg gastro-resistant tablets (Actavis UK Ltd) | Prednisolone | 5mg | 01050200/03020000/05011000/05040800/06030200/08020200/10010201 |
| 32835 | Prednisolone 5mg tablets (Wockhardt UK Ltd) | Prednisolone | 5mg | 01050200/03020000/05011000/05040800/06030200/08020200/10010201 |
| 33691 | Prednisolone 5mg Gastro-resistant tablet (Biorex Laboratories Ltd) | Prednisolone | 5mg | 6030200 |
| 33988 | Prednisolone 5mg Tablet (Co-Pharma Ltd) | Prednisolone | 5mg | 6030200 |
| 33990 | Prednisolone 5mg Tablet (IVAX Pharmaceuticals UK Ltd) | Prednisolone | 5mg | 6030200 |
| 34109 | Prednisolone 5 mg gastro-resistant tablet | Prednisolone | 5mg | 6030200 |
| 34393 | Prednisolone 5mg gastro-resistant tablets (Teva UK Ltd) | Prednisolone | 5mg | 01050200/03020000/05011000/05040800/06030200/08020200/10010201 |
| 34404 | Prednisolone 1mg tablets (Actavis UK Ltd) | Prednisolone | 1mg | 01050200/03020000/05011000/05040800/06030200/08020200/10010201 |
| 34452 | Prednisolone 1mg tablets (A A H Pharmaceuticals Ltd) | Prednisolone | 1mg | 01050200/03020000/05011000/05040800/06030200/08020200/10010201 |
| 34461 | Prednisolone 2.5mg gastro-resistant tablets (Actavis UK Ltd) | Prednisolone | 2.5mg | 01050200/03020000/05011000/05040800/06030200/08020200/10010201 |
| 34631 | Prednisolone 1mg Tablet (Co-Pharma Ltd) | Prednisolone | 1mg | 6030200 |
| 34660 | Prednisolone 1mg tablets (Kent Pharmaceuticals Ltd) | Prednisolone | 1mg | 01050200/03020000/05011000/05040800/06030200/08020200/10010201 |
| 34748 | Prednisolone 1mg tablets (Teva UK Ltd) | Prednisolone | 1mg | 01050200/03020000/05011000/05040800/06030200/08020200/10010201 |
| 34781 | Prednisolone 5mg tablets (Kent Pharmaceuticals Ltd) | Prednisolone | 5mg | 01050200/03020000/05011000/05040800/06030200/08020200/10010201 |
| 34914 | Prednisolone 1mg Tablet (Celltech Pharma Europe Ltd) | Prednisolone | 1mg | 6030200 |
| 34978 | Prednisolone 1mg tablets (Wockhardt UK Ltd) | Prednisolone | 1mg | 01050200/03020000/05011000/05040800/06030200/08020200/10010201 |
| 38407 | Prednisolone 20mg tablet | Prednisolone | 20mg | 6030200 |
| 41515 | Prednisolone 5mg tablets (Teva UK Ltd) | Prednisolone | 5mg | 01050200/03020000/05011000/05040800/06030200/08020200/10010201 |
| 41745 | Prednisolone 25mg tablets (Zentiva) | Prednisolone | 25mg | 01050200/03020000/06030200/08020200/10010201 |
| 45302 | Prednisolone 5mg Tablet (Biorex Laboratories Ltd) | Prednisolone | 5mg | 6030200 |
| 51753 | Prednisolone 1mg tablets (Strides Shasun (UK) Ltd) | Prednisolone | 1mg | 01050200/03020000/05011000/05040800/06030200/08020200/10010201 |
| 53313 | Prednisolone 20mg/5ml oral suspension | Prednisolone | 4mg/1ml | 01050200/03020000/05011000/05040800/06030200/08020200/10010201/10020100 |
| 53336 | Prednisolone 25mg tablets (A A H Pharmaceuticals Ltd) | Prednisolone | 25mg | 01050200/03020000/06030200/08020200/10010201 |
| 54118 | Prednisolone 25mg/5ml oral suspension | Prednisolone | 5mg/1ml | 01050200/03020000/05011000/05040800/06030200/08020200/10010201/10020100 |
| 54434 | Prednisolone 2.5mg/5ml oral suspension | Prednisolone | 500microgram/1ml | 01050200/03020000/05011000/05040800/06030200/08020200/10010201/10020100 |
| 55024 | Prednisolone 5mg/5ml oral solution | Prednisolone | 1mg/1ml | 03020000/06030200/10010201 |
| 55480 | Prednisolone 2.5mg gastro-resistant tablets (Alliance Pharmaceuticals Ltd) | Prednisolone | 2.5mg | 01050200/03020000/05011000/05040800/06030200/08020200/10010201 |
| 56891 | Prednisolone 1mg tablets (Waymade Healthcare Plc) | Prednisolone | 1mg | 01050200/03020000/05011000/05040800/06030200/08020200/10010201 |
| 58000 | Prednisolone 5mg tablets (Almus Pharmaceuticals Ltd) | Prednisolone | 5mg | 01050200/03020000/05011000/05040800/06030200/08020200/10010201 |
| 58234 | Prednisolone 10mg/5ml oral solution | Prednisolone | 2mg/1ml | 01050200/03020000/05011000/05040800/06030200/08020200/10010201/10020100 |
| 58369 | Prednisolone 5mg tablets (Boston Healthcare Ltd) | Prednisolone | 5mg | 01050200/03020000/05011000/05040800/06030200/08020200/10010201 |
| 58384 | Prednisolone 1mg tablets (Almus Pharmaceuticals Ltd) | Prednisolone | 1mg | 01050200/03020000/05011000/05040800/06030200/08020200/10010201 |
| 58987 | Prednisolone 5mg gastro-resistant tablets (Phoenix Healthcare Distribution Ltd) | Prednisolone | 5mg | 01050200/03020000/05011000/05040800/06030200/08020200/10010201 |
| 59229 | Dilacort 5mg gastro-resistant tablets (Auden McKenzie (Pharma Division) Ltd) | Prednisolone | 5mg | 01050200/03020000/05011000/05040800/06030200/08020200/10010201 |
| 59283 | Dilacort 2.5mg gastro-resistant tablets (Auden McKenzie (Pharma Division) Ltd) | Prednisolone | 2.5mg | 01050200/03020000/05011000/05040800/06030200/08020200/10010201 |
| 59338 | Prednisolone 1mg/5ml oral solution | Prednisolone | 200microgram/1ml | 01050200/03020000/05011000/05040800/06030200/08020200/10010201/10020100 |
| 59912 | Prednisolone 5mg gastro-resistant tablets (Waymade Healthcare Plc) | Prednisolone | 5mg | 01050200/03020000/05011000/05040800/06030200/08020200/10010201 |
| 60421 | Prednisolone 5mg tablets (Strides Shasun (UK) Ltd) | Prednisolone | 5mg | 01050200/03020000/05011000/05040800/06030200/08020200/10010201 |
| 61132 | Prednisolone 1mg tablets (Boston Healthcare Ltd) | Prednisolone | 1mg | 01050200/03020000/05011000/05040800/06030200/08020200/10010201 |
| 61162 | Prednisolone 5mg tablets (Waymade Healthcare Plc) | Prednisolone | 5mg | 01050200/03020000/05011000/05040800/06030200/08020200/10010201 |
| 63066 | Prednisolone 2.5mg tablets | Prednisolone | 2.5mg | 01050200/03020000/05011000/05040800/06030200/08020200/10010201 |
| 63082 | Prednisolone 20mg tablets | Prednisolone | 20mg | 01050200/03020000/05011000/05040800/06030200/08020200/10010201 |
| 63172 | Prednisolone 10mg tablets | Prednisolone |  | 01050200/03020000/05011000/05040800/06030200/08020200/10010201 |
| 63549 | Prednisolone 1mg/ml oral solution (Logixx Pharma Solutions Ltd) | Prednisolone | 1mg/1ml | 03020000/06030200/10010201 |
| 63791 | Prednisolone 5mg/5ml oral solution unit dose | Prednisolone |  | 03020000/06030200/10010201 |
| 64007 | Pevanti 10mg tablets (AMCo) | Prednisolone | 10mg | 01050200/03020000/05011000/05040800/06030200/08020200/10010201 |
| 64008 | Pevanti 2.5mg tablets (AMCo) | Prednisolone | 2.5mg | 01050200/03020000/05011000/05040800/06030200/08020200/10010201 |
| 64128 | Pevanti 5mg tablets (AMCo) | Prednisolone | 5mg | 01050200/03020000/05011000/05040800/06030200/08020200/10010201 |
| 64221 | Prednisolone 5mg/5ml oral suspension | Prednisolone | 1mg/1ml | 01050200/03020000/05011000/05040800/06030200/08020200/10010201/10020100 |
| 64416 | Prednisolone 10mg/ml oral solution sugar free | Prednisolone |  | 01050200/03020000/05011000/05040800/06030200/08020200/10010201/10020100 |
| 65020 | Prednisolone 25mg/5ml oral solution | Prednisolone | 25mg/5ml | 01050200/03020000/05011000/05040800/06030200/08020200/10010201/10020100 |
| 65626 | Prednisolone 10mg/5ml oral suspension | Prednisolone | 2mg/1ml | 01050200/03020000/05011000/05040800/06030200/08020200/10010201/10020100 |
| 66015 | Prednisolone Dompe 5mg/5ml oral solution unit dose (Logixx Pharma Solutions Ltd) | Prednisolone |  | 03020000/06030200/10010201 |
| 66550 | Prednisolone 5mg gastro-resistant tablets (Alliance Healthcare (Distribution) Ltd) | Prednisolone | 5mg | 01050200/03020000/05011000/05040800/06030200/08020200/10010201 |
| 66645 | Prednisolone 5mg/5ml oral solution unit dose (Logixx Pharma Solutions Ltd) | Prednisolone |  | 03020000/06030200/10010201 |
| 66914 | Prednisolone 1mg gastro-resistant tablets | Prednisolone |  | 01050200/03020000/05011000/05040800/06030200/08020200/10010201 |
| 67076 | Prednisolone 20mg/5ml oral solution | Prednisolone | 20mg/5ml | 01050200/03020000/05011000/05040800/06030200/08020200/10010201/10020100 |
| 67107 | Prednisolone 5mg gastro-resistant tablets (Alliance Pharmaceuticals Ltd) | Prednisolone | 5mg | 01050200/03020000/05011000/05040800/06030200/08020200/10010201 |
| 67507 | Prednisolone 30mg tablets | Prednisolone |  | 01050200/03020000/05011000/05040800/06030200/08020200/10010201 |
| 67559 | Prednisolone 5mg/5ml oral solution unit dose (A A H Pharmaceuticals Ltd) | Prednisolone | 1mg/1ml | 03020000/06030200/10010201 |
| 68497 | Prednisolone 2.5mg gastro-resistant tablets (Waymade Healthcare Plc) | Prednisolone | 2.5mg | 01050200/03020000/05011000/05040800/06030200/08020200/10010201 |
| 955 | Prednisolone 5mg soluble tablets | Prednisolone sodium phosphate | 5mg | 01050200/03020000/05011000/05040800/06030200/08020200/10010201 |
| 1063 | Prednesol 5mg Tablet (Sovereign Medical Ltd) | Prednisolone sodium phosphate | 5mg | 6030200 |
| 19141 | Prednisolone 5mg soluble tablets (AMCo) | Prednisolone sodium phosphate | 5mg | 01050200/03020000/05011000/05040800/06030200/08020200/10010201 |
| 47142 | Prednisolone 5mg Soluble tablet (Amdipharm Plc) | Prednisolone sodium phosphate | 5mg | 6030200 |
| 61689 | Prednisolone 5mg soluble tablets (A A H Pharmaceuticals Ltd) | Prednisolone sodium phosphate | 5mg | 01050200/03020000/05011000/05040800/06030200/08020200/10010201 |
| 63214 | Prednisolone 5mg soluble tablets (Alliance Healthcare (Distribution) Ltd) | Prednisolone sodium phosphate | 5mg | 01050200/03020000/05011000/05040800/06030200/08020200/10010201 |
| 3345 | Sintisone Tablet (Pharmacia Ltd) | Prednisolone Steaglate |  | 01050200/06030200 |
| 31327 | Prednisolone steaglate 6.65mg tablet | Prednisolone Steaglate | 6.65mg | 01050200/06030200 |
| 2044 | PREDNISONE 2.5 MG TAB | Prednisone |  | 0 |
| 2949 | Prednisone 5mg tablets | Prednisone | 5mg | 6030200 |
| 3557 | Prednisone 1mg tablets | Prednisone | 1mg | 6030200 |
| 7934 | PREDNISONE 30 MG TAB | Prednisone |  | 0 |
| 13615 | PREDNISONE 10 MG TAB | Prednisone |  | 0 |
| 16724 | PREDNISONE 50 MG TAB | Prednisone |  | 0 |
| 21833 | Decortisyl 5mg Tablet (Roussel Laboratories Ltd) | Prednisone | 5mg | 01050200/06030200 |
| 43544 | Prednisone 5mg Tablet (Knoll Ltd) | Prednisone | 5mg | 01050200/06030200 |
| 44380 | Prednisone 1mg modified-release tablets | Prednisone | 1mg | 6030200 |
| 44723 | Prednisone 5mg modified-release tablets | Prednisone | 5mg | 6030200 |
| 44802 | Lodotra 5mg modified-release tablets (Napp Pharmaceuticals Ltd) | Prednisone | 5mg | 6030200 |
| 44803 | Lodotra 2mg modified-release tablets (Napp Pharmaceuticals Ltd) | Prednisone | 2mg | 6030200 |
| 46711 | Prednisone 2mg modified-release tablets | Prednisone | 2mg | 6030200 |
| 54432 | Lodotra 1mg modified-release tablets (Napp Pharmaceuticals Ltd) | Prednisone | 1mg | 6030200 |
| 58061 | Prednisone 50mg tablets | Prednisone | 50mg | 6030200 |
| 62656 | Prednisone 5mg Tablet (Hillcross Pharmaceuticals Ltd) | Prednisone | 5mg | 01050200/06030200 |
| 64009 | Pevanti 20mg tablets (AMCo) |  |  | 01050200/03020000/05011000/05040800/06030200/08020200/10010201 |

Supplementary Table 35: LAMA product codes

| **Product code** | **Product name** | **Drug substance name** | **Substance strength** | **BNF code** |
| --- | --- | --- | --- | --- |
| 49227 | Aclidinium bromide 375micrograms/dose dry powder inhaler | Aclidinium bromide | 375microgram/1dose | 3010200 |
| 68729 | Braltus 10microgram inhalation powder capsules with Zonda inhaler (Teva UK Ltd) | Tiotropium bromide | 10 microgram | 3010200 |
| 49228 | Eklira 322micrograms/dose Genuair (AstraZeneca UK Ltd) | Aclidinium bromide | 375microgram/1dose | 3010200 |
| 63992 | Eklira 322micrograms/dose Genuair (Waymade Healthcare Plc) | Aclidinium bromide | 375microgram/1dose | 3010200 |
| 53761 | Glycopyrronium bromide 55microgram inhalation powder capsules with device | Glycopyrronium bromide |  |  |
| 67531 | Glycopyrronium bromide 55microgram inhalation powder capsules with device (J M McGill Ltd) | Glycopyrronium bromide |  | 3010200 |
| 61879 | Incruse Ellipta 55micrograms/dose dry powder inhaler (GlaxoSmithKline UK Ltd) | Umeclidinium bromide |  | 3010200 |
| 53982 | Seebri Breezhaler 44microgram inhalation powder capsules with device (Novartis Pharmaceuticals UK Ltd) | Glycopyrronium bromide | 44microgram/1dose | 3010200 |
| 6050 | Spiriva 18 microgram Capsule (Boehringer Ingelheim Ltd) | Tiotropium Bromide | 18 Microgram | 3010200 |
| 35000 | Spiriva 18microgram inhalation powder capsules (Boehringer Ingelheim Ltd) | Tiotropium bromide | 18microgram | 3010200 |
| 51967 | Spiriva 18microgram inhalation powder capsules (Mawdsley-Brooks & Company Ltd) | Tiotropium bromide | 18microgram | 3010200 |
| 50292 | Spiriva 18microgram inhalation powder capsules (Sigma Pharmaceuticals Plc) | Tiotropium Bromide |  | 3010200 |
| 34995 | Spiriva 18microgram inhalation powder capsules with HandiHaler (Boehringer Ingelheim Ltd) | Tiotropium bromide | 18microgram | 3010200 |
| 50577 | Spiriva 18microgram inhalation powder capsules with HandiHaler (DE Pharmaceuticals) | Tiotropium bromide | 18microgram | 3010200 |
| 59638 | Spiriva 18microgram inhalation powder capsules with HandiHaler (Sigma Pharmaceuticals Plc) | Tiotropium Bromide |  | 3010200 |
| 50103 | Spiriva 18microgram inhalation powder capsules with HandiHaler (Waymade Healthcare Plc) | Tiotropium Bromide |  | 3010200 |
| 36869 | Spiriva Respimat 2.5micrograms/dose solution for inhalation cartridge with device (Boehringer Ingelheim Ltd) | Tiotropium bromide | 2.5microgram/1dose | 3010200 |
| 61582 | Spiriva Respimat 2.5micrograms/dose solution for inhalation cartridge with device (Waymade Healthcare Plc) | Tiotropium bromide | 2.5microgram/1dose | 3010200 |
| 746 | Tiotropium 18 microgram Capsule | Tiotropium Bromide | 18 Microgram | 3010200 |
| 68530 | Tiotropium bromide 10microgram inhalation powder capsules with device | Tiotropium Bromide |  | 3010200 |
| 35011 | Tiotropium bromide 18microgram inhalation powder capsules | Tiotropium Bromide Monohydrate | 18 Micrograms | 3010200 |
| 35014 | Tiotropium bromide 18microgram inhalation powder capsules with device | Tiotropium Bromide Monohydrate | 18 Micrograms | 3010200 |
| 36864 | Tiotropium bromide 2.5micrograms/dose solution for inhalation cartridge with device CFC free | Tiotropium bromide | 2.5microgram/1dose | 3010200 |
| 64232 | Tiotropium bromide 2.5micrograms/dose solution for inhalation cartridge with device CFC free (AM Distributions (Yorkshire) Ltd) | Tiotropium bromide | 2.5microgram/1dose | 3010200 |
| 62109 | Umeclidinium bromide 65micrograms/dose dry powder inhaler | Umeclidinium bromide |  | 3010200 |
| 62109 | Umeclidinium bromide 65micrograms/dose dry powder inhaler | Umeclidinium bromide |  | 3010200 |

Supplementary Table 36: Combination anticholinergics/BA product codes

| **Product code** | **Product name** | **Drug substance name** | **Substance strength** | **BNF code** |
| --- | --- | --- | --- | --- |
| 2722 | Duovent inhaler (Boehringer Ingelheim Ltd) | Fenoterol hydrobromide/Ipratropium bromide | 100microgram/1dose + 40microgram/1dose | 0 |
| 3786 | Fenoterol 100micrograms/dose / Ipratropium 40micrograms/dose inhaler | Fenoterol hydrobromide/Ipratropium bromide | 100microgram/1dose + 40microgram/1dose | 0 |
| 16207 | Duovent UDVs nebuliser liquid 4ml (Boehringer Ingelheim Ltd) | Fenoterol hydrobromide/Ipratropium bromide | 312.5microgram/1ml + 125microgram/1ml | 3010400 |
| 2862 | Duovent Autohaler (Boehringer Ingelheim Ltd) | Ipratropium bromide/Fenoterol hydrobromide | 40microgram/1dose + 100microgram/1dose | 0 |
| 9270 | Ipratropium bromide with fenoterol hydrobromide 500micrograms + 1.25mg/4ml | Ipratropium Bromide/Fenoterol Hydrobromide | 500micrograms + 1.25mg/4ml | 03010101/03010400 |
| 12808 | Fenoterol 100micrograms/dose / Ipratropium bromide 40micrograms/dose breath actuated inhaler | Ipratropium bromide/Fenoterol hydrobromide | 40microgram/1dose + 100microgram/1dose | 0 |
| 26616 | Ipratropium bromide with fenoterol hydrobromide 0micrograms + 100micrograms/actuation | Ipratropium Bromide/Fenoterol Hydrobromide | 0micrograms + 100micrograms/actuation | 03010101/03010400 |
| 27505 | Ipratropium bromide with fenoterol hydrobromide 40micrograms + 100micrograms/actuation | Ipratropium Bromide/Fenoterol Hydrobromide | 40micrograms + 100micrograms/actuation | 03010101/03010400 |
| 43046 | Salipraneb 0.5mg/2.5mg nebuliser solution 2.5ml ampoules (Arrow Generics Ltd) | Ipratropium bromide/Salbutamol sulfate | 200microgram/1ml + 1mg/1ml | 3010400 |
| 48410 | Salbutamol 2.5mg/2.5ml / Ipratropium bromide 500micrograms/2.5ml nebuliser liquid ampoules | Ipratropium bromide/Salbutamol sulfate | 200microgram/1ml + 1mg/1ml | 3010400 |
| 68844 | Salipraneb 0.5mg/2.5mg nebuliser solution 2.5ml ampoules (Actavis UK Ltd) | Ipratropium bromide/Salbutamol sulfate | 200microgram/1ml + 1mg/1ml | 3010400 |
| 556 | Combivent inhaler (Boehringer Ingelheim Ltd) | Salbutamol sulfate/Ipratropium bromide | 100microgram/1dose + 20microgram/1dose | 0 |
| 3305 | Combivent nebuliser liquid 2.5ml UDVs (Boehringer Ingelheim Ltd) | Salbutamol sulfate/Ipratropium bromide | 1mg/1ml + 200microgram/1ml | 3010400 |
| 12909 | Salbutamol 100micrograms/dose / Ipratropium 20micrograms/dose inhaler | Salbutamol sulfate/Ipratropium bromide | 100microgram/1dose + 20microgram/1dose | 0 |
| 35557 | Ipramol nebuliser solution 2.5ml Steri-Neb unit dose vials (Teva UK Ltd) | Salbutamol sulfate/Ipratropium bromide | 1mg/1ml + 200microgram/1ml | 3010400 |
| 48607 | Salbutamol 2.5mg/2.5ml / Ipratropium bromide 500micrograms/2.5ml nebuliser liquid unit dose vials | Salbutamol sulfate/Ipratropium bromide | 1mg/1ml + 200microgram/1ml | 3010400 |
| 49904 | Combivent nebuliser liquid 2.5ml UDVs (Lexon (UK) Ltd) | Salbutamol sulfate/Ipratropium bromide | 1mg/1ml + 200microgram/1ml | 3010400 |
| 51903 | Combivent nebuliser liquid 2.5ml UDVs (DE Pharmaceuticals) | Salbutamol sulfate/Ipratropium bromide | 1mg/1ml + 200microgram/1ml | 3010400 |
| 2152 | Ipratropium bromide with salbutamol 20mcg + 100mcg | Salbutamol Sulphate/Ipratropium Bromide | 20mcg + 100mcg | 03010101/03010400 |
| 11046 | Ipratropium bromide with salbutamol 500micrograms + 2.5mg/2.5ml | Salbutamol Sulphate/Ipratropium Bromide | 500micrograms + 2.5mg/2.5ml | 03010101/03010200 |
| 12822 | Salbutamol 2.5mg with ipratropium bromide 500micrograms/2.5ml unit dose nebuilser solution | Salbutamol Sulphate/Ipratropium Bromide | 2.5mg + 500micrograms/2.5ml | 03010101/03010200 |
| 64509 | Tiotropium bromide 2.5micrograms/dose / Olodaterol 2.5micrograms/dose solution for inhalation cartridge with device CFC free | Tiotropium bromide/Olodaterol hydrochloride | 2.5microgram/1dose + 2.5microgram/1dose | 3010400 |
| 64523 | Spiolto Respimat 2.5micrograms/dose / 2.5micrograms/dose solution for inhalation cartridge with device (Boehringer Ingelheim Ltd) | Tiotropium bromide/Olodaterol hydrochloride | 2.5microgram/1dose + 2.5microgram/1dose | 3010400 |
| 12909 | Salbutamol 100micrograms/dose / Ipratropium 20micrograms/dose inhaler | Salbutamol sulfate/Ipratropium bromide | 100microgram/1dose + 20microgram/1dose | 0 |
| 48607 | Salbutamol 2.5mg/2.5ml / Ipratropium bromide 500micrograms/2.5ml nebuliser liquid unit dose vials | Salbutamol sulfate/Ipratropium bromide | 1mg/1ml + 200microgram/1ml | 3010400 |
| 11046 | Ipratropium bromide with salbutamol 500micrograms + 2.5mg/2.5ml | Salbutamol Sulphate/Ipratropium Bromide | 500micrograms + 2.5mg/2.5ml | 03010101/03010200 |
| 18299 | Fenoterol 1.25mg/4ml / Ipratropium 500micrograms/4ml nebuliser liquid unit dose vials | Fenoterol hydrobromide/Ipratropium bromide | 312.5microgram/1ml + 125microgram/1ml | 3010400 |
| *62535* | *Duaklir 340micrograms/dose / 12micrograms/dose Genuair (AstraZeneca UK Ltd)* | *Formoterol fumarate dihydrate/Aclidinium bromide* | *11.8microgram/1dose + 396microgram/1dose* | *3010400* |
| *62838* | *Aclidinium bromide 396micrograms/dose / Formoterol 11.8micrograms/dose dry powder inhaler* | *Formoterol fumarate dihydrate/Aclidinium bromide* | *11.8microgram/1dose + 396microgram/1dose* | *3010400* |
| *61176* | *Anoro Ellipta 55micrograms/dose / 22micrograms/dose dry powder inhaler (GlaxoSmithKline UK Ltd)* | *Vilanterol trifenatate/Umeclidinium bromide* | *22microgram/1dose + 65microgram/1dose* | *3010400* |

Supplementary Table 37: SABA product codes

| **Product code** | **Product name** | **Drug substance name** | **Substance strength** | **BNF code** |
| --- | --- | --- | --- | --- |
| 8 | Salbutamol 100micrograms/dose inhaler | Salbutamol | 100microgram/1dose | 0 |
| 17 | Salbutamol 100micrograms/dose inhaler CFC free | Salbutamol sulphate | 100microgram/1dose | 3010101 |
| 31 | Ventolin 100microgram/inhalation Inhalation powder (Glaxo Wellcome UK Ltd) | Salbutamol | 100microgram/1dose | 3010000 |
| 235 | Bricanyl 250micrograms/dose inhaler (AstraZeneca UK Ltd) | Terbutaline sulphate | 250microgram/1dose | 0 |
| 461 | Orciprenaline 750micrograms/inhalation inhaler | Orciprenaline Sulphate | 750micrograms/inhalation | 3010102 |
| 510 | Ventolin 5mg/ml respirator solution (GlaxoSmithKline UK Ltd) | Salbutamol sulphate | 5mg/1ml | 3010101 |
| 674 | Ventolin 2.5mg Nebules (GlaxoSmithKline UK Ltd) | Salbutamol sulphate | 1mg/1ml | 3010101 |
| 862 | Salbulin Inhalation powder (3M Health Care Ltd) | Salbutamol | 100microgram/1dose | 3010000 |
| 882 | Salbutamol 200microgram inhalation powder capsules | Salbutamol sulphate | 200microgram | 3010101 |
| 898 | Ventolin evohaler 100 100microgram/inhalation Pressurised inhalation (Glaxo Wellcome UK Ltd) | Salbutamol sulphate | 100microgram/1dose | 3010000 |
| 907 | Bricanyl turbohaler 500 500microgram Turbohaler (AstraZeneca UK Ltd) | Terbutaline sulphate | 500microgram/1dose | 3010000 |
| 942 | Aerolin 100micrograms/dose Autohaler (3M Health Care Ltd) | Salbutamol sulphate | 100microgram/1dose | 0 |
| 957 | Salamol easi-breathe 100microgram/actuation Pressurised inhalation (IVAX Pharmaceuticals UK Ltd) | Salbutamol sulphate | 100microgram/1dose | 3010000 |
| 958 | Ventolin easi-breathe 100microgram/actuation Pressurised inhalation (Allen & Hanburys Ltd) | Salbutamol sulphate | 100microgram/1dose | 3010000 |
| 1087 | Asmasal 95micrograms/dose Clickhaler (Focus Pharmaceuticals Ltd) | Salbutamol sulphate | 95microgram/1dose | 3010101 |
| 1093 | Salamol 100microgram/actuation Inhalation powder (IVAX Pharmaceuticals UK Ltd) | Salbutamol | 100microgram/1dose | 3010000 |
| 1414 | Salamol 5mg/2.5ml nebuliser liquid Steri-Neb unit dose vials (Teva UK Ltd) | Salbutamol sulphate | 2mg/1ml | 3010101 |
| 1619 | Terbutaline 500micrograms/dose dry powder inhaler | Terbutaline sulphate | 500microgram/1dose | 3010101 |
| 1620 | Terbutaline 250micrograms/dose inhaler | Terbutaline sulphate | 250microgram/1dose | 0 |
| 1628 | Terbutaline 250micrograms/actuation refill canister | Terbutaline Sulphate | 250micrograms/actuation | 3010000 |
| 1630 | Salbutamol 2.5mg/2.5ml nebuliser liquid unit dose vials | Salbutamol sulphate | 1mg/1ml | 3010101 |
| 1698 | Salbutamol 100micrograms/dose breath actuated inhaler | Salbutamol sulphate | 100microgram/1dose | 0 |
| 1711 | Salbutamol 5mg/2.5ml nebuliser liquid unit dose vials | Salbutamol sulphate | 2mg/1ml | 3010101 |
| 1741 | Salbutamol 100micrograms/dose breath actuated inhaler CFC free | Salbutamol sulphate | 100microgram/1dose | 3010101 |
| 1794 | Berotec 100microgram/actuation Inhalation powder (Boehringer Ingelheim Ltd) | Fenoterol Hydrobromide | 100microgram/actuation | 3010101 |
| 1882 | Ventodisks 200microgram/blister Disc (Allen & Hanburys Ltd) | Salbutamol Sulphate | 200microgram/blister | 3010000 |
| 1950 | Ventodisks 400microgram/blister Disc (Allen & Hanburys Ltd) | Salbutamol Sulphate | 400microgram/blister | 3010000 |
| 1952 | Ventolin 400microgram Rotacaps (GlaxoSmithKline UK Ltd) | Salbutamol sulphate | 400microgram | 3010101 |
| 1957 | Ventolin 5mg Nebules (GlaxoSmithKline UK Ltd) | Salbutamol sulphate | 2mg/1ml | 3010101 |
| 2152 | Ipratropium bromide with salbutamol 20mcg + 100mcg | Salbutamol Sulphate | 20mcg + 100mcg | 03010400/03010101 |
| 2655 | Airomir 100micrograms/dose inhaler (Teva UK Ltd) | Salbutamol sulphate | 100microgram/1dose | 3010101 |
| 2758 | Bricanyl Refill canister (AstraZeneca UK Ltd) | Terbutaline Sulphate |  | 3010000 |
| 2850 | Salbutamol 400microgram inhalation powder capsules | Salbutamol sulphate | 400microgram | 3010101 |
| 2851 | Ventolin 200microgram Rotacaps (GlaxoSmithKline UK Ltd) | Salbutamol sulphate | 200microgram | 3010101 |
| 2978 | Salbutamol 200micrograms/dose dry powder inhaler | Salbutamol | 200microgram/1dose | 3010101 |
| 3163 | Salbutamol 200micrograms disc | Salbutamol Sulphate | 200micrograms | 3010000 |
| 3443 | Salbutamol 100microgram/inhalation Spacehaler (Celltech Pharma Europe Ltd) | Salbutamol | 100microgram/inhalation | 3010000 |
| 3758 | Pulmadil Inhalation powder (3M Health Care Ltd) | Rimiterol Hydrobromide |  | 3010101 |
| 3763 | TERBUTALINE RESPULES INH |  |  | 0 |
| 3838 | SALBUTAMOL 400MCG/BECLOMETH.100MCG R/CAP INH |  |  | 0 |
| 4497 | Ventolin accuhaler 200 200microgram/actuation Inhalation powder (Glaxo Wellcome UK Ltd) | Salbutamol | 200microgram/1dose | 3010000 |
| 4634 | Salamol 2.5mg/2.5ml nebuliser liquid Steri-Neb unit dose vials (Teva UK Ltd) | Salbutamol sulphate | 1mg/1ml | 3010101 |
| 4665 | Salbulin 100micrograms/dose inhaler (3M Health Care Ltd) | Salbutamol sulphate | 100microgram/1dose | 3010101 |
| 4842 | Fenoterol 100microgram/actuation inhaler | Fenoterol Hydrobromide | 100microgram/actuation | 3010101 |
| 5170 | Salamol 100micrograms/dose inhaler CFC free (Teva UK Ltd) | Salbutamol sulphate | 100microgram/1dose | 3010101 |
| 5185 | Fenoterol 200micrograms/dose inhaler | Fenoterol hydrobromide | 200microgram/1dose | 0 |
| 5308 | Terbutaline 5mg/2ml nebuliser liquid unit dose vials | Terbutaline sulfate | 2.5mg/1ml | 3010101 |
| 5516 | Salamol 100micrograms/dose Easi-Breathe inhaler (Teva UK Ltd) | Salbutamol sulphate | 100microgram/1dose | 3010101 |
| 5740 | Airomir 100micrograms/dose Autohaler (Teva UK Ltd) | Salbutamol sulphate | 100microgram/1dose | 3010101 |
| 5753 | Salbutamol 400micrograms disc | Salbutamol Sulphate | 400micrograms | 3010000 |
| 5837 | Salamol steri-neb 5mg/2.5ml Nebuliser liquid (Numark Management Ltd) | Salbutamol sulphate | 2mg/1ml | 3010101 |
| 5889 | Salamol 100microgram/inhalation Inhalation powder (Kent Pharmaceuticals Ltd) | Salbutamol sulphate | 100microgram/1dose | 3010000 |
| 5898 | Salamol steri-neb 2.5mg/2.5ml Nebuliser liquid (Numark Management Ltd) | Salbutamol sulphate | 1mg/1ml | 3010101 |
| 6462 | Salbutamol 95micrograms/dose dry powder inhaler | Salbutamol sulphate | 95microgram/1dose | 3010101 |
| 7017 | Salbutamol 100micrograms/dose dry powder inhaler | Salbutamol | 100microgram/1dose | 3010101 |
| 7711 | Terbutaline 250micrograms/dose inhaler with spacer | Terbutaline sulfate | 250microgram/1dose | 0 |
| 7935 | Maxivent 100microgram/inhalation Inhalation powder (Ashbourne Pharmaceuticals Ltd) | Salbutamol | 100microgram/1dose | 3010000 |
| 7965 | Salbutamol 5mg/ml nebuliser liquid | Salbutamol sulphate | 5mg/1ml | 3010101 |
| 8151 | Orciprenaline 750micrograms/inhalation Aerosol refill | Orciprenaline Sulphate | 750micrograms/inhalation | 3010102 |
| 8339 | FENOTEROL HYDROBROMIDE COMPLETE UNIT INH |  |  | 0 |
| 8572 | Rimiterol inhaler | Rimiterol Hydrobromide |  | 3010101 |
| 8676 | Terbutaline 10mg/ml nebuliser liquid | Terbutaline sulfate | 10mg/1ml | 0 |
| 9651 | Asmasal 100microgram/inhalation Spacehaler (Celltech Pharma Europe Ltd) | Salbutamol | 100microgram/inhalation | 3010000 |
| 10858 | Pulmadil auto Inhalation powder (3M Health Care Ltd) | Rimiterol Hydrobromide |  | 3010101 |
| 11046 | Ipratropium bromide with salbutamol 500micrograms + 2.5mg/2.5ml | Salbutamol Sulphate | 500micrograms + 2.5mg/2.5ml | 03010200/03010101 |
| 12463 | PIRBUTEROL 15 MG TAB |  |  | 0 |
| 12486 | Bronchodil 500microgram/dose Inhalation powder (Viatris Pharmaceuticals Ltd) | Reproterol Hydrochloride | 500microgram/dose | 3010101 |
| 12563 | Exirel Inhalation powder (3M Health Care Ltd) | Pirbuterol |  | 3010101 |
| 12822 | Salbutamol 2.5mg with ipratropium bromide 500micrograms/2.5ml unit dose nebuilser solution | Salbutamol Sulphate | 2.5mg + 500micrograms/2.5ml | 03010101/03010200 |
| 13038 | Pulvinal Salbutamol 200micrograms/dose dry powder inhaler (Chiesi Ltd) | Salbutamol | 200microgram/1dose | 3010101 |
| 13181 | Easyhaler Salbutamol sulphate 100micrograms/dose dry powder inhaler (Orion Pharma (UK) Ltd) | Salbutamol | 100microgram/1dose | 3010101 |
| 13365 | Berotec 5mg/ml Nebuliser liquid (Boehringer Ingelheim Ltd) | Fenoterol Hydrobromide | 5mg/ml | 3010101 |
| 13996 | Salamol 100microgram/inhalation Inhalation powder (Sandoz Ltd) | Salbutamol sulphate | 100microgram/1dose | 3010000 |
| 14525 | Salbutamol 100micrograms/inhalation vortex inhaler | Salbutamol | 100micrograms/inhalation | 3010000 |
| 15165 | Reproterol 500micrograms/dose inhaler | Reproterol Hydrochloride | 500micrograms/dose | 3010101 |
| 15413 | ORCIPRENALINE SULPHATE 5% SOL |  |  | 0 |
| 15413 | ORCIPRENALINE SULPHATE 5% SOL |  |  | 0 |
| 15441 | FENOTEROL HYDROBROMIDE .5% SOL |  |  | 0 |
| 16236 | Pirbuterol acetate inhaler | Pirbuterol |  | 3010101 |
| 16577 | Easyhaler Salbutamol sulphate 200micrograms/dose dry powder inhaler (Orion Pharma (UK) Ltd) | Salbutamol | 200microgram/1dose | 3010101 |
| 21859 | Asmaven 100microgram Inhalation powder (Berk Pharmaceuticals Ltd) | Salbutamol | 100microgram/1dose | 3010000 |
| 22430 | Spacehaler salbutamol 100microgram/inhalation Spacehaler (Celltech Pharma Europe Ltd) | Salbutamol | 100microgram/inhalation | 3010000 |
| 22790 | Reproterol 10mg/ml respirator solution | Reproterol Hydrochloride | 10mg/ml | 3010101 |
| 23269 | Maxivent 2.5mg/2.5ml nebuliser liquid unit dose Steripoule vials (Ashbourne Pharmaceuticals Ltd) | Salbutamol sulphate | 1mg/1ml | 3010101 |
| 25339 | Maxivent 5mg/2.5ml nebuliser liquid unit dose Steripoule vials (Ashbourne Pharmaceuticals Ltd) | Salbutamol sulphate | 2mg/1ml | 3010101 |
| 27793 | Salbutamol cyclohaler type 5 insufflator Inhalation powder (Bristol-Myers Squibb Pharmaceuticals Ltd) |  |  | 3010502 |
| 28508 | Salbutamol 100microgram/inhalation Inhalation powder (IVAX Pharmaceuticals UK Ltd) | Salbutamol | 100microgram/1dose | 3010000 |
| 30118 | Salbutamol 100micrograms/dose inhaler CFC free (Teva UK Ltd) | Salbutamol sulphate | 100microgram/1dose | 3010101 |
| 30204 | Salbutamol 200micrograms inahalation capsules | Salbutamol Sulphate | 200micrograms | 3010000 |
| 30212 | Salbutamol cyclohaler | Salbutamol Sulphate |  | 3010000 |
| 30230 | Salbutamol 100micrograms/actuation breath actuated inhaler | Salbutamol Sulphate | 100micrograms/actuation | 3010000 |
| 30240 | Aerolin autohaler 100microgram/actuation Pressurised inhalation (3M Health Care Ltd) | Salbutamol sulphate | 100microgram/1dose | 3010000 |
| 31082 | Salbuvent 5mg/ml Respirator solution (Pharmacia Ltd) | Salbutamol sulphate | 5mg/1ml | 3010101 |
| 31933 | Salbutamol 100micrograms/dose inhaler (A A H Pharmaceuticals Ltd) | Salbutamol | 100microgram/1dose | 0 |
| 32050 | Salbutamol 400 Cyclocaps (Teva UK Ltd) | Salbutamol sulphate | 400microgram | 3010101 |
| 33089 | Salbutamol 100micrograms/dose inhaler (Kent Pharmaceuticals Ltd) | Salbutamol | 100microgram/1dose | 0 |
| 33373 | Salbutamol 200 Cyclocaps (Teva UK Ltd) | Salbutamol sulphate | 200microgram | 3010101 |
| 33588 | Salbutamol 100micrograms/dose inhaler (Generics (UK) Ltd) | Salbutamol | 100microgram/1dose | 0 |
| 33817 | Salbutamol 100micrograms/dose inhaler CFC free (Actavis UK Ltd) | Salbutamol sulphate | 100microgram/1dose | 3010101 |
| 34018 | Salbutamol 5mg/2.5ml Nebuliser liquid (Galen Ltd) | Salbutamol sulphate | 2mg/1ml | 3010101 |
| 34029 | Salbutamol 400micrograms inahalation capsules | Salbutamol Sulphate | 400micrograms | 3010000 |
| 34134 | Aerolin 400 100microgram/actuation Inhalation powder (3M Health Care Ltd) | Salbutamol | 100microgram/1dose | 3010000 |
| 34162 | Salbutamol 2.5mg/2.5ml Nebuliser liquid (Galen Ltd) | Salbutamol sulphate | 1mg/1ml | 3010101 |
| 34310 | Salbutamol 100micrograms/dose inhaler CFC free (A A H Pharmaceuticals Ltd) | Salbutamol sulphate | 100microgram/1dose | 3010101 |
| 34311 | Salbutamol 100microgram/inhalation Inhalation powder (Berk Pharmaceuticals Ltd) | Salbutamol | 100microgram/1dose | 3010000 |
| 34619 | Salbutamol 100microgram/inhalation Inhalation powder (Kent Pharmaceuticals Ltd) | Salbutamol sulphate | 100microgram/1dose | 3010000 |
| 34702 | Salbutamol 100microgram/inhalation Inhalation powder (C P Pharmaceuticals Ltd) | Salbutamol | 100microgram/1dose | 3010000 |
| 37612 | Terbutaline 5mg/2ml nebuliser liquid unit dose vials (Galen Ltd) | Terbutaline sulphate | 2.5mg/1ml | 0 |
| 38079 | Salbutamol 100micrograms/dose dry powder inhalation cartridge with device | Salbutamol Sulphate | 100micrograms | 3010101 |
| 38097 | Salbutamol cyclocaps 200microgram Inhalation powder (DuPont Pharmaceuticals Ltd) | Salbutamol sulphate | 200microgram | 3010000 |
| 38136 | Salbulin Novolizer 100micrograms/dose inhalation powder (Meda Pharmaceuticals Ltd) | Salbutamol sulphate | 100microgram/1dose | 3010101 |
| 38214 | Salbutamol 100micrograms/dose dry powder inhalation cartridge | Salbutamol Sulphate | 100micrograms | 3010101 |
| 38226 | Salbulin Novolizer 100micrograms/dose inhalation powder refill (Meda Pharmaceuticals Ltd) | Salbutamol Sulphate | 100micrograms | 3010101 |
| 38416 | Salbutamol cyclocaps 400microgram Inhalation powder (DuPont Pharmaceuticals Ltd) | Salbutamol sulphate | 400microgram | 3010000 |
| 40599 | Salbutamol 5mg/2.5ml nebuliser liquid unit dose Steripoule vials (Galen Ltd) | Salbutamol sulphate | 2mg/1ml | 3010101 |
| 40655 | Salbuvent 100microgram/actuation Inhalation powder (Pharmacia Ltd) | Salbutamol | 100microgram/1dose | 3010000 |
| 40709 | Salbutamol 2.5mg/2.5ml nebuliser liquid unit dose vials (A A H Pharmaceuticals Ltd) | Salbutamol sulphate | 1mg/1ml | 3010101 |
| 42279 | Salbutamol 2.5mg/2.5ml nebuliser liquid unit dose Steripoule vials (Galen Ltd) | Salbutamol sulphate | 1mg/1ml | 3010101 |
| 42830 | Ventolin 100micrograms/dose Evohaler (GlaxoSmithKline UK Ltd) | Salbutamol sulphate | 100microgram/1dose | 3010101 |
| 42858 | Ventolin 200micrograms/dose Accuhaler (GlaxoSmithKline UK Ltd) | Salbutamol | 200microgram/1dose | 3010101 |
| 42886 | Bricanyl 500micrograms/dose Turbohaler (AstraZeneca UK Ltd) | Terbutaline sulphate | 500microgram/1dose | 3010101 |
| 43085 | Bricanyl 5mg/2ml Respules (AstraZeneca UK Ltd) | Terbutaline sulphate | 2.5mg/1ml | 3010101 |
| 44713 | Salbutamol 100microgram/inhalation Inhalation powder (Celltech Pharma Europe Ltd) | Salbutamol | 100microgram/1dose | 3010000 |
| 45863 | Salbutamol 5mg/2.5ml Nebuliser liquid (Generics (UK) Ltd) | Salbutamol sulphate | 2mg/1ml | 3010101 |
| 46551 | Salbutamol 100microgram/inhalation Inhalation powder (Neo Laboratories Ltd) | Salbutamol sulphate | 100microgram/1dose | 3010000 |
| 48490 | Ventolin 100micrograms/dose Evohaler (Doncaster Pharmaceuticals Ltd) | Salbutamol sulphate | 100microgram/1dose | 3010101 |
| 48519 | Ventolin 100micrograms/dose Evohaler (Waymade Healthcare Plc) | Salbutamol sulphate | 100microgram/1dose | 3010101 |
| 48547 | Salamol 100micrograms/dose inhaler CFC free (Arrow Generics Ltd) | Salbutamol sulphate | 100microgram/1dose | 3010101 |
| 48741 | Ventolin 100micrograms/dose Evohaler (Mawdsley-Brooks & Company Ltd) | Salbutamol sulphate | 100microgram/1dose | 3010101 |
| 48742 | Ventodisks 400microgram (GlaxoSmithKline UK Ltd) | Salbutamol sulphate | 400microgram | 0 |
| 48809 | Ventodisks 400microgram with Diskhaler (GlaxoSmithKline UK Ltd) | Salbutamol sulphate | 400microgram | 0 |
| 49368 | Ventodisks 200microgram with Diskhaler (GlaxoSmithKline UK Ltd) | Salbutamol sulphate | 200microgram | 0 |
| 49369 | Salbutamol 200microgram inhalation powder blisters | Salbutamol sulphate | 200microgram | 0 |
| 49370 | Ventodisks 200microgram (GlaxoSmithKline UK Ltd) | Salbutamol sulphate | 200microgram | 0 |
| 49591 | Salbutamol 100micrograms/dose inhaler CFC free (Sandoz Ltd) | Salbutamol sulphate | 100microgram/1dose | 3010101 |
| 50315 | Salbutamol 200microgram inhalation powder blisters with device | Salbutamol sulphate | 200microgram | 0 |
| 50503 | Ventolin 200micrograms/dose Accuhaler (Mawdsley-Brooks & Company Ltd) | Salbutamol | 200microgram/1dose | 3010101 |
| 50557 | Ventolin 200micrograms/dose Accuhaler (Lexon (UK) Ltd) | Salbutamol | 200microgram/1dose | 3010101 |
| 50956 | Ventolin 200micrograms/dose Accuhaler (Doncaster Pharmaceuticals Ltd) | Salbutamol | 200microgram/1dose | 3010101 |
| 52410 | Bricanyl 500micrograms/dose Turbohaler (Necessity Supplies Ltd) | Terbutaline sulphate | 500microgram/1dose | 3010101 |
| 52543 | Salbutamol 400microgram inhalation powder blisters | Salbutamol sulphate | 400microgram | 0 |
| 52799 | Salbutamol 400microgram inhalation powder blisters with device | Salbutamol sulphate | 400microgram | 0 |
| 53019 | Ventolin 2.5mg Nebules (Mawdsley-Brooks & Company Ltd) | Salbutamol sulphate | 1mg/1ml | 3010101 |
| 53297 | Ventolin 200micrograms/dose Accuhaler (Sigma Pharmaceuticals Plc) | Salbutamol | 200microgram/1dose | 3010101 |
| 57249 | Asmavent 100micrograms/dose inhaler CFC free (Kent Pharmaceuticals Ltd) | Salbutamol sulfate | 100microgram/1dose | 3010101 |
| 57524 | Ventolin 200micrograms/dose Accuhaler (Dowelhurst Ltd) | Salbutamol | 200microgram/1dose | 3010101 |
| 58269 | AirSalb 100micrograms/dose inhaler CFC free (Sandoz Ltd) | Salbutamol sulfate | 100microgram/1dose | 3010101 |
| 59409 | Salbutamol 100micrograms/dose inhaler CFC free (Waymade Healthcare Plc) | Salbutamol sulfate | 100microgram/1dose | 3010101 |
| 60601 | Salbutamol 5mg/2.5ml nebuliser liquid unit dose vials (Alliance Healthcare (Distribution) Ltd) | Salbutamol sulfate | 2mg/1ml | 3010101 |
| 60923 | Salamol 100micrograms/dose Easi-Breathe inhaler (DE Pharmaceuticals) | Salbutamol sulfate | 100microgram/1dose | 3010101 |
| 61330 | Salbutamol 2.5mg/2.5ml nebuliser liquid unit dose vials (Alliance Healthcare (Distribution) Ltd) | Salbutamol sulfate | 1mg/1ml | 3010101 |
| 61591 | Salbutamol 100micrograms/dose inhaler CFC free (Phoenix Healthcare Distribution Ltd) | Salbutamol sulfate | 100microgram/1dose | 3010101 |
| 64801 | Salbutamol 100micrograms/dose inhaler CFC free (Mylan Ltd) | Salbutamol sulfate | 100microgram/1dose | 3010101 |
| 66395 | Salbutamol 100micrograms/dose inhaler CFC free (Mawdsley-Brooks & Company Ltd) | Salbutamol sulfate | 100microgram/1dose | 3010101 |
| 66793 | Salbutamol rondo 100micrograms/actuation inhaler and spacer | Salbutamol |  | 3010000 |
| 66924 | Salbutamol 100micrograms/dose inhaler CFC free (DE Pharmaceuticals) | Salbutamol sulfate | 100microgram/1dose | 3010101 |
| 66972 | Salbutamol 100micrograms/dose inhaler CFC free (AM Distributions (Yorkshire) Ltd) | Salbutamol sulfate | 100microgram/1dose | 3010101 |
| 67040 | Salbutamol 100micrograms/dose inhaler CFC free (Alliance Healthcare (Distribution) Ltd) | Salbutamol sulfate | 100microgram/1dose | 3010101 |
| 67326 | Bricanyl 500micrograms/dose Turbohaler (DE Pharmaceuticals) | Terbutaline sulfate | 500microgram/1dose | 3010101 |
| 67543 | Bricanyl 500micrograms/dose Turbohaler (Waymade Healthcare Plc) | Terbutaline sulfate | 500microgram/1dose | 3010101 |
| 68285 | Salbutamol 5mg/2.5ml nebuliser liquid unit dose vials (Actavis UK Ltd) | Salbutamol sulfate | 2mg/1ml | 3010101 |

Supplementary Table 38: SAMA product codes

| **Product code** | **Product name** | **Drug substance name** | **Substance strength** | **BNF code** |
| --- | --- | --- | --- | --- |
| 1697 | Atrovent 20micrograms/dose Autohaler (Boehringer Ingelheim Ltd) | Ipratropium bromide | 20microgram/1dose | 3010200 |
| 534 | Atrovent 20micrograms/dose inhaler (Boehringer Ingelheim Ltd) | Ipratropium bromide | 20microgram/1dose | 3010200 |
| 6512 | Atrovent 20micrograms/dose inhaler CFC free (Boehringer Ingelheim Ltd) | Ipratropium bromide | 20microgram/1dose | 3010200 |
| 50810 | Atrovent 20micrograms/dose inhaler CFC free (DE Pharmaceuticals) | Ipratropium bromide | 20microgram/1dose | 3010200 |
| 57557 | Atrovent 20micrograms/dose inhaler CFC free (Lexon (UK) Ltd) | Ipratropium bromide | 20microgram/1dose | 3010200 |
| 60920 | Atrovent 20micrograms/dose inhaler CFC free (Sigma Pharmaceuticals Plc) | Ipratropium bromide | 20microgram/1dose | 3010200 |
| 6911 | Atrovent 250micrograms/1ml nebuliser liquid UDVs (Boehringer Ingelheim Ltd) | Ipratropium bromide | 250microgram/1ml | 3010200 |
| 43090 | Atrovent 40microgram Aerocaps (Boehringer Ingelheim Ltd) | Ipratropium bromide | 40microgram | 3010200 |
| 43105 | Atrovent 40microgram Aerocaps with Aerohaler (Boehringer Ingelheim Ltd) | Ipratropium bromide | 40microgram | 3010200 |
| 7140 | Atrovent 500micrograms/2ml nebuliser liquid UDVs (Boehringer Ingelheim Ltd) | Ipratropium bromide | 250microgram/1ml | 3010200 |
| 55132 | Atrovent 500micrograms/2ml nebuliser liquid UDVs (Waymade Healthcare Plc) | Ipratropium bromide | 250microgram/1ml | 3010200 |
| 2994 | Atrovent aerocaps 40microgram Inhalation powder (Boehringer Ingelheim Ltd) | Ipratropium bromide | 40microgram | 3010200 |
| 9681 | Atrovent aerohaler 40microgram Inhalation powder (Boehringer Ingelheim Ltd) | Ipratropium bromide | 40microgram | 3010200 |
| 3306 | Atrovent Forte 40micrograms/dose inhaler (Boehringer Ingelheim Ltd) | Ipratropium bromide | 40microgram/1dose | 3010200 |
| 1962 | Atrovent udv 0.25mg/ml Nebuliser liquid (Boehringer Ingelheim Ltd) | Ipratropium Bromide | 0.25mg/ml | 3010200 |
| 6758 | Ipratropium 250micrograms/1ml nebuliser liquid Steri-Neb unit dose vials (Teva UK Ltd) | Ipratropium bromide | 250microgram/1ml | 3010200 |
| 40637 | Ipratropium 250micrograms/1ml nebuliser liquid unit dose Steripoule vials (Galen Ltd) | Ipratropium bromide | 250microgram/1ml | 3010200 |
| 23709 | Ipratropium 500micrograms/2ml nebuliser liquid Steri-Neb unit dose vials (Teva UK Ltd) | Ipratropium bromide | 250microgram/1ml | 3010200 |
| 40832 | Ipratropium 500micrograms/2ml nebuliser liquid unit dose Steripoule vials (Galen Ltd) | Ipratropium bromide | 250microgram/1ml | 3010200 |
| 1410 | Ipratropium bromide 0.25mg/ml | Ipratropium Bromide | 0.25mg/ml | 3010200 |
| 6081 | Ipratropium bromide 20micrograms/dose breath actuated inhaler | Ipratropium bromide | 20microgram/1dose | 3010200 |
| 1409 | Ipratropium bromide 20micrograms/dose inhaler | Ipratropium bromide | 20microgram/1dose | 3010200 |
| 6522 | Ipratropium bromide 20micrograms/dose inhaler CFC free | Ipratropium bromide | 20microgram/1dose | 3010200 |
| 37791 | Ipratropium bromide 250microgram/ml | Ipratropium Bromide | 250microgram/ml | 3010200 |
| 23961 | Ipratropium bromide 250microgram/ml Inhalation vapour (Galen Ltd) | Ipratropium Bromide | 250microgram/ml | 3010200 |
| 68030 | Ipratropium bromide 250microgram/ml Nebuliser liquid (Approved Prescription Services Ltd) | Ipratropium bromide | 250microgram/1ml | 3010200 |
| 30229 | Ipratropium bromide 250microgram/ml Nebuliser liquid (Galen Ltd) | Ipratropium bromide | 250microgram/1ml | 3010200 |
| 40177 | Ipratropium bromide 250microgram/ml Nebuliser liquid (Hillcross Pharmaceuticals Ltd) | Ipratropium Bromide | 250microgram/ml | 3010200 |
| 6772 | Ipratropium bromide 250micrograms/1ml nebuliser liquid unit dose vials | Ipratropium bromide | 250microgram/1ml | 3010200 |
| 1411 | Ipratropium bromide 250micrograms/ml | Ipratropium Bromide | 250micrograms/ml | 3010200 |
| 8333 | Ipratropium bromide 40microgram inhalation powder capsules | Ipratropium bromide | 40microgram | 3010200 |
| 11779 | Ipratropium bromide 40microgram inhalation powder capsules with device | Ipratropium bromide | 40microgram | 3010200 |
| 4268 | Ipratropium bromide 40micrograms/dose inhaler | Ipratropium bromide | 40microgram/1dose | 3010200 |
| 6719 | Ipratropium bromide 500micrograms/2ml nebuliser liquid unit dose vials | Ipratropium bromide | 250microgram/1ml | 3010200 |
| 53174 | Ipratropium bromide 500micrograms/2ml nebuliser liquid unit dose vials (A A H Pharmaceuticals Ltd) | Ipratropium bromide | 250microgram/1ml | 3010200 |
| 23567 | Respontin 250micrograms/1ml Nebules (GlaxoSmithKline UK Ltd) | Ipratropium bromide | 250microgram/1ml | 3010200 |
| 18140 | Respontin 500micrograms/2ml Nebules (GlaxoSmithKline UK Ltd) | Ipratropium bromide | 250microgram/1ml | 3010200 |
| 18421 | Respontin nebules 250microgram/ml Nebuliser liquid (Glaxo Wellcome UK Ltd) | Ipratropium Bromide | 250microgram/ml | 3010200 |
| 1415 | Steri-neb ipratropium 250microgram/ml Nebuliser liquid (IVAX Pharmaceuticals UK Ltd) | Ipratropium Bromide | 250microgram/ml | 3010200 |
| 13757 | Tropiovent steripoule 250microgram/ml Nebuliser liquid (Ashbourne Pharmaceuticals Ltd) | Ipratropium Bromide | 250microgram/ml | 3010200 |

Supplementary Table 39: Oral LABA product codes

| **Product code** | **Product name** | **Drug substance name** | **Substance strength** | **BNF code** |
| --- | --- | --- | --- | --- |
| 7192 | Bambuterol 10mg tablets | Bambuterol hydrochloride | 10mg | 3010101 |
| 12144 | Bambuterol 20mg tablets | Bambuterol hydrochloride | 20mg | 3010101 |
| 13575 | Bambec 120mg tablets (AstraZeneca UK Ltd) | Bambuterol hydrochloride | 20mg | 3010101 |
| 14527 | Bambec 10mg tablets (AstraZeneca UK Ltd) | Bambuterol hydrochloride | 10mg | 3010101 |
| 31262 | Orciprenaline with bromhexine hcl mixture | Bromhexine/Orciprenaline Sulphate |  | 3010102 |
| 32812 | Numotac 10mg Tablet (3M Health Care Ltd) | Isoetarine Hydrochloride | 10mg | 3010101 |
| 2490 | Orciprenaline 10mg/5ml oral solution sugar free | Orciprenaline sulfate | 2mg/1ml | 0 |
| 7943 | Orciprenaline 20mg tablets | Orciprenaline sulfate | 20mg | 0 |
| 8012 | Exirel 15mg Capsule (3M Health Care Ltd) | Pirbuterol | 15mg | 3010101 |
| 8252 | Pirbuterol 15mg capsule | Pirbuterol | 15mg | 3010101 |
| 22661 | Pirbuterol 10mg capsule | Pirbuterol | 10mg | 3010101 |
| 23787 | Exirel 10mg Capsule (3M Health Care Ltd) | Pirbuterol | 10mg | 3010101 |
| 25821 | Exirel 7.5mg/5ml Oral solution (3M Health Care Ltd) | Pirbuterol | 7.5mg/5ml | 3010101 |
| 25829 | Pirbuterol 7.5mg/5ml oral solution | Pirbuterol | 7.5mg/5ml | 3010101 |
| 15075 | Bronchodil 20mg Tablet (Viatris Pharmaceuticals Ltd) | Reproterol Hydrochloride | 20mg | 3010101 |
| 25820 | Bronchodil 10mg/5ml Oral solution (Viatris Pharmaceuticals Ltd) | Reproterol Hydrochloride | 10mg/5ml | 3010101 |
| 36677 | Reproterol 10mg/5ml oral solution | Reproterol Hydrochloride | 10mg/5ml | 3010101 |
| 8080 | Ritodrine 10mg tablets | Ritodrine hydrochloride | 10mg | 0 |
| 42497 | Salbutamol 8mg tablet | Salbutamol | 8mg | 3010101 |
| 65376 | Salbutamol 2mg/5ml oral solution sugar free (Pinewood Healthcare) | Salbutamol sulfate | 400microgram/1ml | 3010101 |
| 282 | Salbutamol 2mg/5ml oral solution sugar free | Salbutamol sulphate | 400microgram/1ml | 3010101 |
| 696 | Salbutamol 8mg modified-release capsules | Salbutamol sulphate | 8mg | 3010101 |
| 856 | Ventolin 2mg/5ml syrup (GlaxoSmithKline UK Ltd) | Salbutamol sulphate | 400microgram/1ml | 3010101 |
| 860 | Salbutamol 4mg tablets | Salbutamol sulphate | 4mg | 3010101 |
| 881 | Salbutamol 2mg tablets | Salbutamol sulphate | 2mg | 3010101 |
| 987 | Ventolin 4mg Tablet (Allen & Hanburys Ltd) | Salbutamol sulphate | 4mg | 07010300/03010101 |
| 1635 | Salbuvent 2mg/5ml Oral solution (Pharmacia Ltd) | Salbutamol sulphate | 400microgram/1ml | 07010300/03010101 |
| 1960 | Volmax 8mg modified-release tablets (GlaxoSmithKline UK Ltd) | Salbutamol sulphate | 8mg | 0 |
| 1961 | Volmax 4mg modified-release tablets (GlaxoSmithKline UK Ltd) | Salbutamol sulphate | 4mg | 0 |
| 2869 | Salbutamol 8mg modified-release tablets | Salbutamol sulphate | 8mg | 0 |
| 3254 | Salbulin 4mg Tablet (3M Health Care Ltd) | Salbutamol sulphate | 4mg | 07010300/03010101 |
| 3994 | Salbutamol 4mg modified-release tablets | Salbutamol sulphate | 4mg | 0 |
| 4055 | Salbulin 2mg/5ml Oral solution (3M Health Care Ltd) | Salbutamol sulphate | 400microgram/1ml | 07010300/03010101 |
| 4171 | Ventolin 2mg Tablet (Allen & Hanburys Ltd) | Salbutamol sulphate | 2mg | 07010300/03010101 |
| 9384 | Salbutamol 4mg modified-release capsules | Salbutamol sulphate | 4mg | 3010101 |
| 10458 | Ventolin cr 4mg Tablet (Allen & Hanburys Ltd) | Salbutamol sulphate | 4mg | 3010101 |
| 12042 | Ventolin cr 8mg Tablet (Allen & Hanburys Ltd) | Salbutamol sulphate | 8mg | 3010101 |
| 17696 | Ventmax SR 4mg capsules (Chiesi Ltd) | Salbutamol sulphate | 4mg | 3010101 |
| 18622 | Salbulin 2mg Tablet (3M Health Care Ltd) | Salbutamol sulphate | 2mg | 07010300/03010101 |
| 20838 | Salbuvent 2mg Tablet (Pharmacia Ltd) | Salbutamol sulphate | 2mg | 03010101/07010300 |
| 21102 | Salbutamol 2mg/5ml Oral solution (Lagap) | Salbutamol sulphate | 400microgram/1ml | 03010101/07010300 |
| 22313 | Ventmax SR 8mg capsules (Chiesi Ltd) | Salbutamol sulphate | 8mg | 3010101 |
| 26873 | Cobutolin 2mg Tablet (Actavis UK Ltd) | Salbutamol sulphate | 2mg | 07010300/03010101 |
| 28881 | Salbutamol 2mg/5ml oral solution sugar free (A A H Pharmaceuticals Ltd) | Salbutamol sulphate | 400microgram/1ml | 3010101 |
| 29267 | Salbuvent 4mg Tablet (Pharmacia Ltd) | Salbutamol sulphate | 4mg | 03010101/07010300 |
| 31845 | Salapin 2mg/5ml syrup (Pinewood Healthcare) | Salbutamol sulphate | 400microgram/1ml | 3010101 |
| 32102 | Salbutamol 4mg tablets (A A H Pharmaceuticals Ltd) | Salbutamol sulphate | 4mg | 3010101 |
| 34618 | Salbutamol 2mg tablets (Actavis UK Ltd) | Salbutamol sulphate | 2mg | 3010101 |
| 34938 | Salbutamol 4mg tablets (Actavis UK Ltd) | Salbutamol sulphate | 4mg | 3010101 |
| 41548 | Salbutamol 2mg tablets (Approved Prescription Services Ltd) | Salbutamol sulphate | 2mg | 3010101 |
| 41549 | Salbutamol 2mg Tablet (C P Pharmaceuticals Ltd) | Salbutamol sulphate | 2mg | 03010101/07010300 |
| 41691 | Salbutamol 2mg/5ml oral solution sugar free (Sandoz Ltd) | Salbutamol sulphate | 400microgram/1ml | 3010101 |
| 3534 | Bricanyl 5mg tablets (AstraZeneca UK Ltd) | Terbutaline sulphate | 5mg | 03010101/07010300 |
| 3584 | Bricanyl 1.5mg/5ml syrup (AstraZeneca UK Ltd) | Terbutaline sulphate | 300microgram/1ml | 07010300/03010101 |
| 4541 | Bricanyl SA 7.5mg tablets (AstraZeneca UK Ltd) | Terbutaline sulphate | 7.5mg | 0 |
| 7953 | Terbutaline 1.5mg/5ml oral solution sugar free | Terbutaline sulphate | 300microgram/1ml | 03010101/07010300 |
| 8522 | Terbutaline 7.5mg modified-release tablets | Terbutaline sulphate | 7.5mg | 0 |
| 10825 | Terbutaline 5mg tablets | Terbutaline sulphate | 5mg | 07010300/03010101 |
| 17874 | Monovent 1.5mg/5ml Oral solution (Lagap) | Terbutaline sulphate | 300microgram/1ml | 07010300/03010101 |
| 38419 | Terbutaline 1.5mg/5ml oral solution sugar free (A A H Pharmaceuticals Ltd) | Terbutaline sulphate | 300microgram/1ml | 0 |
| 41832 | Monovent 1.5mg/5ml syrup (Sandoz Ltd) | Terbutaline sulphate | 300microgram/1ml | 0 |
| 42867 | Terbutaline 1.5mg/5ml Oral solution (Sandoz Ltd) | Terbutaline sulphate | 300microgram/1ml | 07010300/03010101 |
| 15483 | Bricanyl Oral solution (AstraZeneca UK Ltd) | Terbutaline Sulphate/Guaifenesin |  | 3010101 |
| 17875 | Terbutaline with guafenesin expectorant | Terbutaline Sulphate/Guaifenesin |  | 3010101 |
| 26987 | Bricanyl Tablet (AstraZeneca UK Ltd) | Terbutaline Sulphate/Guaifenesin |  | 3010101 |
| 19799 | Tulobuterol 2mg | Tulobuterol | 2mg | 3010101 |
| 22663 | Respacal 2mg Tablet (UCB Pharma Ltd) | Tulobuterol | 2mg | 3010101 |
| 26829 | Brelomax 2mg Tablet (Abbott Laboratories Ltd) | Tulobuterol | 2mg | 3010101 |
| 2395 | SALBUTAMOL 2 MG/5ML SYR |  |  | 0 |
| 12463 | PIRBUTEROL 15 MG TAB |  |  | 0 |

Supplementary Table 40: Inhaled LABA product codes

| **Product code** | **Product name** | **Drug substance name** | **Substance strength** | **BNF code** |
| --- | --- | --- | --- | --- |
| 1794 | Berotec 100microgram/actuation Inhalation powder (Boehringer Ingelheim Ltd) | Fenoterol Hydrobromide | 100microgram/actuation | 3010101 |
| 4842 | Fenoterol 100microgram/actuation inhaler | Fenoterol Hydrobromide | 100microgram/actuation | 3010101 |
| 5185 | Fenoterol 200micrograms/dose inhaler | Fenoterol hydrobromide | 200microgram/1dose | 0 |
| 13365 | Berotec 5mg/ml Nebuliser liquid (Boehringer Ingelheim Ltd) | Fenoterol Hydrobromide | 5mg/ml | 3010101 |
| 3786 | Fenoterol 100micrograms/dose / Ipratropium 40micrograms/dose inhaler | Fenoterol hydrobromide/Ipratropium bromide | 100microgram/1dose + 40microgram/1dose | 0 |
| 18299 | Fenoterol 1.25mg/4ml / Ipratropium 500micrograms/4ml nebuliser liquid unit dose vials | Fenoterol hydrobromide/Ipratropium bromide | 312.5microgram/1ml + 125microgram/1ml | 3010400 |
| 1974 | Oxis 12 Turbohaler (AstraZeneca UK Ltd) | Formoterol fumarate dihydrate |  |  |
| 1975 | Oxis 6 Turbohaler (AstraZeneca UK Ltd) | Formoterol fumarate dihydrate |  |  |
| 6526 | Formoterol 12microgram inhalation powder capsules with device | Formoterol fumarate dihydrate | 12microgram | 3010101 |
| 7133 | Formoterol 12micrograms/dose dry powder inhaler | Formoterol fumarate dihydrate | 12microgram/1dose | 3010101 |
| 9711 | Formoterol 6micrograms/dose dry powder inhaler | Formoterol fumarate dihydrate | 6microgram/1dose | 3010101 |
| 10968 | Foradil 12microgram inhalation powder capsules with device (Novartis Pharmaceuticals UK Ltd) | Formoterol fumarate dihydrate | 12microgram | 3010101 |
| 14306 | Formoterol 12micrograms/dose inhaler CFC free | Formoterol Fumarate Dihydrate | 12micrograms/actuation | 3010101 |
| 25784 | Atimos Modulite 12micrograms/dose inhaler (Chiesi Ltd) | Formoterol fumarate dihydrate | 12microgram/1dose | 3010101 |
| 35725 | Formoterol Easyhaler 12micrograms/dose dry powder inhaler (Orion Pharma (UK) Ltd) | Formoterol fumarate dihydrate | 12microgram/1dose | 3010101 |
| 56482 | Oxis 12 Turbohaler (Waymade Healthcare Plc) | Formoterol fumarate dihydrate |  |  |
| 57558 | Oxis 6 Turbohaler (Lexon (UK) Ltd) | Formoterol fumarate dihydrate | 6microgram/1dose | 3010101 |
| 66547 | Oxis 12 Turbohaler (DE Pharmaceuticals) | Formoterol fumarate dihydrate | 12microgram/1dose | 3010101 |
| 67238 | Foradil 12microgram inhalation powder capsules with device (Sigma Pharmaceuticals Plc) | Formoterol fumarate dihydrate | 12microgram | 3010101 |
| 62838 | Aclidinium bromide 396micrograms/dose / Formoterol 11.8micrograms/dose dry powder inhaler | Formoterol fumarate dihydrate/Aclidinium bromide | 11.8microgram/1dose + 396microgram/1dose | 3010400 |
| 6796 | Budesonide 200micrograms/dose / Formoterol 6micrograms/dose dry powder inhaler | Formoterol fumarate dihydrate/Budesonide | 6microgram/1dose + 200microgram/1dose | 03010100/03020000 |
| 62739 | Indacaterol 85micrograms/dose / Glycopyrronium bromide 54micrograms/dose inhalation powder capsules with device | Glycopyrronium bromide/Indacaterol maleate | 54microgram/1dose + 85microgram/1dose | 3010400 |
| 43738 | Indacaterol 150 microgram inhalation powder capsules with device | Indacaterol Maleate | 150micrograms | 3010101 |
| 43893 | Onbrez Breezhaler 150microgram inhalation powder capsules with devide (Novartis Pharmaceuticals UK Ltd) | Indacaterol Maleate | 150micrograms | 3010101 |
| 44064 | Onbrez Breezhaler 300microgram inhalation powder capsules with devide (Novartis Pharmaceuticals UK Ltd) | Indacaterol Maleate | 300micrograms | 3010101 |
| 45610 | Indacaterol 300microgram inhalation powder capsules with device | Indacaterol Maleate | 300micrograms | 3010101 |
| 62662 | Olodaterol 2.5micrograms/dose solution for inhaltion cartridge with device CFC free | Olodaterol hydrochloride | 2.5micrograms/1dose | 3010101 |
| 65431 | Striverdi Respimat 2.5micrpgrams/dose solution for inhalation cartridge with devide (Boehringer Ingelheim Ltd) | Olodaterol hydrochloride | 2.5micrograms/1dose | 3010101 |
| 461 | Orciprenaline 750micrograms/inhalation inhaler | Orciprenaline Sulphate | 750micrograms/inhalation | 3010102 |
| 8151 | Orciprenaline 750micrograms/inhalation Aerosol refill | Orciprenaline Sulphate | 750micrograms/inhalation | 3010102 |
| 32283 | Orciprenaline 0.5mg/ml Injection | Orciprenaline Sulphate | 0.5mg/ml | 3010102 |
| 12563 | Exirel Inhalation powder (3M Health Care Ltd) | Pirbuterol |  | 3010101 |
| 16236 | Pirbuterol acetate inhaler | Pirbuterol |  | 3010101 |
| 12486 | Bronchodil 500microgram/dose Inhalation powder (Viatris Pharmaceuticals Ltd) | Reproterol Hydrochloride | 500microgram/dose | 3010101 |
| 15165 | Reproterol 500micrograms/dose inhaler | Reproterol Hydrochloride | 500micrograms/dose | 3010101 |
| 22790 | Reproterol 10mg/ml respirator solution | Reproterol Hydrochloride | 10mg/ml | 3010101 |
| 3758 | Pulmadil Inhalation powder (3M Health Care Ltd) | Rimiterol Hydrobromide |  | 3010101 |
| 8572 | Rimiterol inhaler | Rimiterol Hydrobromide |  | 3010101 |
| 10858 | Pulmadil auto Inhalation powder (3M Health Care Ltd) | Rimiterol Hydrobromide |  | 3010101 |
| 465 | Salmeterol 25micrograms/dose inhaler | Salmeterol xinafoate | 25microgram/1dose | 0 |
| 549 | Serevent 25micrograms/dose inhaler (GlaxoSmithKline UK Ltd) | Salmeterol xinafoate | 25microgram/1dose | 0 |
| 719 | Salmeterol 50micrograms/dose dry powder inhaler | Salmeterol xinafoate | 50microgram/1dose | 3010101 |
| 910 | Serevent diskhaler 50microgram Inhalation powder (Glaxo Wellcome UK Ltd) | Salmeterol Xinafoate | 50microgram | 3010101 |
| 2224 | Serevent 50micrograms/dose Accuhaler (GlaxoSmithKline UK Ltd) | Salmeterol xinafoate | 50microgram/1dose | 3010101 |
| 3297 | Salmeterol 50micrograms disc | Salmeterol Xinafoate | 50micrograms | 3010101 |
| 5558 | Salmeterol 50micrograms with fluticasone 500micrograms CFC free inhaler | Salmeterol Xinafoate | 50micrograms+ 500micrograms/inhalation | 03020000/03010101 |
| 5864 | Salmeterol 25micrograms with fluticasone 250micrograms CFC free inhaler | Salmeterol Xinafoate | 25micrograms + 250micrograms/actuation | 03020000/03010101 |
| 5942 | Salmeterol 50micrograms with fluticasone 250micrograms CFC free inhaler | Salmeterol Xinafoate | 50micrograms + 250micrograms/inhalation | 03010101/03020000 |
| 6569 | Salmeterol 25micrograms with fluticasone 125micrograms CFC free inhaler | Salmeterol Xinafoate | 25micrograms + 125micrograms/actuation | 03010101/03020000 |
| 6616 | Salmeterol 25micrograms with fluticasone 50micrograms CFC free inhaler | Salmeterol Xinafoate | 25micrograms + 50micrograms/actuation | 03020000/03010101 |
| 6938 | Salmeterol 50micrograms with fluticasone 100micrograms dry powder inhaler | Salmeterol Xinafoate | 50micrograms + 100micrograms/inhalation | 03010101/03020000 |
| 7268 | Serevent 25micrograms/dose Evohaler (GlaxoSmithKline UK Ltd) | Salmeterol Xinafoate | 25micrograms/actuation | 3010101 |
| 7270 | Salmeterol 25micrograms/dose inhaler CFC free | Salmeterol Xinafoate | 25micrograms/actuation | 3010101 |
| 35165 | Serevent 50microgram disks with Diskhaler (GlaxoSmithKline UK Ltd) | Salmeterol xinafoate | 50microgram | 3010101 |
| 35503 | Salmeterol 50microgram inhalation powder blisters | Salmeterol xinafoate | 50microgram | 3010101 |
| 35542 | Salmeterol 50microgram inhalation powder blisters with device | Salmeterol xinafoate | 50microgram | 3010101 |
| 35825 | Serevent 50microgram disks (GlaxoSmithKline UK Ltd) | Salmeterol xinafoate | 50microgram | 3010101 |
| 47638 | Neovent 25micrograms/dose inhaler CFC free (Fannin UK Ltd) | Salmeterol xinafoate | 25microgram/1dose | 3010101 |
| 50051 | Serevent 25micrograms/dose Evohaler (Waymade Healthcare Plc) | Salmeterol xinafoate | 25microgram/1dose | 3010101 |
| 54742 | Salmeterol 25micrograms/dose inhaler CFC free (A A H Pharmaceuticals Ltd) | Salmeterol xinafoate | 25microgram/1dose | 3010101 |
| 56478 | Serevent 50micrograms/dose Accuhaler (Doncaster Pharmaceuticals Ltd) | Salmeterol xinafoate |  | 3010101 |
| 57544 | Serevent 50micrograms/dose Accuhaler (Waymade Healthcare Plc) | Salmeterol xinafoate | 50microgram/1dose | 3010101 |
| 57694 | Vertine 25micrograms/dose inhaler CFC free (Teva UK Ltd) | Salmeterol xinafoate | 25microgram/1dose | 3010101 |
| 67823 | Salmeterol 50microgram Diskhaler (Dowelhurst Ltd) | Salmeterol xinafoate | 50microgram | 3010101 |
| 68260 | Serevent 50micrograms/dose Accuhaler (Mawdsley-Brooks & Company Ltd) | Salmeterol xinafoate | 50microgram/1dose | 3010101 |
| 8339 | FENOTEROL HYDROBROMIDE COMPLETE UNIT INH |  |  | 0 |
| 15413 | ORCIPRENALINE SULPHATE 5% SOL |  |  | 0 |
| 15441 | FENOTEROL HYDROBROMIDE .5% SOL |  |  | 0 |
| 67800 | Serevent 25micrograms/dose Evohaler (Lexon (UK) Ltd) |  |  | 3010101 |
| 67800 | Serevent 25micrograms/dose Evohaler (Lexon (UK) Ltd) |  |  | 3010101 |
| 68483 | Soltel 25micrograms/dose inhaler CFC free (Kent Pharmaceuticals Ltd) |  |  | 3010101 |

Supplementary Table 41: Combination ICS/LABA product codes

| **Product code** | **Product name** | **Drug substance name** | **Substance strength** | **mcg ICS per dose** | **BNF code** |
| --- | --- | --- | --- | --- | --- |
| 3556 | Beclometasone 50micrograms with salbutamol 100micrograms/inhalation inhaler | Beclometasone Dipropionate/Salbutamol | 50micrograms + 100micrograms/inhalation |  | 03010101/03020000 |
| 19121 | Beclometasone 100micrograms with Salbutamol 200micrograms inhalation capsules | Beclometasone Dipropionate/Salbutamol | 100micrograms + 200micrograms |  | 03010101/03020000 |
| 19376 | Beclometasone 200micrograms with Salbutamol 400micrograms inhalation capsules | Beclometasone Dipropionate/Salbutamol | 200micrograms + 400micrograms |  | 03010101/03020000 |
| 61644 | Fostair NEXThaler 100micrograms/dose / 6micrograms/dose dry powder inhaler (Chiesi Ltd) | Beclomethasone dipropionate/formoterol fumerate dihydrate | 100microgram/1dose + 6microgram/1dose | 100 |  |
| 65658 | Fostair NEXThaler 200micrograms/dose / 6micrograms/dose dry powder inhaler (Chiesi Ltd) | Beclomethasone dipropionate/formoterol fumerate dihydrate | 200microgram/1dose + 6microgram/1dose | 200 |  |
| 31262 | Orciprenaline with bromhexine hcl mixture | Bromhexine/Orciprenaline Sulphate |  |  | 3010102 |
| 6166 | DuoResp Spiromax 320micrograms/dose/9micrograms/dose dry powder inhaler (Teva UK Ltd) | Budesonide/Formoterol fumarate dihydrate | 320microgram/1dose + 9microgram/1dose | 320 | 03020000/03010100 |
| 6325 | Symbicort 200/6 Turbohaler (AstraZeneca UK Ltd) | Budesonide/Formoterol fumarate dihydrate | 200microgram/1dose + 6microgram/1dose | 200 | 03010100/03020000 |
| 6746 | Budesonide 400micrograms/dose / Formoterol 12micrograms/dose dry powder inhaler | Budesonide/Formoterol fumarate dihydrate | 400microgram/1dose + 12microgram/1dose | 400 | 03020000/03010100 |
| 6780 | Symbicort 400/12 Turbohaler (AstraZeneca UK Ltd) | Budesonide/Formoterol fumarate dihydrate | 400microgram/1dose + 12microgram/1dose | 400 | 03010100/03020000 |
| 6796 | Budesonide 200micrograms/dose / Formoterol 6micrograms/dose dry powder inhaler | Budesonide/Formoterol fumarate dihydrate | 200microgram/1dose + 6microgram/1dose | 200 | 03020000/03010100 |
| 7013 | Symbicort 100/6 Turbohaler (AstraZeneca UK Ltd) | Budesonide/Formoterol fumarate dihydrate | 100microgram/1dose + 6microgram/1dose | 100 | 03010100/03020000 |
| 10218 | Budesonide 100micrograms/dose / Formoterol 6micrograms/dose dry powder inhaler | Budesonide/Formoterol fumarate dihydrate | 100microgram/1dose + 6microgram/1dose | 100 | 03020000/03010100 |
| 49114 | Symbicort 100/6 Turbohaler (Sigma Pharmaceuticals Plc) | Budesonide/Formoterol fumarate dihydrate | 100microgram/1dose + 6microgram/1dose | 100 | 03010100/03020000 |
| 50739 | Symbicort 400/12 Turbohaler (Mawdsley-Brooks & Company Ltd) | Budesonide/Formoterol fumarate dihydrate | 400microgram/1dose + 12microgram/1dose | 400 | 03020000/03010100 |
| 50945 | Symbicort 100/6 Turbohaler (Mawdsley-Brooks & Company Ltd) | Budesonide/Formoterol fumarate dihydrate | 100microgram/1dose + 6microgram/1dose | 100 | 03010100/03020000 |
| 51570 | Symbicort 200/6 Turbohaler (Doncaster Pharmaceuticals Ltd) | Budesonide/Formoterol fumarate dihydrate | 200microgram/1dose + 6microgram/1dose | 200 | 03020000/03010100 |
| 51759 | Symbicort 200/6 Turbohaler (Mawdsley-Brooks & Company Ltd) | Budesonide/Formoterol fumarate dihydrate | 200microgram/1dose + 6microgram/1dose | 200 | 03010100/03020000 |
| 53237 | Symbicort 400/12 Turbohaler (Doncaster Pharmaceuticals Ltd) | Budesonide/Formoterol fumarate dihydrate | 400microgram/1dose + 12microgram/1dose | 400 | 03010100/03020000 |
| 53491 | Symbicort 200/6 Turbohaler (Sigma Pharmaceuticals Plc) | Budesonide/Formoterol fumarate dihydrate | 200microgram/1dose + 6microgram/1dose | 200 | 03010100/03020000 |
| 61666 | DuoResp Spiromax 320micrograms/dose / 9micrograms/dose dry powder inhaler (Teva UK Ltd) | Budesonide/Formoterol fumarate dihydrate | 400microgram/1dose + 12microgram/1dose |  | 03010100/03020000 |
| 61782 | DuoResp Spiromax 160micrograms/dose/4.5micrograms/dose dry powder inhaler (Teva UK Ltd) | Budesonide/Formoterol fumarate dihydrate | 160microgram/1dose + 4.5microgram/1dose | 160 | 03020000/03010100 |
| 3786 | Fenoterol 100micrograms/dose / Ipratropium 40micrograms/dose inhaler | Fenoterol hydrobromide/Ipratropium bromide | 100microgram/1dose + 40microgram/1dose |  | 0 |
| 18299 | Fenoterol 1.25mg/4ml / Ipratropium 500micrograms/4ml nebuliser liquid unit dose vials | Fenoterol hydrobromide/Ipratropium bromide | 312.5microgram/1ml + 125microgram/1ml |  | 3010400 |
| 59327 | Relvar Ellipta 92 micrograms/dose 22micrograms/dose dry powder inhaler (GlaxoSmithKline UK Ltd) | fluticasone furoate , vilanterol trifenatate | 92microgram/1dose + 22microgram/1dose | 92 | 3010400 |
| 59573 | Relvar Ellipta 184 micrograms/dose 22micrograms/dose dry powder inhaler (GlaxoSmithKline UK Ltd) | fluticasone furoate , vilanterol trifenatate | 184microgram/1dose + 22microgram/1dose | 184 | 3010400 |
| 48666 | Flutiform 250micrograms/dose / 10micrograms/dose inhaler (Napp Pharmaceuticals Ltd) | Fluticasone propionate/Formoterol fumarate dihydrate | 250microgram/1dose + 10microgram/1dose | 250 |  |
| 50036 | Flutiform 125micrograms/dose / 5micrograms/dose inhaler (Napp Pharmaceuticals Ltd) | Fluticasone propionate/Formoterol fumarate dihydrate | 125microgram/1dose + 25microgram/1dose | 125 |  |
| 50689 | Flutiform 50micrograms/dose / 5micrograms/dose inhaler (Napp Pharmaceuticals Ltd) | Fluticasone propionate/Formoterol fumarate dihydrate | 50microgram/1dose + 5microgram/1dose | 50 | 3020000 |
| 51270 | Fluticasone 50micrograms/dose / Formoterol 5micrograms/dose inhaler CFC free | Fluticasone propionate/Formoterol fumarate dihydrate | 50microgram/1dose + 5microgram/1dose | 50 | 3020000 |
| 64638 | Flutiform 125micrograms/dose / 5micrograms/dose inhaler (Waymade Healthcare Plc) | Fluticasone propionate/Formoterol fumarate dihydrate | 125microgram/1dose + 25microgram/1dose | 125 |  |
| 66448 | Flutiform 250micrograms/dose / 10micrograms/dose inhaler (Waymade Healthcare Plc) | Fluticasone propionate/Formoterol fumarate dihydrate | 250microgram/1dose + 10microgram/1dose | 250 |  |
| 68175 | Flutiform 50micrograms/dose / 5micrograms/dose inhaler (Waymade Healthcare Plc) | Fluticasone propionate/Formoterol fumarate dihydrate | 50microgram/1dose + 5microgram/1dose | 50 |  |
| 638 | Seretide 250 Accuhaler (GlaxoSmithKline UK Ltd) | Fluticasone propionate/Salmeterol xinafoate | 250microgram/1dose + 50microgram/1dose | 250 | 03020000/03010101 |
| 665 | Seretide 100 Accuhaler (GlaxoSmithKline UK Ltd) | Fluticasone propionate/Salmeterol xinafoate | 100microgram/1dose + 50microgram/1dose | 100 | 03010101/03020000 |
| 3666 | Seretide 500 Accuhaler (GlaxoSmithKline UK Ltd) | Fluticasone propionate/Salmeterol xinafoate | 500microgram/1dose + 50microgram/1dose | 500 | 03010101/03020000 |
| 5143 | Seretide 50 Evohaler (GlaxoSmithKline UK Ltd) | Fluticasone propionate/Salmeterol xinafoate | 50microgram/1dose + 25microgram/1dose | 50 | 03020000/03010101 |
| 5161 | Seretide 125 Evohaler (GlaxoSmithKline UK Ltd) | Fluticasone propionate/Salmeterol xinafoate | 125microgram/1dose + 25microgram/1dose | 125 | 03010101/03020000 |
| 5172 | Seretide 250 Evohaler (GlaxoSmithKline UK Ltd) | Fluticasone propionate/Salmeterol xinafoate | 250microgram/1dose + 25microgram/1dose | 250 | 03020000/03010101 |
| 11410 | Fluticasone 500micrograms/dose / Salmeterol 50micrograms/dose dry powder inhaler | Fluticasone propionate/Salmeterol xinafoate | 500microgram/1dose + 50microgram/1dose | 500 | 03010101/03020000 |
| 11588 | Fluticasone 125micrograms/dose / Salmeterol 25micrograms/dose inhaler CFC free | Fluticasone propionate/Salmeterol xinafoate | 125microgram/1dose + 25microgram/1dose | 125 | 03010101/03020000 |
| 11618 | Fluticasone 250micrograms/dose / Salmeterol 25micrograms/dose inhaler CFC free | Fluticasone propionate/Salmeterol xinafoate | 250microgram/1dose + 25microgram/1dose | 250 | 03010101/03020000 |
| 12994 | Fluticasone 50micrograms/dose / Salmeterol 25micrograms/dose inhaler CFC free | Fluticasone propionate/Salmeterol xinafoate | 50microgram/1dose + 25microgram/1dose | 50 | 03010101/03020000 |
| 13040 | Fluticasone 250micrograms/dose / Salmeterol 50micrograms/dose dry powder inhaler | Fluticasone propionate/Salmeterol xinafoate | 250microgram/1dose + 50microgram/1dose | 250 | 03010101/03020000 |
| 13273 | Fluticasone 100micrograms/dose / Salmeterol 50micrograms/dose dry powder inhaler | Fluticasone propionate/Salmeterol xinafoate | 100microgram/1dose + 50microgram/1dose | 100 | 03020000/03010101 |
| 48739 | Seretide 250 Evohaler (Doncaster Pharmaceuticals Ltd) | Fluticasone propionate/Salmeterol xinafoate | 250microgram/1dose + 25microgram/1dose | 250 | 03010101/03020000 |
| 49000 | Seretide 250 Evohaler (Waymade Healthcare Plc) | Fluticasone propionate/Salmeterol xinafoate | 250microgram/1dose + 25microgram/1dose | 250 | 03010101/03020000 |
| 50560 | Seretide 250 Accuhaler (Sigma Pharmaceuticals Plc) | Fluticasone propionate/Salmeterol xinafoate | 250microgram/1dose + 50microgram/1dose | 250 | 03010101/03020000 |
| 50886 | Seretide 250 Evohaler (Stephar (U.K.) Ltd) | Fluticasone propionate/Salmeterol xinafoate | 250microgram/1dose + 25microgram/1dose | 250 | 03020000/03010101 |
| 51027 | Seretide 125 Evohaler (Doncaster Pharmaceuticals Ltd) | Fluticasone propionate/Salmeterol xinafoate | 125microgram/1dose + 25microgram/1dose | 125 | 03010101/03020000 |
| 51151 | Seretide 125 Evohaler (Lexon (UK) Ltd) | Fluticasone propionate/Salmeterol xinafoate | 125microgram/1dose + 25microgram/1dose | 125 | 03020000/03010101 |
| 51394 | Seretide 500 Accuhaler (Waymade Healthcare Plc) | Fluticasone propionate/Salmeterol xinafoate | 500microgram/1dose + 50microgram/1dose | 500 | 03010101/03020000 |
| 51593 | Seretide 500 Accuhaler (Doncaster Pharmaceuticals Ltd) | Fluticasone propionate/Salmeterol xinafoate | 500microgram/1dose + 50microgram/1dose | 500 | 03020000/03010101 |
| 51861 | Seretide 500 Accuhaler (Mawdsley-Brooks & Company Ltd) | Fluticasone propionate/Salmeterol xinafoate | 500microgram/1dose + 50microgram/1dose | 500 | 03020000/03010101 |
| 51909 | Seretide 250 Evohaler (Necessity Supplies Ltd) | Fluticasone propionate/Salmeterol xinafoate | 250microgram/1dose + 25microgram/1dose | 250 | 03010101/03020000 |
| 53230 | Seretide 250 Accuhaler (Doncaster Pharmaceuticals Ltd) | Fluticasone propionate/Salmeterol xinafoate | 250microgram/1dose + 50microgram/1dose | 250 | 03020000/03010101 |
| 53283 | Seretide 100 Accuhaler (Waymade Healthcare Plc) | Fluticasone propionate/Salmeterol xinafoate | 100microgram/1dose + 50microgram/1dose | 100 | 03020000/03010101 |
| 55677 | Seretide 500 Accuhaler (Lexon (UK) Ltd) | Fluticasone propionate/Salmeterol xinafoate | 500microgram/1dose + 50microgram/1dose | 500 | 03020000/03010101 |
| 62126 | Seretide 100 Accuhaler (DE Pharmaceuticals) | Fluticasone propionate/Salmeterol xinafoate | 100microgram/1dose + 50microgram/1dose |  | 03010101/03020000 |
| 63252 | Seretide 250 Evohaler (Lexon (UK) Ltd) | Fluticasone propionate/Salmeterol xinafoate | 250microgram/1dose + 25microgram/1dose |  | 03010101/03020000 |
| 64372 | Sirdupla 25micrograms/dose / 125micrograms/dose inhaler (Mylan Ltd) | Fluticasone propionate/Salmeterol xinafoate | 125microgram/1dose + 25microgram/1dose | 125 |  |
| 64373 | Sirdupla 25micrograms/dose / 250micrograms/dose inhaler (Mylan Ltd) | Fluticasone propionate/Salmeterol xinafoate | 250microgram/1dose + 25microgram/1dose | 250 |  |
| 65117 | Seretide 125 Evohaler (Mawdsley-Brooks & Company Ltd) | Fluticasone propionate/Salmeterol xinafoate | 125microgram/1dose + 25microgram/1dose |  | 03010101/03020000 |
| 65677 | AirFluSal Forspiro 50micrograms/dose / 500micrograms/dose dry powder inhaler | Fluticasone propionate/Salmeterol xinafoate | 500microgram/1dose + 50microgram/1dose | 500 |  |
| 66453 | Sirdupla 25micrograms/dose / 250micrograms/dose inhaler (Waymade Healthcare Plc) | Fluticasone propionate/Salmeterol xinafoate | 250microgram/1dose + 25microgram/1dose | 250 |  |
| 67055 | Fluticasone 250micrograms/dose / Salmeterol 25micrograms/dose inhaler CFC free (A A H Pharmaceuticals Ltd) | Fluticasone propionate/Salmeterol xinafoate | 250microgram/1dose + 25microgram/1dose |  | 03010101/03020000 |
| 67101 | Fluticasone propionate 500micrograms/dose / Salmeterol 50micrograms/dose dry powder inhaler (A A H Pharmaceuticals Ltd) | Fluticasone propionate/Salmeterol xinafoate | 500microgram/1dose + 50microgram/1dose |  | 03010101/03020000 |
| 68453 | Seretide 125 Evohaler (Waymade Healthcare Plc) | Fluticasone propionate/Salmeterol xinafoate | 125microgram/1dose + 25microgram/1dose |  | 03010101/03020000 |
| 68495 | Seretide 500 Accuhaler (Necessity Supplies Ltd) | Fluticasone propionate/Salmeterol xinafoate | 500microgram/1dose + 50microgram/1dose |  | 03010101/03020000 |
| 68983 | Aerivio Spiromax 50micrograms/dose / 500micrograms/dose dry powder inhaler (Teva UK Ltd) | Fluticasone propionate/Salmeterol xinafoate | 500microgram/1dose + 50microgram/1dose | 500 |  |
| 37432 | Fostair 100micrograms/dose/6micrograms/dose inhaler (Chiesi Ltd) | Formoterol fumarate dihydrate/Beclometasone dipropionate | 6microgram/1dose + 100microgram/1dose | 100 | 3020000 |
| 37470 | Beclometasone 100micrograms/dose / Formoterol 6micrograms/dose inhaler CFC free | Formoterol fumarate dihydrate/Beclometasone dipropionate | 6microgram/1dose + 100microgram/1dose | 100 | 3020000 |
| 65596 | Fostair 200micrograms/dose / 6micrograms/dose inhaler (Chiesi Ltd) | Formoterol fumarate dihydrate/Beclometasone dipropionate | 6microgram/1dose + 200microgram/1dose | 200 |  |
| 67677 | Symbicort 200micrograms/dose / 6micrograms/dose pressurised inhaler (AstraZeneca UK Ltd) | Formoterol fumarate dihydrate/Budesonide | 6microgram/1dose + 200microgram/1dose |  | 03010100/03020000 |
| 68034 | Symbicort 200/6 Turbohaler (Necessity Supplies Ltd) | Formoterol fumarate dihydrate/Budesonide | 6microgram/1dose + 200microgram/1dose |  | 03010100/03020000 |
| 9270 | Ipratropium bromide with fenoterol hydrobromide 500micrograms + 1.25mg/4ml | Ipratropium Bromide/Fenoterol Hydrobromide | 500micrograms + 1.25mg/4ml |  | 03010101/03010400 |
| 12808 | Fenoterol 100micrograms/dose / Ipratropium bromide 40micrograms/dose breath actuated inhaler | Ipratropium bromide/Fenoterol hydrobromide | 40microgram/1dose + 100microgram/1dose |  | 0 |
| 26616 | Ipratropium bromide with fenoterol hydrobromide 0micrograms + 100micrograms/actuation | Ipratropium Bromide/Fenoterol Hydrobromide | 0micrograms + 100micrograms/actuation |  | 03010101/03010400 |
| 27505 | Ipratropium bromide with fenoterol hydrobromide 40micrograms + 100micrograms/actuation | Ipratropium Bromide/Fenoterol Hydrobromide | 40micrograms + 100micrograms/actuation |  | 03010101/03010400 |
| 48410 | Salbutamol 2.5mg/2.5ml / Ipratropium bromide 500micrograms/2.5ml nebuliser liquid ampoules | Ipratropium bromide/Salbutamol sulfate | 200microgram/1ml + 1mg/1ml |  | 3010400 |
| 14561 | Salbutamol 400microgram / Beclometasone 200microgram inhalation powder capsules | Salbutamol sulfate/Beclometasone dipropionate | 400microgram + 200microgram |  | 0 |
| 16625 | Ventide Rotacaps (GlaxoSmithKline UK Ltd) | Salbutamol sulfate/Beclometasone dipropionate | 400microgram + 200microgram |  | 0 |
| 18456 | Salbutamol 200microgram / Beclometasone 100microgram inhalation powder capsules | Salbutamol sulfate/Beclometasone dipropionate | 200microgram + 100microgram |  | 0 |
| 18484 | Ventide Paediatric Rotacaps (GlaxoSmithKline UK Ltd) | Salbutamol sulfate/Beclometasone dipropionate | 200microgram + 100microgram |  | 0 |
| 1801 | Ventide inhaler (GlaxoSmithKline UK Ltd) | Salbutamol/Beclometasone dipropionate | 100microgram/1dose + 50microgram/1dose |  | 0 |
| 11307 | Salbutamol 100micrograms/dose / Beclometasone 50micrograms/dose inhaler | Salbutamol/Beclometasone dipropionate | 100microgram/1dose + 50microgram/1dose |  | 0 |
| 5558 | Salmeterol 50micrograms with fluticasone 500micrograms CFC free inhaler | Salmeterol Xinafoate/Fluticasone Propionate | 50micrograms+ 500micrograms/inhalation |  | 03010101/03020000 |
| 5864 | Salmeterol 25micrograms with fluticasone 250micrograms CFC free inhaler | Salmeterol Xinafoate/Fluticasone Propionate | 25micrograms + 250micrograms/actuation |  | 03010101/03020000 |
| 5942 | Salmeterol 50micrograms with fluticasone 250micrograms CFC free inhaler | Salmeterol Xinafoate/Fluticasone Propionate | 50micrograms + 250micrograms/inhalation |  | 03010101/03020000 |
| 6569 | Salmeterol 25micrograms with fluticasone 125micrograms CFC free inhaler | Salmeterol Xinafoate/Fluticasone Propionate | 25micrograms + 125micrograms/actuation |  | 03010101/03020000 |
| 6616 | Salmeterol 25micrograms with fluticasone 50micrograms CFC free inhaler | Salmeterol Xinafoate/Fluticasone Propionate | 25micrograms + 50micrograms/actuation |  | 03010101/03020000 |
| 6938 | Salmeterol 50micrograms with fluticasone 100micrograms dry powder inhaler | Salmeterol Xinafoate/Fluticasone Propionate | 50micrograms + 100micrograms/inhalation |  | 03010101/03020000 |
| 61280 | Seretide 250 Accuhaler (Waymade Healthcare Plc) | Salmeterol xinafoate/Fluticasone propionate | 50microgram/1dose + 250microgram/1dose |  | 03010101/03020000 |
| 63945 | Seretide 250 Accuhaler (Lexon (UK) Ltd) | Salmeterol xinafoate/Fluticasone propionate | 50microgram/1dose + 250microgram/1dose |  | 03010101/03020000 |
| 49868 | Fluticasone 250micrograms/dose / Formoterol 10micrograms/dose inhaler CFC free |  |  |  | 03010101/03020000 |
| 51209 | Fluticasone 125micrograms/dose / Formoterol 5micrograms/dose inhaler CFC free |  |  |  | 03010101/03020000 |
| 62030 | Beclometasone 100micrograms/dose / Formoterol 6micrograms/dose dry powder inhaler |  |  |  | 03010101/03020000 |
| 65758 | Beclometasone 200micrograms/dose / Formoterol 6micrograms/dose inhaler CFC free |  |  |  | 03010101/03020000 |
| 65894 | Beclometasone 200micrograms/dose / Formoterol 6micrograms/dose dry powder inhaler |  |  |  | 03010101/03020000 |
| 67958 | Budesonide 200micrograms/dose / Formoterol 6micrograms/dose inhaler CFC free |  |  |  | 03010100/03020000 |
| 59899 | Fluticasone furoate 184micrograms/dose / Vilanterol 22micrograms/dose dry powder inhaler |  |  |  | 03010400/03020000 |
| 59439 | Fluticasone furoate 92micrograms/dose / Vilanterol 22micrograms/dose dry powder inhaler |  |  |  | 03010400/03020000 |
| 22225 | BECLOMETHASONE /SALBUTAMOL |  |  |  | 0 |
| 64509 | Tiotropium bromide 2.5micrograms/dose / Olodaterol 2.5micrograms/dose solution for inhalation cartridge with device CFC free | Tiotropium bromide/Olodaterol hydrochloride | 2.5microgram/1dose + 2.5microgram/1dose |  | 3010400 |
| 3838 | SALBUTAMOL 400MCG/BECLOMETH.100MCG R/CAP INH |  |  |  | 0 |

Supplementary Table 42: Smoking medical codes

| **Med code** | **READ Code** | **Clinical term** | **Category (code)** |
| --- | --- | --- | --- |
| 33 | 1371 | Never smoked tobacco | Non smoker (1) |
| 11788 | 1371.11 | Non-smoker | Non smoker (1) |
| 60 | 137L.00 | Current non-smoker | Non smoker (1) |
| 52503 | 13WK.00 | No smokers in the household | Non smoker (1) |
| 98177 | 9kn..00 | Non-smoker annual review - enhanced services administration | Non smoker (1) |
| 12961 | 1377 | Ex-trivial smoker (<1/day) | Ex smoker (2) |
| 12957 | 1378 | Ex-light smoker (1-9/day) | Ex smoker (2) |
| 12955 | 1379 | Ex-moderate smoker (10-19/day) | Ex smoker (2) |
| 12956 | 137A.00 | Ex-heavy smoker (20-39/day) | Ex smoker (2) |
| 12959 | 137B.00 | Ex-very heavy smoker (40+/day) | Ex smoker (2) |
| 12946 | 137F.00 | Ex-smoker - amount unknown | Ex smoker (2) |
| 776 | 137K.00 | Stopped smoking | Ex smoker (2) |
| 26470 | 137N.00 | Ex pipe smoker | Ex smoker (2) |
| 19488 | 137O.00 | Ex cigar smoker | Ex smoker (2) |
| 90 | 137S.00 | Ex smoker | Ex smoker (2) |
| 12878 | 137T.00 | Date ceased smoking | Ex smoker (2) |
| 97210 | 137j.00 | Ex-cigarette smoker | Ex smoker (2) |
| 98447 | 9km..00 | Ex-smoker annual review - enhanced services administration | Ex smoker (2) |
| 72706 | E251300 | Tobacco dependence in remission | Ex smoker (2) |
| 16717 | H310100 | Smokers' cough | Ex or current smoker (3) |
| 72700 | ZV11600 | [V]Personal history of tobacco abuse | Ex or current smoker (3) |
| 12942 | 137..11 | Smoker - amount smoked | Current smoker (4) |
| 12958 | 1372 | Trivial smoker - < 1 cig/day | Current smoker (4) |
| 12941 | 1372.11 | Occasional smoker | Current smoker (4) |
| 12944 | 1373 | Light smoker - 1-9 cigs/day | Current smoker (4) |
| 1878 | 1374 | Moderate smoker - 10-19 cigs/d | Current smoker (4) |
| 3568 | 1375 | Heavy smoker - 20-39 cigs/day | Current smoker (4) |
| 1822 | 1376 | Very heavy smoker - 40+cigs/d | Current smoker (4) |
| 12964 | 137C.00 | Keeps trying to stop smoking | Current smoker (4) |
| 12240 | 137G.00 | Trying to give up smoking | Current smoker (4) |
| 12947 | 137H.00 | Pipe smoker | Current smoker (4) |
| 12943 | 137J.00 | Cigar smoker | Current smoker (4) |
| 12945 | 137M.00 | Rolls own cigarettes | Current smoker (4) |
| 93 | 137P.00 | Cigarette smoker | Current smoker (4) |
| 1823 | 137P.11 | Smoker | Current smoker (4) |
| 12952 | 137Q.00 | Smoking started | Current smoker (4) |
| 12951 | 137Q.11 | Smoking restarted | Current smoker (4) |
| 10558 | 137R.00 | Current smoker | Current smoker (4) |
| 12966 | 137V.00 | Smoking reduced | Current smoker (4) |
| 12965 | 137X.00 | Cigarette consumption | Current smoker (4) |
| 12963 | 137Y.00 | Cigar consumption | Current smoker (4) |
| 12960 | 137Z.00 | Tobacco consumption NOS | Current smoker (4) |
| 12967 | 137a.00 | Pipe tobacco consumption | Current smoker (4) |
| 31114 | 137b.00 | Ready to stop smoking | Current smoker (4) |
| 30423 | 137c.00 | Thinking about stopping smoking | Current smoker (4) |
| 30762 | 137d.00 | Not interested in stopping smoking | Current smoker (4) |
| 41979 | 137e.00 | Smoking restarted | Current smoker (4) |
| 46321 | 137f.00 | Reason for restarting smoking | Current smoker (4) |
| 62686 | 137h.00 | Minutes from waking to first tobacco consumption | Current smoker (4) |
| 10211 | 13p..00 | Smoking cessation milestones | Current smoker (4) |
| 34126 | 13p0.00 | Negotiated date for cessation of smoking | Current smoker (4) |
| 34127 | 13p1.00 | Smoking status at 4 weeks | Current smoker (4) |
| 34374 | 13p2.00 | Smoking status between 4 and 52 weeks | Current smoker (4) |
| 41405 | 13p3.00 | Smoking status at 52 weeks | Current smoker (4) |
| 10898 | 13p4.00 | Smoking free weeks | Current smoker (4) |
| 38112 | 13p5.00 | Smoking cessation programme start date | Current smoker (4) |
| 28886 | 13p6.00 | Carbon monoxide reading at 4 weeks | Current smoker (4) |
| 97643 | 38DH.00 | Fagerstrom test for nicotine dependence | Current smoker (4) |
| 10184 | 67A3.00 | Pregnancy smoking advice | Current smoker (4) |
| 98137 | 67H6.00 | Brief intervention for smoking cessation | Current smoker (4) |
| 74907 | 745H.00 | Smoking cessation therapy | Current smoker (4) |
| 81440 | 745H000 | Nicotine replacement therapy using nicotine patches | Current smoker (4) |
| 85975 | 745H100 | Nicotine replacement therapy using nicotine gum | Current smoker (4) |
| 85247 | 745H200 | Nicotine replacement therapy using nicotine inhalator | Current smoker (4) |
| 89464 | 745H300 | Nicotine replacement therapy using nicotine lozenges | Current smoker (4) |
| 94958 | 745H400 | Smoking cessation drug therapy | Current smoker (4) |
| 91708 | 745Hy00 | Other specified smoking cessation therapy | Current smoker (4) |
| 90522 | 745Hz00 | Smoking cessation therapy NOS | Current smoker (4) |
| 9833 | 8B2B.00 | Nicotine replacement therapy | Current smoker (4) |
| 32572 | 8B3Y.00 | Over the counter nicotine replacement therapy | Current smoker (4) |
| 25106 | 8B3f.00 | Nicotine replacement therapy provided free | Current smoker (4) |
| 67178 | 8BP3.00 | Nicotine replacement therapy provided by community pharmacis | Current smoker (4) |
| 7622 | 8CAL.00 | Smoking cessation advice | Current smoker (4) |
| 41042 | 8CAg.00 | Smoking cessation advice provided by community pharmacist | Current smoker (4) |
| 18573 | 8H7i.00 | Referral to smoking cessation advisor | Current smoker (4) |
| 98245 | 8HBM.00 | Stop smoking face to face follow-up | Current smoker (4) |
| 10742 | 8HTK.00 | Referral to stop-smoking clinic | Current smoker (4) |
| 98154 | 8HkQ.00 | Referral to NHS stop smoking service | Current smoker (4) |
| 66409 | 8I2I.00 | Nicotine replacement therapy contraindicated | Current smoker (4) |
| 63717 | 8I2J.00 | Bupropion contraindicated | Current smoker (4) |
| 24529 | 8I39.00 | Nicotine replacement therapy refused | Current smoker (4) |
| 57639 | 8I3M.00 | Bupropion refused | Current smoker (4) |
| 11356 | 9N2k.00 | Seen by smoking cessation advisor | Current smoker (4) |
| 11527 | 9N4M.00 | DNA - Did not attend smoking cessation clinic | Current smoker (4) |
| 28834 | 9OO..00 | Anti-smoking monitoring admin. | Current smoker (4) |
| 32083 | 9OO..11 | Stop smoking clinic admin. | Current smoker (4) |
| 7130 | 9OO..12 | Stop smoking monitoring admin. | Current smoker (4) |
| 12953 | 9OO1.00 | Attends stop smoking monitor. | Current smoker (4) |
| 40418 | 9OO2.00 | Refuses stop smoking monitor | Current smoker (4) |
| 40417 | 9OO3.00 | Stop smoking monitor default | Current smoker (4) |
| 42722 | 9OO4.00 | Stop smoking monitor 1st lettr | Current smoker (4) |
| 60720 | 9OO5.00 | Stop smoking monitor 2nd lettr | Current smoker (4) |
| 66387 | 9OO6.00 | Stop smoking monitor 3rd lettr | Current smoker (4) |
| 53101 | 9OO7.00 | Stop smoking monitor verb.inv. | Current smoker (4) |
| 58597 | 9OO8.00 | Stop smoking monitor phone inv | Current smoker (4) |
| 63901 | 9OO9.00 | Stop smoking monitoring delete | Current smoker (4) |
| 19485 | 9OOA.00 | Stop smoking monitor.chck done | Current smoker (4) |
| 21637 | 9OOZ.00 | Stop smoking monitor admin.NOS | Current smoker (4) |
| 96992 | 9kc..00 | Smoking cessation - enhanced services administration | Current smoker (4) |
| 98493 | 9kc0.00 | Smoking cessatn monitor template complet - enhanc serv admin | Current smoker (4) |
| 98347 | 9ko..00 | Current smoker annual review - enhanced services admin | Current smoker (4) |
| 6359 | E023.00 | Nicotine withdrawal | Current smoker (4) |
| 32687 | E251.00 | Tobacco dependence | Current smoker (4) |
| 95610 | E251000 | Tobacco dependence, unspecified | Current smoker (4) |
| 70746 | E251100 | Tobacco dependence, continuous | Current smoker (4) |
| 68658 | E251z00 | Tobacco dependence NOS | Current smoker (4) |
| 61905 | Eu17.00 | [X]Mental and behavioural disorder due to use of tobacco | Current smoker (4) |
| 56144 | Eu17100 | [X]Mental and behav dis due to use of tobacco: harmful use | Current smoker (4) |
| 9045 | ZG23300 | Advice on smoking | Current smoker (4) |
| 63666 | ZRBm200 | Fagerstrom test for nicotine dependence | Current smoker (4) |
| 63299 | ZRBm211 | FTND - Fagerstrom test for nicotine dependence | Current smoker (4) |
| 47273 | ZRaM.00 | Motives for smoking scale | Current smoker (4) |
| 91513 | ZRao.00 | Occasions for smoking scale | Current smoker (4) |
| 59866 | ZRh4.00 | Reasons for smoking scale | Current smoker (4) |
| 49418 | ZRh4.11 | RFS - Reasons for smoking scale | Current smoker (4) |
| 12954 | ZV4K000 | [V]Tobacco use | Current smoker (4) |
| 35055 | ZV6D800 | [V]Tobacco abuse counselling | Current smoker (4) |

Supplementary Table 43: Smoking cessation medication product codes (patients with a prescription for smoking cessation medication were classified as current smokers)

| **Product code** | **Formulation** | **Route of administration** | **BNF code** |
| --- | --- | --- | --- |
| 42221 | Lozenge | Oromucosal | 4105100 |
| 1703 | Transdermal patch | Transdermal | 4105100 |
| 41765 | Medicated chewing-gum | Oromucosal | 4105100 |
| 5502 | Transdermal patch | Transdermal | 4105100 |
| 37716 | Lozenge | Oromucosal | 4105100 |
| 39123 | Transdermal patch | Transdermal | 4105100 |
| 40683 | Transdermal patch | Transdermal | 4105100 |
| 33392 | Transdermal Patch | Transdermal | 4105100 |
| 58034 |  |  | 4105100 |
| 65968 | Inhalation vapour | Oromucosal | 4105100 |
| 48620 | Inhalation vapour | Oromucosal | 4105100 |
| 38958 | Lozenge | Oromucosal | 4105100 |
| 41809 | Medicated chewing-gum | Oromucosal | 4105100 |
| 5606 | Transdermal patch | Transdermal | 4105100 |
| 5320 | Inhalation vapour | Oromucosal | 4105100 |
| 7303 | Transdermal patch | Transdermal | 4105100 |
| 41040 | Tablet | Sublingual | 4105100 |
| 5784 | Lozenge | Oromucosal | 4105100 |
| 42286 | Lozenge | Oromucosal | 4105100 |
| 42016 | Medicated chewing-gum | Oromucosal | 4105100 |
| 57731 | Liquid | Route of administration not applicable | 4105100 |
| 3818 | Transdermal patch | Transdermal | 4105100 |
| 42047 | Medicated chewing-gum | Oromucosal | 4105100 |
| 27411 | Tablet | Oral | 4105100 |
| 5457 | Transdermal patch | Transdermal | 4105100 |
| 4717 | Transdermal patch | Transdermal | 4105100 |
| 61777 | Medicated chewing-gum | Oromucosal | 4105100 |
| 41860 | Sublingual Tablet | Sublingual | 4105100 |
| 62246 | Medicated chewing-gum | Oromucosal | 4105100 |
| 41376 | Transdermal patch | Transdermal | 4105100 |
| 27414 | Tablet | Oral | 4105100 |
| 2876 | Medicated chewing-gum | Oromucosal | 4105100 |
| 67143 | Lozenge | Oromucosal | 4105100 |
| 35035 | Tablet | Oral | 4105100 |
| 5515 | Lozenge | Oromucosal | 4105100 |
| 54102 | Transdermal patch | Transdermal | 4105100 |
| 4704 | Transdermal patch | Transdermal | 4105100 |
| 66614 | Transdermal patch | Transdermal | 4105100 |
| 41425 | Medicated chewing-gum | Oromucosal | 4105100 |
| 46717 | Inhalation vapour | Oromucosal | 4105100 |
| 40617 | Transdermal patch | Transdermal | 4105100 |
| 41778 | Medicated chewing-gum | Oromucosal | 4105100 |
| 27410 | Film Coated Tablets | Oral | 4105100 |
| 58675 | Transdermal patch | Transdermal | 4105100 |
| 25510 | Medicated Chewing-gum | Oromucosal | 4105100 |
| 41879 | Medicated chewing-gum | Oromucosal | 4105100 |
| 37646 | Lozenge Sugar-free | Oromucosal | 4105100 |
| 60236 | Transdermal patch | Transdermal | 4105100 |
| 49204 | Not applicable | Route of administration not applicable | 4105100 |
| 3404 | Transdermal patch | Transdermal | 4105100 |
| 41372 | Transdermal patch | Transdermal | 4105100 |
| 66285 | Transdermal patch | Transdermal | 4105100 |
| 46592 | Inhalation vapour | Oromucosal | 4105100 |
| 10623 | Transdermal patch | Transdermal | 4105100 |
| 45504 | Mouth Spray | Oromucosal | 4105100 |
| 41507 | Transdermal patch | Transdermal | 4105100 |
| 6448 | Transdermal patch | Transdermal | 4105100 |
| 41426 | Transdermal patch | Transdermal | 4105100 |
| 41801 | Medicated chewing-gum | Oromucosal | 4105100 |
| 68621 | Lozenge | Oromucosal | 4105100 |
| 5440 | Transdermal patch | Transdermal | 4105100 |
| 54574 | Medicated chewing-gum | Oromucosal | 4105100 |
| 49901 | Tablet | Oral | 4105100 |
| 11718 | Sublingual tablet | Sublingual | 4105100 |
| 4166 | Medicated chewing-gum | Oromucosal | 4105100 |
| 49607 | Transdermal patch | Transdermal | 4105100 |
| 66778 |  |  | 4105100 |
| 40865 | Lozenge | Oromucosal | 4105100 |
| 41909 | Medicated chewing-gum | Oromucosal | 4105100 |
| 5877 | Sublingual tablet | Sublingual | 4105100 |
| 27311 | Transdermal Patch | Transdermal | 4105100 |
| 41505 | Transdermal patch | Transdermal | 4105100 |
| 49319 | Lozenge | Oromucosal | 4105100 |
| 45429 | Mouth Spray | Oromucosal | 4105100 |
| 5700 | Lozenge | Oromucosal | 4105100 |
| 31939 | Medicated Chewing-gum | Oromucosal | 4105100 |
| 56552 | Medicated chewing-gum | Oromucosal | 4105100 |
| 5944 | Inhalation vapour | Oromucosal | 4105100 |
| 41368 | Transdermal patch | Transdermal | 4105100 |
| 50541 | Not applicable | Route of administration not applicable | 4105100 |
| 39572 | Transdermal patch | Transdermal | 4105100 |
| 67802 | Transdermal patch | Transdermal | 4105100 |
| 6698 | Lozenge | Oromucosal | 4105100 |
| 41881 | Medicated chewing-gum | Oromucosal | 4105100 |
| 66101 | Transdermal patch | Transdermal | 4105100 |
| 5946 | Medicated chewing-gum | Oromucosal | 4105100 |
| 44106 | Lozenge | Oromucosal | 4105100 |
| 9591 | Transdermal patch | Transdermal | 4105100 |
| 41864 | Medicated chewing-gum | Oromucosal | 4105100 |
| 39166 | Transdermal patch | Transdermal | 4105100 |
| 29680 | Transdermal Patch | Transdermal | 4105100 |
| 66460 | Sublingual tablet | Sublingual | 4105100 |
| 41802 | Transdermal patch | Transdermal | 4105100 |
| 1248 | Spray | Nasal | 4105100 |
| 6630 | Lozenge | Oromucosal | 4105100 |
| 10527 | Transdermal patch | Transdermal | 4105100 |
| 36457 | Transdermal patch | Transdermal | 4105100 |
| 40730 | Lozenge | Oromucosal | 4105100 |
| 55417 | Modified-release tablet | Oral | 4105100 |
| 36618 | Transdermal patch | Transdermal | 4105100 |
| 42048 | Lozenge | Oromucosal | 4105100 |
| 27412 | Tablets | Oral | 4105100 |
| 41496 | Spray | Nasal | 4105100 |
| 57417 | Transdermal patch | Transdermal | 4105100 |
| 45603 | Lozenge | Oromucosal | 4105100 |
| 65406 |  |  | 4105100 |
| 41753 | Medicated chewing-gum | Oromucosal | 4105100 |
| 6642 | Medicated chewing-gum | Oromucosal | 4105100 |
| 6018 | Transdermal patch | Transdermal | 4105100 |
| 13048 | Medicated chewing-gum | Oromucosal | 4105100 |
| 36635 | Transdermal patch | Transdermal | 4105100 |
| 49305 | Lozenge | Oromucosal | 4105100 |
| 65765 |  |  | 4105100 |
| 57829 |  |  | 4105100 |
| 5531 | Lozenge | Oromucosal | 4105100 |
| 6593 | Lozenge | Oromucosal | 4105100 |
| 9806 | Lozenge | Oromucosal | 4105100 |
| 39046 | Transdermal patch | Transdermal | 4105100 |
| 41474 | Transdermal patch | Transdermal | 4105100 |
| 467 | Modified-release tablet | Oral | 4105100 |
| 41808 | Medicated chewing-gum | Oromucosal | 4105100 |
| 50487 | Transdermal patch | Transdermal | 4105100 |
| 5659 | Lozenge | Oromucosal | 4105100 |
| 41356 | Sublingual tablet | Sublingual | 4105100 |
| 25516 | Medicated Chewing-gum | Oromucosal | 4105100 |
| 5115 | Modified-release tablet | Oral | 4105100 |
| 41377 | Medicated chewing-gum | Oromucosal | 4105100 |
| 6323 | Medicated chewing-gum | Oromucosal | 4105100 |
| 42011 | Medicated chewing-gum | Oromucosal | 4105100 |
| 7644 | Transdermal patch | Transdermal | 4105100 |
| 46588 | Medicated chewing-gum | Oromucosal | 4105100 |
| 41485 | Transdermal patch | Transdermal | 4105100 |
| 58410 |  |  | 4105100 |
| 46701 | Medicated chewing-gum | Oromucosal | 4105100 |
| 41931 | Medicated chewing-gum | Oromucosal | 4105100 |
| 8571 | Spray | Nasal | 4105100 |
| 49088 | Transdermal patch | Transdermal | 4105100 |
| 25523 | Medicated Chewing-gum | Oromucosal | 4105100 |
| 41493 | Medicated chewing-gum | Oromucosal | 4105100 |
| 6565 | Medicated chewing-gum | Oromucosal | 4105100 |
| 66376 | Lozenge |  | 4105100 |
| 35089 | Tablet | Oral | 4105100 |
| 39521 | Lozenge | Oromucosal | 4105100 |
| 9804 | Transdermal patch | Transdermal | 4105100 |
| 5758 | Medicated chewing-gum | Oromucosal | 4105100 |
| 55590 | Transdermal Patch |  | 4105100 |
| 40620 | Transdermal patch | Transdermal | 4105100 |
| 5479 | Transdermal patch | Transdermal | 4105100 |
| 41779 | Medicated chewing-gum | Oromucosal | 4105100 |

Supplementary Table 44: COPD medical codes

| **Medical code** | **Read code** | **Read term** |
| --- | --- | --- |
| 794 | H32..00 | Emphysema |
| 998 | H3...11 | Chronic obstructive airways disease |
| 1001 | H3...00 | Chronic obstructive pulmonary disease |
| 3243 | H31..00 | Chronic bronchitis |
| 5710 | H3z..00 | Chronic obstructive airways disease NOS |
| 9876 | H38..00 | Severe chronic obstructive pulmonary disease |
| 10802 | H37..00 | Moderate chronic obstructive pulmonary disease |
| 10863 | H36..00 | Mild chronic obstructive pulmonary disease |
| 11287 | 66YM.00 | Chronic obstructive pulmonary disease annual review |
| 14798 | H312100 | Emphysematous bronchitis |
| 15157 | H31x.00 | Chronic bronchitis NOS |
| 16410 | H32yz00 | Other emphysema NOS |
| 21061 | H3y0.00 | Chronic obstruct pulmonary dis with acute lower resp infectn |
| 23492 | H320z00 | Chronic bullous emphysema NOS |
| 26306 | H320.00 | Chronic bullous emphysema |
| 27819 | H312.00 | Obstructive chronic bronchitis |
| 33450 | H32z.00 | Emphysema NOS |
| 37247 | H3z..11 | Chronic obstructive pulmonary disease NOS |
| 40788 | H32y.00 | Other emphysema |
| 44525 | H312z00 | Obstructive chronic bronchitis NOS |
| 45770 | 66Yg.00 | Chronic obstructive pulmonary disease disturbs sleep |
| 45771 | 66Yh.00 | Chronic obstructive pulmonary disease does not disturb sleep |
| 46578 | H321.00 | Panlobular emphysema |
| 54893 | H582.00 | Compensatory emphysema |
| 56860 | H320000 | Segmental bullous emphysema |
| 60188 | H320200 | Giant bullous emphysema |
| 61118 | H310z00 | Simple chronic bronchitis NOS |
| 64721 | H464000 | Chronic emphysema due to chemical fumes |
| 66058 | Hyu3000 | [X]Other emphysema |
| 68066 | H31yz00 | Other chronic bronchitis NOS |
| 68662 | H320100 | Zonal bullous emphysema |
| 70787 | H32y100 | Atrophic (senile) emphysema |
| 93568 | H39..00 | Very severe chronic obstructive pulmonary disease |
| 99536 | H320300 | Bullous emphysema with collapse |

Supplementary Table 45: Hayfever medical codes

| **Medical code** | **Read code** | **Read term** |
| --- | --- | --- |
| 121 | H170.11 | Hay fever - pollens |
| 16134 | H171.14 | Hay fever - other allergen |
| 3798 | H172.11 | Hay fever - unspecified allergen |
| 15248 | H330.13 | Hay fever with asthma |
| 5627 | H330011 | Hay fever with asthma |

Supplementary Table 46: Eczema medical codes

| **Medical code** | **Read code** | **Read term** |
| --- | --- | --- |
| 5395 | A540.00 | Eczema herpeticum - Kaposi's varicelliform eruption |
| 29779 | F4D3000 | Eczematous eyelid dermatitis |
| 6218 | F502411 | Eczema of external ear |
| 5391 | M12..12 | Contact eczema |
| 230 | M12z100 | Eczema NOS |
| 1095 | M12z111 | Discoid eczema |
| 1424 | M12z200 | Infected eczema |
| 3699 | M12z300 | Hand eczema |
| 8994 | M12z400 | Erythrodermic eczema |

Supplementary Table 47: Rhinosinusitis medical codes

| **Medical code** | **Read code** | **Read term** |
| --- | --- | --- |
| 10546 | H13..11 | Chronic rhinosinusitis |
| 94218 | H014.00 | Acute rhinosinusitis |

Supplementary Table 48: Spirometry codes

| **Medical code** | **Read term** |
| --- | --- |
| 6118 | Spirometry |
| 19428 | Chronic obstructive pulmonary disease excluded by spirometry |
| 10336 | Spirometry reversibility |
| 10420 | Spirometry reversibility negative |
| 10492 | Spirometry reversibility positive |
| 10337 | Spirometry screening |
| 29015 | Spirometry |
| 102522 | Post bronchodilator spirometry |
| 26241 | Spirometry indicated |
| 13683 | Referral for spirometry |

Supplementary Table 49: Peak flow codes

| **Medical code** | **Read code** | **Read term** |
| --- | --- | --- |
| 14227 | 3393.11 | Peak flow rate normal |
| 11832 | 3394.11 | Peak flow rate abnormal |
| 84 | 3395 | Peak exp. flow rate: PEFR/PFR |
| 6723 | 3395.11 | PEFR - peak exp. flow rate |
| 1835 | 3395.12 | PFR - peak flow rate |
| 11772 | 3395.13 | Peak flow rate |
| 102669 | 3395000 | Diurnal variation of peak expiratory flow rate |
| 104413 | 3395100 | Peak expiratory flow rate after exercise |
| 14231 | 339A.00 | Peak flow rate before bronchodilation |
| 27135 | 339A.11 | Peak expiratory flow rate before bronchodilation |
| 14232 | 339B.00 | Peak flow rate after bronchodilation |
| 27136 | 339B.11 | Peak expiratory flow rate after bronchodilation |
| 43033 | 339c.00 | Peak expiratory flow rate pre steroids |
| 11090 | 339C.00 | Expected peak flow rate |
| 14236 | 339C.11 | Expected peak expiratory flow rate |
| 43032 | 339d.00 | Peak expiratory flow rate post steroids |
| 14233 | 339D.00 | Best ever peak flow rate |
| 19833 | 339D.11 | Best ever peak expiratory flow rate |
| 14237 | 339E.00 | More than 80% of predicted peak flow rate |
| 41440 | 339E.11 | More than 80% of predicted peak expiratory flow rate |
| 19835 | 339F.00 | 60-80% of predicted peak flow rate |
| 15657 | 339F.11 | 60-80% of predicted peak expiratory flow rate |
| 23238 | 339G.00 | Less than 60% of predicted peak flow rate |
| 14238 | 339g.00 | Serial peak expiratory flow rate |
| 10498 | 339G.11 | Less than 60% of predicted peak expiratory flow rate |
| 14235 | 339H.00 | Predicted peak flow |
| 65378 | 339I.00 | Expected peak flow rate x 50% |
| 10894 | 339J.00 | Optimal peak flow rate |
| 60854 | 339K.00 | Expected peak flow rate x 30% |
| 47463 | 339L.00 | Expected peak flow rate x 80% |
| 14239 | 339n.00 | Serial peak expiratory flow rate abnormal |
| 30704 | 339o.00 | Peak expiratory flow rate measured using EN 13826 device |
| 27142 | 339p.00 | Predicted peak expiratory flow rate using EN 13826 standard |
| 100275 | 339u.00 | Peak inspiratory flow rate |
| 37233 | 339V.00 | Recorded/predicted peak expiratory flow rate ratio |
| 39862 | 339W.00 | Worst peak flow rate |
| 60933 | 339X.00 | Percentage of best ever peak expiratory flow rate |
| 40193 | 339Y.00 | Percentage of peak expiratory flow rate variability |
| 12697 | 663S.00 | Peak flow meter at home |
| 28982 | 663T.00 | No peak flow meter at home |
| 46658 | 66Y1.00 | Peak expiratory flow rate - technique poor |
| 47053 | 66Y2.00 | Peak expiratory flow rate - technique moderate |
| 25792 | 66Y3.00 | Peak expiratory flow rate - technique good |
| 46656 | 66Y6.00 | Peak expiratory flow rate - compliance good |
| 61417 | 66Y7.00 | Peak expiratory flow rate - compliance moderate |
| 46657 | 66Y8.00 | Peak expiratory flow rate - compliance poor |
| 89024 | 66Yc.00 | Number of cons days less than 80% peak expiratory flow rate |
| 57485 | 66YN.00 | Peak expiratory flow rate compliance |
| 56997 | 66YO.00 | Peak expiratory flow rate technique |
| 11668 | 66YX.00 | Peak expiratory flow rate monitoring |
| 11671 | 66YY.00 | Peak expiratory flow rate monitoring using diary |
| 67724 | 745C000 | Measurement of peak expiratory flow rate |
| 70070 | 745G100 | Education for peak flow technique |

Supplementary Table 50: Codes used to identify participation in clinical trials

| **Med-code** | **Read code** | **Read term** |
| --- | --- | --- |
| 106765 | 8AE..00 | Research study observation activity |
| 108370 | 8AE0.00 | Clinical trial observation activity |
| 86813 | 9Nd8.00 | Patient consented to randomised clinical trial |
| 10255 | 9P…00 | Clinical trial administration |
| 8676 | 9P1..00 | Clinical drug tirals |
| 18491 | 9P11.00 | Patient entered into trial |
| 109318 | 9P11000 | Entered into drug clinical trial |
| 8629 | 9P12.00 | Patient withdrawn from trial |
| 41886 | 9P3..00 | Clinical drug trial follow up visit |
| 98454 | 9P5..00 | Completed clinical trial |
| 98377 | 9P6..00 | Initial assessment for clinical trial |
| 106749 | 9PC..00 | Patient consented to clinical trial |
| 107882 | 9PE..00 | Consent given to review medical records in clinical trial |
| 110637 | 9PF..00 | Entered into device clinical trial |
| 21704 | 9PZ..00 | Clinical trial admin. NOS |
| 17609 | 9Q1..00 | MRC coronary prevention trial |
| 107169 | 9Q12.00 | MRC treatment phase |
| 27508 | 9Q1Z.00 | MRC coronary trial NOS |
| 8571 | 9Q2..00 | Patient in local study |
| 5138 | 9Q21.00 | Patient in asthma study |
| 24697 | 9Q22.00 | Patient in heart disease study |
| 68061 | 9Q22.12 | Patient in heart study |
| 9149 | 9Q2Z.00 | Patient in local study NOS |
| 105883 | 9Q5..00 | Participant Randomised Evaluation Effective Acceptable Computer Therapy Trial |
| 106293 | 9QC..00 | Participant in research study |
| 107500 | 9QD..00 | Withdrwan from research study |
| 107062 | 9QE..00 | Patient study follow-up |
| 106893 | 9QF..00 | Participation in research study completed |
| 106687 | 9QL..00 | Consent given to participate in research study |
| 107384 | 9QM..00 | Consent given to review medical record in research study |

# Supplementary Notes

The following code was used to define treatment steps.

***Children (5 ≤ age ≤ 12)***

**If** *(Long-term and continuous use of OCS)* **then** step = "Step 5"

**Else if** *(High-dose ICS)* **or** *(High-dose ICS/LABA)* **then** step = "Step 4"

**Else if** *(Low/Medium-dose ICS/LABA)*

**Or** *((Low/Medium-dose ICS/LABA)*

**Or** *(Low/Medium-dose ICS* **and** *(LABA* ***or*** *LTRA* ***or*** *Theophylline)))*

**Then** step = "Step 3"

**Else if** *(Low-dose ICS)* **then** step = "Step 2"

**Else if** (*SABA)* **then** step = "Step 1"

**Else**: “Undefined/ not covered by BTS/SIGN guidelines”

***Adults (age > 12)***

**If** *(Long-term and continuous use of OCS)* **then** step = "Step 5"

**Else if** *(High-dose ICS/LABA)*

**Or** *((High-dose ICS)* **and** *(LABA* **or** *LAMA* **or** *LTRA* **or** *Theophylline* **or** *Chromone))*

**Then** step = "Step 4"

**Else if** *(Low/Medium-dose ICS/LABA)*

**Or** *((Low/Medium-dose ICS)* **and** *(LABA* **or** *LTRA* **or** *Theophylline))*

**Then** step = "Step 3"

**Else if** *(Low/Medium-dose ICS)*

**And No** *(LABA* **or** *LAMA* **or** *LTRA* **or** *Theophylline* **or** *Chromone)*

**And No** *(ICS/LABA)*

**Then** step = "Step 2"

**Else if** *(SABA)* then step = "Step 1"

**Else**: “Undefined/ not covered by BTS/SIGN guidelines”

***Treatment steps between September 2006 and the 1st Asthma treatment***

**If** the patient was diagnosed with asthma before 2006 **Then** step = “Step 0”

**Else if** the patient was diagnosed with asthma during the study period (2006-2016) **Then:**

Steps between 2006 and the patient enrolment date = “Censored”

Steps between 2006 and 1^st^ diagnosis date = “Undiagnosed”

Steps between the 1^st^ diagnosis date and the 1^st^ Asthma prescription = “Step 0”

***Correction of cases not covered by BTS/SIGN***

In Adults (age >12): **If** (*high dose ICS)* **and** **No** add-ons (*LABA* **or** *LAMA* **or** *LTRA* **or** *Theophylline* **or** *Chromone*) **Then** Step = ”Step 4”

In Adults and children:

**If** (*Low/Medium-dose ICS)* **and No** add-ons *(LABA* **or** *LTRA* **or** *Theophylline)* **Then** step = “Step 3”

**If** add-ons prescribed alone (*LABA* **or** *LAMA* **or** *Theophylline* **or** *LTRA* **or** *Chromone*) **and No** *ICS* **Then**

**If** (*High-dose ICS in the previous/Next step)* **Then** step = “Step 4”

**Else if** (*Low/Medium-dose ICS in the previous/Next step)* **Then** step = “Step 3”

**Else** step = “Step 0”

**Else if** *(Low/Medium-dose ICS)* **and** *(LAMA* **or** *Chromone)* **and** **No** *(LABA* **or** *LTRA* **or** *Theophylline)* **Then** step = “Step 3”

**Else if** *(Medium-dose ICS)* **Then**

**If** add-ons *(LABA* **or** *LTRA* **or** *Theophylline in the previous /Next step)* **Then** step = “Step 3”

**Else** step = “Step 2”

**Else:** step = Previous step
